# Supplementary material for: Croaking for haste: How long does it take to describe a frog species since its discovery?
Source: PLoS One. 2026 Jan 23;21(1):e0323855. doi: 10.1371/journal.pone.0323855 (PMC12829843; doi:10.1371/journal.pone.0323855)

S5 Fig. Heatmap of specimen collections in Ecuador. Heatmap showing the distribution of specimen collection events in Ecuador over time. The x-axis represents the months of the year, while the y-axis represents the years of collection. The color intensity indicates the number of specimens collected in each time period.

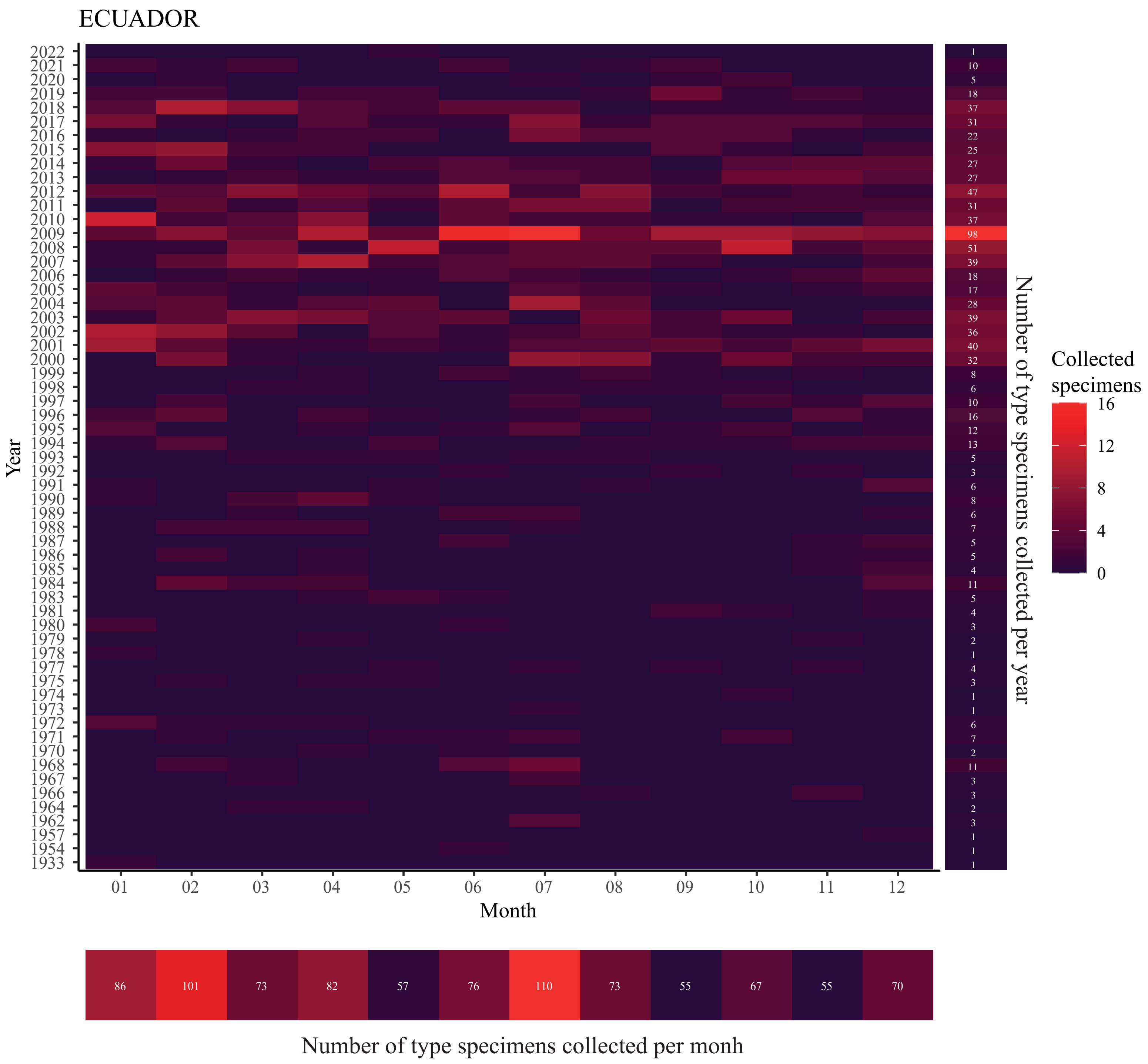

Supplement: S5 Fig — Heatmap showing the distribution of specimen collection events in Ecuador over time. The x-axis represents the months of the year, while the y-axis represents the years of collection. The color intensity indicates the number of specimens collected in each time period. (PDF) [file pone.0323855.s005.pdf]
