# Supplementary figures and images for: Croaking for haste: How long does it take to describe a frog species since its discovery?
Source: PLoS One. 2026 Jan 23;21(1):e0323855. doi: 10.1371/journal.pone.0323855 (PMC12829843; doi:10.1371/journal.pone.0323855)

S6 Fig. Residual diagnostics from the DHARMa R package for the fitted models.

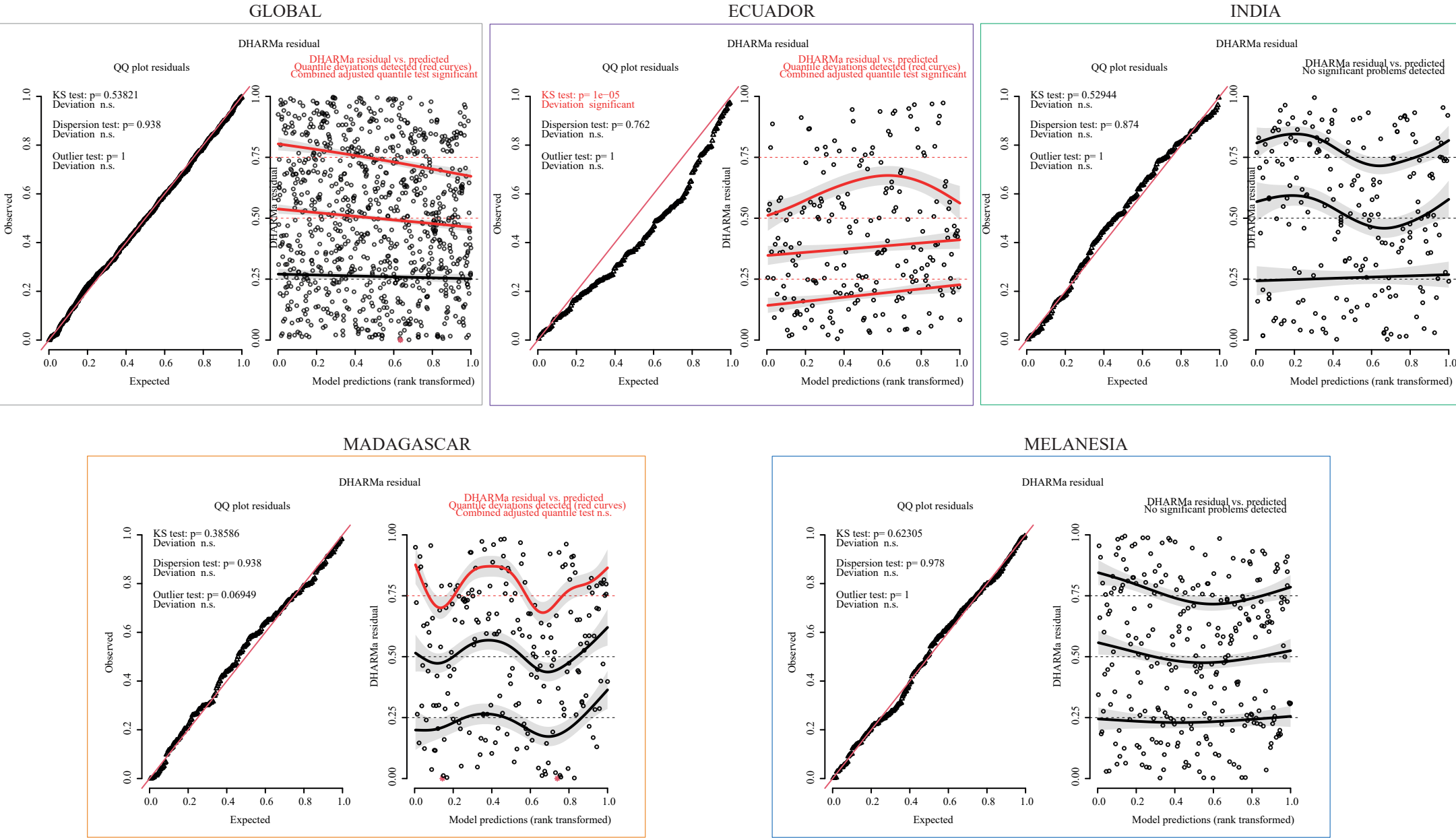

Supplement: S6 Fig — (PDF) [file pone.0323855.s006.pdf]
