# Supplementary material for: Croaking for haste: How long does it take to describe a frog species since its discovery?
Source: PLoS One. 2026 Jan 23;21(1):e0323855. doi: 10.1371/journal.pone.0323855 (PMC12829843; doi:10.1371/journal.pone.0323855)

S7 Fig. Significant effect plots of variables on description sensu stricto. Colored boxes differentiate the regions. Gray= global, Purple = Ecuador, Orange= Madagascar, Green = India, and Blue = Melanesia.

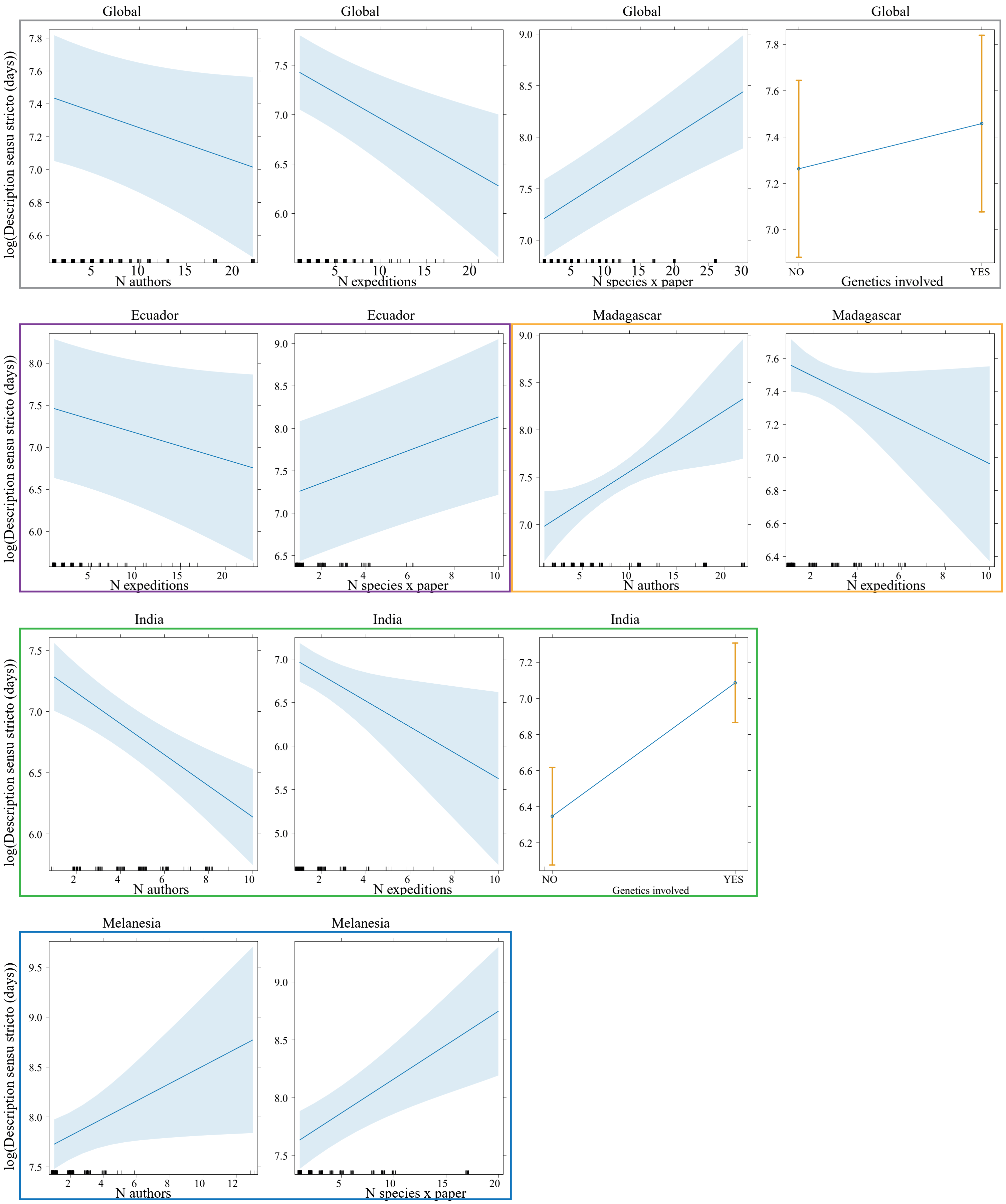

Supplement: S7 Fig — Colored boxes differentiate the regions. Gray = global, Purple = Ecuador, Orange = Madagascar, Green = India, and Blue = Melanesia. (PDF) [file pone.0323855.s007.pdf]
