# Supplementary material for: Croaking for haste: How long does it take to describe a frog species since its discovery?
Source: PLoS One. 2026 Jan 23;21(1):e0323855. doi: 10.1371/journal.pone.0323855 (PMC12829843; doi:10.1371/journal.pone.0323855)

S8 Fig. Temporal evolution of variables in Ecuador. (**a**) Overall description process (black), collection (blue), and description sensu stricto (green), (**b**) Nº of authors in each species description (mean), (**c**) Number of published taxonomic papers (count), (**d**) Taxonomic papers that include genetics (percentage), (**e**) Nº of species described each year (count), (**f**) Nº of species described in each paper (mean), (**g**) Nº of type specimens in the type series (mean), (**h**) Nº of field trips conducted to collect the entire type series (mean).

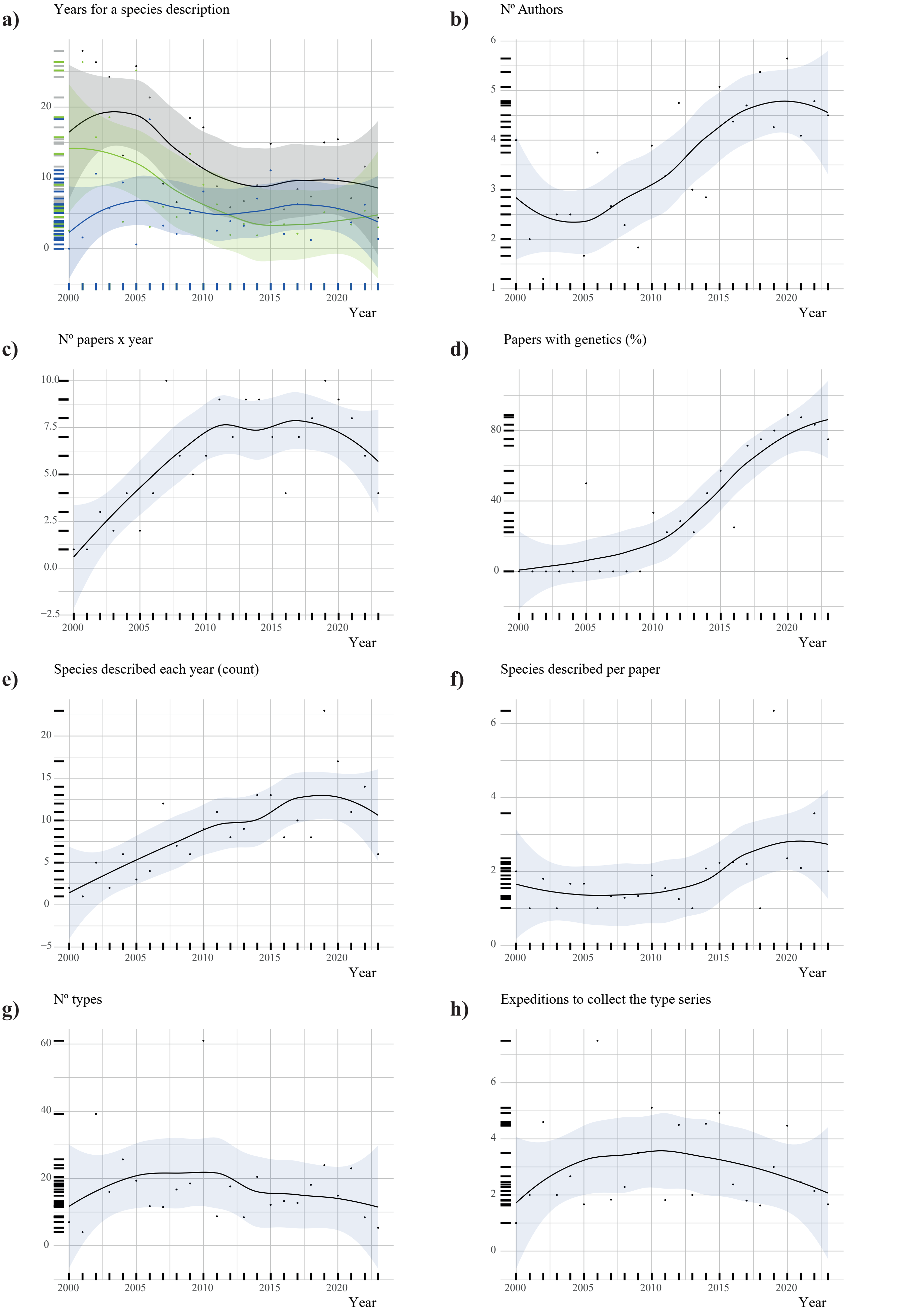

Supplement: S8 Fig — (a) Overall description process (black), collection (blue), and description sensu stricto (green), (b) Nº of authors in each species description (mean), (c) Number of published taxonomic papers (count), (d) Taxonomic papers that include genetics (percentage), (e) Nº of species described each year (count), (f) Nº of species described in each paper (mean), (g) Nº of type specimens in the type series (mean), (h) Nº of field trips conducted to collect the entire type series (mean). (PDF) [file pone.0323855.s008.pdf]
