# Supplementary material for: Croaking for haste: How long does it take to describe a frog species since its discovery?
Source: PLoS One. 2026 Jan 23;21(1):e0323855. doi: 10.1371/journal.pone.0323855 (PMC12829843; doi:10.1371/journal.pone.0323855)
Supplement: S3 Table — (PDF) [file pone.0323855.s014.pdf]

S3 Table. Statistical comparison of AIC values between empiric (fixed + random variables) and null (random variables only) models.

Description *sensu stricto*			
Region	AIC Empiric model	AIC Null model	p-value anova(emp, null)
Global	2315.152	2350.447	**1.634e-08 *****
Ecuador	537.1471	548.0261	**0.000838 *****
India	525.0588	558.4823	**3.856e-08 *****
Madagascar	480.3991	494.3719	**0.0002253 *****
Melanesia	721.8293	728.9007	**0.004045 ****
