# Supplementary material for: Croaking for haste: How long does it take to describe a frog species since its discovery?
Source: PLoS One. 2026 Jan 23;21(1):e0323855. doi: 10.1371/journal.pone.0323855 (PMC12829843; doi:10.1371/journal.pone.0323855)
Supplement: S4 Table — (PDF) [file pone.0323855.s015.pdf]

S4 Table. p-values from the GLMM models and evaluation metrics for global and regional analyses.

Description *sensu stricto*					
Variable p-value	Global	Ecuador	India	Madagascar	Melanesia
N_authors	**0.0868018 .**	0.171589	**1.944e-05 *** **	**0.006475 ** **	**0.0375009 * **
N_types	0.6771016	0.9992508	0.50075	0.929411	0.8609842
Sp_in_genus	0.9097936	0.8682574	0.36596	0.787338	0.2292409
N_expeditions	**0.0006293 *** **	**0.0963995 .**	**0.01378 * **	**0.089970 .**	0.2024911
N_species_x_paper	**7.313e-08 *** **	**0.0003922 *** **	0.76275	0.391804	**0.0002361 *** **
Genetics_involved	**0.0114386 * **	0.7171743	**1.170e-07 *** **	0.258994	0.7497213
MODEL EVALUATION					
Disperstion test	0.938	0.762	0.874	0.938	0.978
testQuantiles	0.02532	2.89E-08	0.2737	0.1469	0.6567
testUniformity	0.5382	6.20E-06	0.5294	0.3859	0.6231
testOutliers	1	1	1	0.06949	1
testZeroInflation	1	1	1	1	1
R^2 only fixed vars.	0.0568	0.0593	0.187	0.149	0.0739
R^2 fixed + Random vars.	0.371	0.524	0.32	0.161	0.229
R^2 improve Random	0.3142	0.4647	0.133	0.012	0.1551
