# Supplementary material for: Croaking for haste: How long does it take to describe a frog species since its discovery?
Source: PLoS One. 2026 Jan 23;21(1):e0323855. doi: 10.1371/journal.pone.0323855 (PMC12829843; doi:10.1371/journal.pone.0323855)
Supplement: Attachment — Submitted filename: 0_Response_to_reviewers.pdf [file pone.0323855.s017.pdf]

MS #PONE-D-25-19328 *Croaking for haste: How long does it take to describe a frog species since its discovery?*

Authors' replies to the comments of the Editor and Reviewers

We thank the editor and reviewers for their positive and constructive comments. We have followed almost all of these. We reply to each comment in the following pages and explain how we dealt with them during the manuscript revision. Please, find below our responses (in black) to the editor's and reviewers' comments (in blue). Each response is preceded by "---Response" for easy identification. All changes in the newly uploaded manuscript have been highlighted using track changes. We hope that the manuscript is now suitable for publication in PLOS ONE.

PLOS ONE

Dear Dr. De la Riva,

Thank you for submitting your manuscript to PLOS ONE. After careful consideration, we feel that it has merit but does not fully meet PLOS ONE's publication criteria as it currently stands. Therefore, we invite you to submit a revised version of the manuscript that addresses the points raised during the review process.

Please submit your revised manuscript by Aug 11 2025 11:59PM. If you will need more time than this to complete your revisions, please reply to this message or contact the journal office at plosone@plos.org. When you're ready to submit your revision, log on to https://www.editorialmanager.com/pone/ and select the 'Submissions Needing Revision' folder to locate your manuscript file. Please include the following items when submitting your revised manuscript: A rebuttal letter that responds to each point raised by the academic editor and reviewer(s). You should upload this letter as a separate file labeled 'Response to Reviewers'. A marked-up copy of your manuscript that highlights changes made to the original version. You should upload this as a separate file labeled 'Revised Manuscript with Track Changes'. An unmarked version of your revised paper without tracked changes. You should upload this as a separate file labeled 'Manuscript'.

If you would like to make changes to your financial disclosure, please include your updated statement in your cover letter. Guidelines for resubmitting your figure files are available below the reviewer comments at the end of this letter. If applicable, we recommend that you deposit your laboratory protocols in protocols.io to enhance the reproducibility of your results. Protocols.io assigns your protocol its own identifier (DOI) so that it can be cited independently in the future. For instructions see: https://journals.plos.org/plosone/s/submission-guidelines#loc-laboratory-protocols. Additionally, PLOS ONE offers an option for publishing peer-reviewed Lab Protocol articles, which describe protocols hosted on protocols.io. Read more information on sharing protocols at https://plos.org/protocols?utm_medium=editorial-email&utm_source=authorletters&utm_campaign=protocols.

We look forward to receiving your revised manuscript.

Kind regards,

Alex Slavenko

Academic Editor

PLOS ONE

Journal Requirements:

1. When submitting your revision, we need you to address these additional requirements.

Please ensure that your manuscript meets PLOS ONE's style requirements, including those for file naming. The PLOS ONE style templates can be found at

https://journals.plos.org/plosone/s/file?id=wjVg/PLOSOne_formatting_sample_main_body.pdf and

https://journals.plos.org/plosone/s/file?id=ba62/PLOSOne_formatting_sample_title_authors_affiliations.pdf

---Response: We have checked and our files meet PLOS ONE'S style requirements.

2. Thank you for stating the following financial disclosure: AC was funded by MCIN/AEI/10.13039/501100011033, contract for industrial doctorates aid DIN2021-011964.

Please state what role the funders took in the study. If the funders had no role, please state: "The funders had no role in study design, data collection and analysis, decision to publish, or preparation of the manuscript." If this statement is not correct you must amend it as needed. Please include this amended Role of Funder statement in your cover letter; we will change the online submission form on your behalf.

---Response: We have added the suggested information and included it at the end of the cover letter.

--- Before it read: AC was funded by MCIN/AEI/10.13039/501100011033, contract for industrial doctorates aid DIN2021-011964.

--- Now it reads: AC was funded by MCIN/AEI/10.13039/501100011033, contract for industrial doctorates aid DIN2021-011964. The funders had no role in study design, data collection and analysis, decision to publish, or preparation of the manuscript.

3. When completing the data availability statement of the submission form, you indicated that you will make your data available on acceptance. We strongly recommend all authors decide on a data sharing plan before acceptance, as the process can be lengthy and hold up publication timelines. Please note that, though access restrictions are acceptable now, your entire data will need to be made freely accessible if your manuscript is accepted for publication. This policy applies to all data except where public deposition would breach compliance with the protocol approved by your research ethics board. If you are unable to adhere to our open data policy, please kindly revise your statement to explain your reasoning and we will seek the editor's input on an exemption. Please be assured that, once you have provided your new statement, the assessment of your exemption will not hold up the peer review process.

---Response: Perhaps we selected this option in error. All data were already included in the initial submission and were uploaded as supplementary files.

4. We note that Figure 1a in your submission contain map images which may be copyrighted. All PLOS content is published under the Creative Commons Attribution License (CC BY 4.0), which means that the manuscript, images, and Supporting Information files will be freely available online, and any third party is permitted to access, download, copy, distribute, and use these materials in any way, even commercially, with proper attribution. For these reasons, we cannot publish previously copyrighted maps or satellite images created using proprietary data, such as Google software (Google Maps, Street View, and Earth). For more information, see our copyright guidelines: http://journals.plos.org/plosone/s/licenses-and-copyright.

We require you to either present written permission from the copyright holder to publish these figures specifically under the CC BY 4.0 license, or remove the figures from your submission:

a. You may seek permission from the original copyright holder of Figure 1a to publish the content specifically under the CC BY 4.0 license.

We recommend that you contact the original copyright holder with the Content Permission Form (http://journals.plos.org/plosone/s/file?id=7c09/content-permission-form.pdf) and the following text:

"I request permission for the open-access journal PLOS ONE to publish XXX under the Creative Commons Attribution License (CCAL) CC BY 4.0 (http://creativecommons.org/licenses/by/4.0/). Please be aware that this license allows unrestricted use and distribution, even commercially, by third parties. Please reply and provide explicit written permission to publish XXX under a CC BY license and complete the attached form."

Please upload the completed Content Permission Form or other proof of granted permissions as an "Other" file with your submission.

In the figure caption of the copyrighted figure, please include the following text: “Reprinted from [ref] under a CC BY license, with permission from [name of publisher], original copyright [original copyright year].”

b. If you are unable to obtain permission from the original copyright holder to publish these figures under the CC BY 4.0 license or if the copyright holder’s requirements are incompatible with the CC BY 4.0 license, please either i) remove the figure or ii) supply a replacement figure that complies with the CC BY 4.0 license. Please check copyright information on all replacement figures and update the figure caption with source information. If applicable, please specify in the figure caption text when a figure is similar but not identical to the original image and is therefore for illustrative purposes only.

The following resources for replacing copyrighted map figures may be helpful:

USGS National Map Viewer (public domain): http://viewer.nationalmap.gov/viewer/

The Gateway to Astronaut Photography of Earth (public domain): http://eol.jsc.nasa.gov/sseop/clickmap/

Maps at the CIA (public domain): https://www.cia.gov/library/publications/the-world-factbook/index.html and https://www.cia.gov/library/publications/cia-maps-publications/index.html

NASA Earth Observatory (public domain): http://earthobservatory.nasa.gov/

Landsat: http://landsat.visibleearth.nasa.gov/

USGS EROS (Earth Resources Observatory and Science (EROS) Center) (public domain): http://eros.usgs.gov/#

Natural Earth (public domain): http://www.naturalearthdata.com/

---Response: The data we used to create the map comes from (https://public.opendatasoft.com/explore/dataset/world-administrative-boundaries/information/) and is licensed under the Open Government Licence v3.0, which is compatible with the Creative Commons Attribution License (CC BY 4.0). We have included this information in the figure caption.

--- Before it read: Time lags and trends in anuran amphibian species descriptions. (a) Cumulative number of frog species described over time. (b) Time lags in the description process. (c) Average description speed per year. Green: description sensu stricto, blue: collection, gray: overall process.

--- Now it reads: Time lags and trends in anuran amphibian species descriptions. (a) Cumulative number of frog species described over time. (b) Time lags in the description process. (c) Average description speed per year. Green: description sensu stricto, blue: collection, gray: overall process. The world map showing country boundaries was obtained from OpenDataSoft (https://public.opendatasoft.com/explore/dataset/world-administrative-boundaries/information/) and is licensed under the Open Government Licence v3.0, which is compatible with the Creative Commons Attribution License (CC BY 4.0).

Additional Editor Comments:

I have now received two reviews for this manuscript. I agree with both reviewers that this manuscript presents an interesting analysis of an impressive dataset, and has some solid results and discussions on a very timely research matter. However, I also share some of reviewer 2's concerns. Some of these are mostly editorial (i.e., the manuscript is quite long and overly detailed in some cases and can be streamlined, as per the excellent suggestion of reviewer 2) and some are related to the content. I agree that some statements made are overly broad and sweeping, and the language can be toned down at times. I also personally have some concerns about advocating fast-track taxonomy and wikification as solutions to the issue. I agree with reviewer 2, also based on my personal experience, that in many cases doing taxonomy properly just takes a whole lot of time, and without investing more resources into there's not much that can be done to speed it up.

---Response: We acknowledge that the manuscript in its original form was quite long and detailed. Following Reviewer #2’s suggestions, we have reduced the length of some sections by focusing on the general patterns, which has shortened the manuscript. Additionally, following the editor’s and Reviewer #2’s suggestions, we have toned down the language throughout the manuscript. Finally, we have placed greater emphasis on the need for increased economic resources for taxonomy.

Overall, I think this is a solid manuscript with a lot of potential, and I encourage the authors to read and adress the reviewer comments carefully if they wish to submit a revised version.

Reviewers' comments:

Reviewer's Responses to Questions

Comments to the Author

1. Is the manuscript technically sound, and do the data support the conclusions?

The manuscript must describe a technically sound piece of scientific research with data that supports the conclusions. Experiments must have been conducted rigorously, with appropriate controls, replication, and sample sizes. The conclusions must be drawn appropriately based on the data presented.

Reviewer #1: Yes

Reviewer #2: Partly

2. Has the statistical analysis been performed appropriately and rigorously?

Reviewer #1: I Don't Know

Reviewer #2: I Don't Know

3. Have the authors made all data underlying the findings in their manuscript fully available?

The PLOS Data policy requires authors to make all data underlying the findings described in their manuscript fully available without restriction, with rare exception (please refer to the Data Availability Statement in the manuscript PDF file). The data should be provided as part of the manuscript or its supporting information, or deposited to a public repository. For example, in addition to summary statistics, the data points behind means, medians and variance measures should be available. If there are restrictions on publicly sharing data—e.g. participant privacy or use of data from a third party—those must be specified.

Reviewer #1: Yes

Reviewer #2: Yes

4. Is the manuscript presented in an intelligible fashion and written in standard English?

PLOS ONE does not copyedit accepted manuscripts, so the language in submitted articles must be clear, correct, and unambiguous. Any typographical or grammatical errors should be corrected at revision, so please note any specific errors here.

Reviewer #1: Yes

Reviewer #2: Yes

5. Review Comments to the Author

Please use the space provided to explain your answers to the questions above. You may also include additional comments for the author, including concerns about dual publication, research ethics, or publication ethics. (Please upload your review as an attachment if it exceeds 20,000 characters)

Reviewer #1: General Assessment:

This manuscript presents a well-conceived and original study based on a remarkable dataset. To the best of my knowledge, it is the first work to quantitatively assess the full duration of the taxonomic description

process—from the initial collection of specimens to the eventual publication of species descriptions. The research addresses an important and timely issue in biodiversity science and has the potential to make a significant contribution to the field.

The authors' approach to categorizing and formalizing their dataset is methodologically rigorous, transparent, and well justified. Their assumptions are clearly detailed and appear reasonable and reproducible. However, as I do not have expertise in statistics, I am not in a position to assess the appropriateness or robustness of the statistical tools and models employed in the analysis.

The discussion is pertinent and well articulated. I find the authors' conclusions and recommendations compelling, especially their suggestions for streamlining the taxonomic workflow to enhance the pace of species inventory. That said, I am not certain that there is a clear consensus within the taxonomic community on some of the proposed recommendations, which highlights the value of this work in stimulating a constructive debate around best practices.

Minor Comments:

I would appreciate it if the authors could briefly explain, in the Materials and Methods section, the meaning and relevance of the statistical indices reported in Table 1 (e.g., Skew, MAD, Kurtosis). For readers who are not trained statisticians, a concise clarification of what each index measures and how it contributes to the analysis would improve accessibility.

---Response: We thank the reviewer for their thoughtful and constructive comments, and for their careful reading of our manuscript.

Some of these metrics are already indirectly described in lines 283–291, where we summarize the values obtained for each (or nearly each) metric. However, we agree with Reviewer #1 that a brief explanation of the metrics would improve clarity. Therefore, we have added this information to the Materials and Methods section.

--- Before it read: To summarize and visualize the three periods, we calculated their durations in years for easier interpretation and representation. We obtained the central tendency and position statistics of each period using the base package in R [43], and we calculated asymmetry and dispersion statistics using the psych package [44]. We used the ggplot2 package [45] to represent the results using violin and boxplots. This process was conducted independently for each genus and region, and merging the four regional datasets to obtain global estimates.

--- Now it reads: To summarize and visualize the three periods, we calculated their durations in years for easier interpretation and representation. Using the base package in R [46], we obtained central tendency statistics including the mean, trimmed mean (mean after excluding 10% of extreme values from both ends to reduce the influence of outliers), and median, along with position statistics such as the minimum and maximum. Using the psych package [47], we then calculated measures of asymmetry, including skewness (an asymmetry coefficient where positive values indicate a long right tail, meaning most observations are concentrated below the mean with some high values) and kurtosis (a measure of tail weight and peakedness where high values indicate heavy tails and a sharp central peak compared to a normal distribution). Using the psych package, we also calculated dispersion measures such as the Standard Deviation (S.D.), Median Absolute Deviation (M.A.D.; a robust dispersion measure; calculated as the median of absolute differences from the median, less sensitive to outliers than S.D.), range, and Standard Error (S.E.). We used the ggplot2 package [48] to represent the results using violin and boxplots. This process was conducted independently for each genus and region, followed by merging the four regional datasets to obtain global estimates.

Conclusion:

In summary, this is a well-executed, well-written, and highly informative article. It presents an original contribution to the literature on taxonomic research. I have no major criticisms and only a single minor suggestion for clarification. I recommend this manuscript for publication after this small revision is addressed.

---Response: Thanks.

Reviewer #2:

This paper is an interesting read that provides information on the temporal distribution of key steps on the road to describing frog species in tropical areas. I think with careful revision it could be worth publishing. I have made some more detailed comments on a word version of the MS, but also make some broader comments here.

Tone of writing - Overall I suggest there may be room to be a bit more tempered in some of the claims made. As examples - for vertebrates (and especially terrestrial verts) it is defendable to argue that we do have moderately good framework for understanding patterns in many areas (i.e the existing framework is not a complete disaster). In developing countries, the claim that naming species leads to their protection is contestable at best, as conservation, let alone conservation of small brown frogs, is low on the priority list. In these places in the short-term individual species focused conservation is also not likely to get off the ground – broader region-based approaches are probably more likely to get traction. This is not to say the taxonomy is not important – it totally is and can feed into the above – but it is not the be all and end all to getting conservation happening in tropical countries. Basically - I suggest temper the tone a little in places.

---Response: We thank the reviewer for his thoughtful and constructive comments, as well as for his careful reading of our manuscript. Below, we respond to both the general comments and the specific remarks provided in the annotated version.

We appreciate the suggestion to temper some of our language and recognize that certain statements may have been more strongly worded than intended. We have reviewed the entire manuscript to identify and revise such instances.

Our intent was to highlight that our understanding of diversity and its spatial patterns remains incomplete and will continue like this until we achieve a complete—or at least representative—picture of actual diversity. In amphibians and reptiles, for instance, with over 100 species from each group described annually, taxonomic knowledge is clearly far from complete, reflecting the dynamic and still-developing nature of our understanding. We agree that in some regions, particularly for terrestrial vertebrates, a relatively well-developed framework exists for recognizing broad biodiversity patterns. However, regions such as Melanesia, Madagascar, and other tropical areas—where hundreds of species have been described recently and many more remain undescribed—demonstrate that substantial portions of biodiversity are still undocumented, and that our understanding is still improving and will continue to shift with further discoveries. We have revised the related text to avoid any misinterpretation.

We agree with the reviewer that taxonomy does not guarantee conservation outcomes and that conservation faces challenges unrelated to taxonomic progress. We do not claim that species descriptions directly lead to conservation, but rather that they are a necessary first step—since we cannot conserve what we do not recognize or delimit. This point has been clarified in the text to avoid misinterpretation. We also acknowledge that species-focused conservation is often impractical in regions with limited resources and low political will, which is why our discussion emphasizes the value of region-based approaches considering the undescribed (but known) species. While charismatic species may occasionally act as flagships for conservation, the documentation of multiple well-justified endemics (e.g., brown frogs) can also support habitat protection, aligning with broader goals such as 30 x 30. Ultimately, as noted, conservation decisions depend on socio-political and economic factors beyond taxonomy alone.

Results – I think these can be rationalised a bit. They are quite long at the moment, but many cases are really just verbal summaries of the tables. Try and cut them down. For instance instead of by region summaries – just say the global trends, and note where regions differ from this. The discussion of months where types where collected is not interesting and should be largely or completely cut. There is a high risk that any differences are largely idiosyncratic so I would strongly suggest focusing on the global trends.

---Response: We have substantially reduced the length of certain sections, focusing on global trends as suggested by Reviewer #2.

--- The section ‘Number of field campaings and temporal patterns of type specimen collection’ has been shortened from 402 to 159 words and now reads:

"Half of the species (476; 53.1%) described between 2000 and 2023 required a single field trip to collect the entire type series, with an average of eight collected specimens per species. Two trips were required for 20.3% of species, three for 11.7%, and four for 6.1%. The remaining ca. 8% were described on the basis of collections made over between five and 23 field trips.

Considering the four regions, species descriptions between 2000 and 2023 were based on type specimens collected between 1893 and 2023, with the majority collected between 1999 and 2016, with some regional differences (S1-S5 Fig).

In Melanesia and Ecuador (S4-S5 Fig), type specimen collection was relatively evenly distributed throughout the year (defined as each month having at least 40% of the number of specimens collected in the peak month). In contrast, Madagascar and India exhibit marked seasonal peaks in collection activity, with certain months showing higher numbers of type specimens collected than others (S2-S3 Fig)."

--- The section 'Temporal evolution of variables' has been reduced from 827 to 643 words, following the reviewer's suggested rationale to focus on the most informative and broadly relevant variables.

Discussion:

Following from the above the discussion is my opinion too long, and includes a lot of general thoughts that are not well connected with the key results and/or quite sweeping. I would suggest this needs a thorough rewrite to make it much more focused.

I would also strongly suggest not framing this around crisis and solution – we have been hearing this for 20 years now (at least) and it has not achieved a great deal. For what it is worth, here is my summary based on working in Melanesia, which likely has over 800 frog species and about 5 active taxonomists (none of which are paid full time to be taxonomists). First there is not going to be a magic bullet, my uni does see describing frogs as a priority activity for me, and neither do most people in Melanesia. That said I do think it is of great value and agree with the authors. So what we can do however is look at the resources we have, the shape the job to be done, and try and prioritise things on criteria such as which things are likely to be most threatened, which descriptions are easiest, and which descriptions will have most impact. If we do this smart we may then be able to attract support to keep working on the job. When people are really keen to work in this space we can support and enable them, while trying to be honest about their job prospects, but we also can't engineer a trained and employed New Guinea frog taxonomist out of thin air. I am not against new technologies at all, but with 5 people in the space (three which are over 60) we have to be realistic about what we can get done. I would suggest a more focused discussion about how to prioritise resources and potentially to advocate for a few more could be a useful thing that can be done here.

---Response: We appreciate the reviewer's thoughtful comments and their candid reflection based on first-hand experience in Melanesia. We have taken this feedback seriously and revised the discussion to improve the focus and structure. We acknowledge that the discussion section is relatively long, but we believe it is necessary to properly contextualize our findings and the broader implications of our results.

While some of the discussion topics are not directly derived from our quantitative results, they emerge logically from them, and we believe they are important to address in the context of species description processes.

We did not intend to frame the manuscript strictly as a "crisis and solution" narrative. Rather, we aimed to describe and discuss the current state of species description—including its challenges. We fully agree that there is no "magic bullet," and we do not suggest otherwise. However, we believe that strategies and pathways for improvement may help address specific bottlenecks and are, therefore, discussed.

Finally, we understand the reviewer's point about prioritizing species for description. However, in many cases, such prioritization is difficult without first initiating the species delimitation process. Unless a species is highly distinct (e.g., morphologically unique, phylogenetically isolated, or strikingly colored), it is often not possible to anticipate how easily it can be described compared to other undescribed species, its conservation status, the impact of its description, or its broader significance. Therefore, while we agree with the theoretical importance of prioritization, we believe it is not straightforward in practice.

I have noted in the marked up word doc some other cases where sweeping statements are good in principle, but in practice can pose issues, or need to be much more fully fleshed out.

---Response: We have carefully reviewed the marked-up Word document and addressed all the noted instances. Below, you will find our detailed responses to each of the points raised.

Areas for consideration with respect to interpretation and analyses

The time from collection to description is actually shorter than I expected. For me this emphasises that we really need to keep collecting (as we and many other have stated in lit). Most new species are relatively newly collected, indicating that the reference libraries of life (museums) have collections that are far from complete. This point has been made, but given increasing hurdles to fieldwork and vouchering, it does not hurt to make it again. If we don't keep collecting - especially in poorly known areas, we are not gunna get the job done.

---Response: We agree that the fact that a substantial proportion of species were collected only a few years prior to their description is significant and worth highlighting. We have therefore revised the relevant paragraph to further emphasize the importance of continued collecting:

--- Before it read: Only 36% of species described since 2000 were collected within a five-year timeframe, suggesting that new species are rarely identified in the field and that targeted sampling campaigns are uncommon. The remaining species were collected earlier —up to 125 years ago— and described much later. This underscores the importance of public collections as reservoirs of potential new species but, at the same time, it concerns about the lack of resources, time, or interest dedicated to examining the collected material.

--- Now it reads: Thirty-six percent of frog species described since 2000 were named within five years of first collection, highlighting the importance of continued collecting. A substantial proportion of recently described species rely on newly collected material, which underscores the need to maintain active fieldwork, particularly in poorly known areas, at a time when logistical, political, and regulatory barriers to collecting and vouchering are increasing. In contrast, the remaining 64% of species were collected earlier —up to 125 years ago— and described much later. This suggests that new species are not always recognized as such in the field, or, if they are, their formal description is not prioritized. It also reflects that targeted sampling aimed at collecting specific, suspected new species is relatively uncommon. They also support the critical role of public scientific collections as reservoirs of undocumented diversity, while also raising concerns about the lack of resources, time, or interest dedicated to examining the collected material.

Is lag time correlated with the number of active taxonomists? I know in Melanesia there are only four living scientists that have described more than five species in this region. I suggest an analysis of how numbers of active authors may influence description speed. This could also illustrate how few people are working on some diverse faunas which would show how tenuous all this knowledge actually is.

---Response: Thank you for this valuable suggestion. In the first submission, we did include the number of active taxonomists as a variable in our analysis as can be seen in the table S2 ‘N_aut_taxon’.

Defining who qualifies as an “active taxonomist” is not straightforward, as (arguably) any author listed on a species description could be considered one.

To address this, we extracted the names of all authors from taxonomic papers published since 2000 and counted the number of times each name appeared across the dataset. We then defined "active taxonomists" as those whose number of appearances exceeded the mean plus the standard deviation. This threshold allowed us to identify highly recurrent contributors. Using this approach, in Melanesia, we identified S. J. Richards, R. Günther, F. Kraus, P. M. Oliver, and A. Allison as active taxonomists—each appearing at least 20 times in species descriptions after the year 2000. This, we think that seems to align well with ‘active taxonomists’ in that region. However, in regions like Ecuador, this threshold excluded some significant but less active (in the last 20 years) taxonomists, whose expertise and impact are nonetheless important.

When we attempted to include the number of active taxonomists per paper as a variable, we found it to be highly correlated with the total number of authors. Due to this strong correlation, we excluded the "number of active taxonomists" from the final model to avoid redundancy, as both variables would have explained

essentially the same variation. Instead, we retained the total number of authors to also test the "author inflation" hypothesis.

I would suggest the 11.3 year lag is likely an underestimate going forward, as the sample is biased towards frogs that are being described. The frogs that remain undescribed are increasingly, the hard ones – complexes of confusing or morphologically similar species that are difficult to resolve. One could speculate that lag time may increase as we increasingly get stuck on these species. But only put this in if space opens up – more important in my opinion to rationalise and focus discussion around some of the points raised earlier.

---Response: Thank you for this thoughtful observation. We understand the concern that our estimate of an 11.3-year lag may underestimate future delays, especially as some species pending description are likely part of morphologically cryptic or complex groups (i.e., the difficult ones) that may require more time and advanced tools (e.g., molecular data) to resolve. However, our dataset already includes a number of species described since 2000 that belong to such complexes, suggesting that some of these challenges are already reflected in the current lag time. Moreover, many species in our dataset were collected over 50 and up to 125 years ago, reflecting these 'difficult cases' and counterbalancing the inclusion of more recently collected, 'easier to describe' species. We believe this helps capture a range of descriptive difficulties, although we agree that lag times may increase as taxonomic efforts increasingly focus on the more complex portions of biodiversity.

Keywords

Crisis is misspelt, order words alphabetically

---Response: thanks for the observation, we have made the changes.

6. PLOS authors have the option to publish the peer review history of their article (what does this mean?). If published, this will include your full peer review and any attached files.

If you choose "no", your identity will remain anonymous but your review may still be made public.

Do you want your identity to be public for this peer review? For information about this choice, including consent withdrawal, please see our Privacy Policy.

Reviewer #1: No

Reviewer #2: Yes: Paul Oliver

[NOTE: If reviewer comments were submitted as an attachment file, they will be attached to this email and accessible via the submission site. Please log into your account, locate the manuscript record, and check for the action link "View Attachments". If this link does not appear, there are no attachment files.]

While revising your submission, please upload your figure files to the Preflight Analysis and Conversion Engine (PACE) digital diagnostic tool, https://pacev2.apexcovantage.com/. PACE helps ensure that figures meet PLOS requirements. To use PACE, you must first register as a user. Registration is free. Then, login and navigate to the UPLOAD tab, where you will find detailed instructions on how to use the tool. If you encounter any issues or have any questions when using PACE, please email PLOS at figures@plos.org. Please note that Supporting Information files do not need this step.

Reviewer #2 attached file comments:

L63 True for many groups of inverts, probably not true for plants and vertebrates, suggest clarify

---Response: In this paragraph we are talking about global diversity (in general) for which the statement 'largest proportion of biodiversity is comprised by undescribed species' is completely applicable.

L80 There its now a good ref for barcoding suggesting high levels on unrecognised diversity in Melanesia as well - file:///Users/s2984027/Downloads/biogeography-18-001_article-137988_en_1-4.pdf

---Response: Done. We have added this reference in three different sections of the paper.

L93 Often these steps are not linear like this though, there can often be back and forth while working what units are worthy of the designation of 'species"

---Response: We have partly rephrased to include the reviewers' point.

--- Before it read: Regardless of the chosen approach, describing a species involves five steps, each depending on the previous one —the five D's of taxonomy [36]: discovery, delimitation, diagnosis, description and specimen determination (although not necessarily by the same person). None of these steps can be achieved without previous field work.

--- Now it reads: Regardless of the chosen approach, describing a species involves five steps —the five D's of taxonomy [38]: discovery, delimitation, diagnosis, description and specimen determination (although not necessarily by the same person). While these steps are conceptually linear, in practice, the process is often iterative, with researchers revisiting and redefining previous steps as new information emerges or as the species boundaries are reassessed. None of these steps can be achieved without previous field work.

L94 Good point - I like this

---Response: Thanks.

L97 Suggest list some different possible data sources here

---Response: We have added some lines of evidence typically used in herpetology.

--- Before it read: To decide whether a lineage merits to be named, data from different lines of evidence should ideally be gathered, which requires time and cannot always be achieved within a single field campaign.

--- Now it reads: To decide whether a lineage merits to be named, data from different lines of evidence (e.g., morphology, genetics, bioacoustics, ecology) should ideally be gathered, which requires time and cannot always be achieved within a single field trip.

L113 We coined a term for this - 'cryptic extinction risk' which may be useful here - https://link.springer.com/article/10.1007/s10531-022-02412-x You could also cite the "dark extinction" papers here

---Response: We appreciate the suggestion and have now include the reference. While we recognize the relevance of the term "cryptic extinction risk", we have opted to retain the term "silent extinction", given its broader use.

L118 I think reframing these as aims rather than questions reads a bit better

---Response: We have followed the reviewer suggestion and changed the goals from questions to aims.

L135 And largely in practice..

---Response: We appreciate the suggestion. Indeed, we found that only a few taxonomists (sensu lato—that is, authors involved in species descriptions) have participated in species descriptions across more than one country (e.g., R.K. Abraham, A. Fouquet, A. Hamidy, C.R. Hutter, J. Köhler, A. Ohler, and R.A. Pyron), and their involvement in two countries simultaneously is very limited. We have therefore updated the sentence to reflect that the selected regions are taxonomically independent both in principle and largely in practice, as suggested.

--- Before it read: These regions are taxonomically independent and, in principle, do not share taxonomists, which helps minimize regional biases in research practices.

--- Now it reads: These regions are taxonomically independent and, in principle —and largely in practice— do not share taxonomists, which helps control for regional biases in research practices.

L151 There is an ongoing discussion about whether to include the Maluku biota as part of the Melanesian biota. I think either approach is defendable, but you might want to check whether your search terms ending up capturing this region or not

---Response: We did not include the Maluku biota because ASW only allows selection of the "South Moluccas" region, which represents only the southern part of the Maluku Islands. Since this filter does not cover the entire Maluku archipelago, we chose to exclude all Maluku Islands from our analysis. Additionally, using the "South Moluccas" filter yields five species, all described before the year 2000. As our analysis focuses exclusively on species described after 2000, applying or omitting this filter would have no effect on the results.

L157 This is true, but focusing in on Melanesia, it remains true that a vast majority of descriptions are still based largely on morph and calls, so yes we have new tools, but in very poorly known areas, the older approaches often remain very dominate, reflecting the fact that these biotas are still rife wth obviously different frogs

---Response: We agree with Reviewer #2's observation. As our results show, Melanesia currently lags behind in the use of molecular data, with fewer than 40% of species descriptions including genetic data. However, this is likely to change soon, given the recent development of a comprehensive mitochondrial barcoding database for the entire region (Ferreira et al., 2025). Furthermore, we believe that the decision to use molecular data in species descriptions often reflects the author's preference rather than an absolute necessity. Incorporating molecular data does not automatically imply that the species are cryptic relative to their congeners, just as relying solely on morphological traits does not necessarily indicate a larger number of obviously distinct frog species compared to other regions.

L195 Authors discretion here - but I feel like field campaign is a bit dramatic - suggest fieldwork and field trip is fine!

---Response: We have replaced all instances of "field campaign" with "field trip" throughout the manuscript.

L233 This is for the pooled 4 region dataset right, perhaps make that super clear?

---Response: Yes, we have clarified.

--- Before it read: We evaluated the temporal evolution of several variables including the overall description process, the collection, the description sensu stricto…

--- Not it reads: For each region and globally, we evaluated the temporal evolution of several variables including the overall description process, the collection, the description sensu stricto...

L239 Ok this answers my question I think, but perhaps add a clarifying section above as well

---Response: Please, see previous comment.

L241 As written not clear what the percentage is of - percentage of descriptions using genetic evidence?

---Response: We have rephrased.

--- Before it read: In addition, we counted the number of species described each year and the number of species described using genetics to compute the percentage.

--- Not it reads: Additionally, we counted the number of species described each year and the number of those that used genetic data, in order to compute the percentage of species descriptions incorporating genetic evidence per year.

L250 Not a competition - but yay for Melanesia!!! :)

---Response: Haha, yes!

L276 All this is in the table, is possible to just give the verbal summary, and point to the table for the numbers?

---Response: We agree that the essential information is already presented in the table; however, since these represent the main results, we prefer to keep the verbal summary in the text for clarity. That said, we have substantially shortened other sections as suggested by Reviewer #2 to balance the manuscript's length.

L281 I guess Melanesia does not win the competition here!

---Response: Given the points Reviewer #2 raised during this revision, we fully understand that Melanesia does not come out on top in this comparison.

L297 This probably not important, but in a lot of places I feel like one decimal place would be a more meaningful cut-off to use in this paper

---Response: We reviewed the entire manuscript (except the tables and p-values) and reduced the number of decimal places to one for better clarity and consistency.

L303 I will bet a lot go money some of this is an artefact of the fact that this is when universities are not teaching, and lecturers can go into the field - I would be inclined to downplay these monthly patterns somewhat - I think other parts of the paper are more interesting

---Response: Following the recommendation, we have significantly shortened this section (see general Result's comment). While we agree that some of the monthly patterns may be influenced by external factors such as academic calendars, we believe a brief mention still adds useful context regarding seasonal biases in sampling.

L305 These summaries are basically the same as what is in the figures, could consider cutting or further rationilising

---Response: We have significantly reduced the length of this section (see previous comment and general comment).

L317 The main researchers in this region have not had lecturing duties, which may explain the broader spread of collections across the year. Again I don't think this is supper interesting and would suggest cutting.

---Response: We have significantly reduced the length of this section (see previous comment and general comment).

L347 That's an interesting bit of information

---Response: We agree, and we have addressed it in the discussion section.

L350 As above - I found this section to detailed, my strong suggestion would be to go through and rationalise. For instance you could give global trends, but only mention regional trends when they conflict, and otherwise point to the figures

---Response: The section 'Temporal evolution of variables' has been reduced from 827 to 643 words following the reviewer's suggested rationale. However, the evolution of many of these variables is interesting in itself, so it is not straightforward to reduce the length without losing relevant information.

L357 Is this basically saying same thing as first two sentences?

---Response: Yes, we have removed the sentence.

L420 Arguable - EO Wilson was onto this in the 80/90's, Gerald Durrell was writing about it in the 60 and 70's… Could cut these last two sentences, and join with next paragraph

---Response: We agree with the reviewer that awareness of the extinction crisis began decades ago, as reflected in the work of Wilson, Durrell, and others. Our original use of "generation" was meant to refer to all people currently living on Earth. However, we recognize that this wording may have been ambiguous and have rephrased the passage accordingly.

--- Before it read: We are the first generation to fully comprehend that we are in the midst of an extinction event, while the majority of species remain undiscovered and undescribed. We may also be the last generation having the opportunity to explore and document Earth's biodiversity [37, 51].

--- Now it reads: In the past century, we have come to realize that our planet is facing a biodiversity crisis, while the majority of species remain undiscovered and undescribed. Unless we reverse the current trend of

threats, we may be among the last generations with the opportunity to explore and document Earth's biodiversity [39, 54].

L444 Don't follow the logic here, in my experience often the opposite also occurs, things are thought to be new, but careful reveals they are not

---Response: We agree that both scenarios can occur and this sentence could be misinterpreted. We have rephrased the paragraph to improve clarity and avoid overgeneralization. Our point is that if 64% of species were described more than five years after collection, this suggests that many new species are either not recognized in the field or are not prioritized for description. In our view, if a species is clearly recognized as new in the field, it should not take ten years or more for its formal description. We acknowledge that delays can occur for many reasons, but taking a decade (or much more) to describe something identified as new from the outset seems excessive, and the less parsimonious explanation. We have rephrased to make it clearer and to include the crucial role of keep collecting.

--- Before it read: Only 36% of species described since 2000 were collected within a five-year timeframe, suggesting that new species are rarely identified in the field and that targeted sampling campaigns are uncommon. The remaining species were collected earlier —up to 125 years ago— and described much later. This underscores the importance of public collections as reservoirs of potential new species but, at the same time, it concerns about the lack of resources, time, or interest dedicated to examining the collected material.

--- Now it reads: Thirty-six percent of frog species described since 2000 were named within five years of first collection, highlighting the importance of continued collecting. A substantial proportion of recently described species rely on newly collected material, which underscores the need to maintain active fieldwork, particularly in poorly known areas, at a time when logistical, political, and regulatory barriers to collecting and vouchering are increasing. In contrast, the remaining 64% of species were collected earlier —up to 125 years ago— and described much later. This suggests that new species are not always recognized as such in the field, or, if they are, their formal description is not prioritized. It also reflects that targeted sampling aimed at collecting specific, suspected new species is relatively uncommon. They also support the critical role of public scientific collections as reservoirs of undocumented diversity, while also raising concerns about the lack of resources, time, or interest dedicated to examining the collected material.

L455 This is extremely arguable. In the places I work we desperately need both, there are literally 5 people describing species in a fauna that we estimate contains hundreds of candidate species and vast areas of uncollected terrain. Please do not write this as we do not need more people coming up reasons why we can't fill these massive sampling gaps we have. I will also argue that feildtrips often produce the easy to described species that can be done quickly, I would guess part of the slow down for Melanesia is linked back to a slowdown in fieldwork. So this means we are left dealing with the hard slow species, while also failing to fill the massive geographic gaps that remain unsnapled

---Response: Thank you for pointing this out. We fully agree that both increased collecting and increased taxonomic capacity are urgently needed, particularly in regions like Melanesia where the number of candidate species far exceeds the number of people working on them. Our intention was not to discourage collecting efforts, but to emphasize that their full value is only realized when material is properly processed and revised. To avoid misinterpretation, we have reworded the paragraph to clarify this highlighting the need to reverse the funding cuts in taxonomy.

--- Before it read: In the current context of the biodiversity crisis, some authors have suggested prioritizing the collection and preservation of as many specimens as possible as testimonies of a vanishing biodiversity, ensuring future generations can describe species that may already be extinct [37, 59]. However, unless this strategy is paired with thorough revision of collected material, it may result in decades of specimens remaining unexamined on shelves, delaying species descriptions that could contribute to their conservation.

--- Now it reads: In the context of the current biodiversity crisis, some authors have suggested prioritizing the collection and preservation of as many specimens as possible as testimonies of a vanishing biodiversity, ensuring future generations can describe species that may already be extinct [39–63]. Collecting efforts are essential for addressing the geographical and taxonomic gaps that still exist. To maximize their contribution to biodiversity discovery, collecting should be paired with proper specimen processing (including tissue

sampling for genetic analysis) and thorough taxonomic revision to promptly detect candidate species and prioritize their description.

L458 Honestly - this statement is frustrating - we have had 20 years of people talking about how to fix the crisis in taxonomy. The reality here is that it takes time, there is no quick fix, and there are fewer and fewer jobs. We can prioritise the resources we have to try and ensure maximum impact and try and advocate for more resources. But please stop pretending there is some magic fix around the corner, and we need to start talking about how we are going to prioritise the resources we do have to maximise value, rather than pretending we can somehow get it all done in the next 10 years.

---Response: We thank the reviewer for highlighting the complexities in addressing the taxonomy crisis. We have revised the section to emphasize the critical need for increased funding and streamlined methods.

--- Before it read: To ensure species are described while they still exist, it is necessary to adopt streamlined species description methodologies (e.g., "fast-track taxonomy"), the integration of molecular data to support rapid identifications, and increasing funding for taxonomic research.

--- Now it reads: To describe as many species as possible while they still exist, it is essential to reverse decades of funding cuts to taxonomy. Increased investment would support the creation and maintenance of much-needed taxonomic positions, expand sampling efforts, and enable thorough scrutiny of both recent and historical collections. In parallel, adopting streamlined species description methodologies (e.g., "fast-track taxonomy" in the sense explained above) and integrating molecular data to support rapid identifications can improve efficiency of species discovery and descriptions.

L460 Yes molecular data is good, but it is not nessecary in all cases. And this ignores the fact that many countries don't have access to these resources, or won't allow export to specimens to countries that do. So yes in principle, and we should certainly take and lodge tissues, but in practice sweeping statements like this often create issues for situations where they are not feasible to apply in practice.

--- Response: Thank you for this important comment. We have revised the paragraph to acknowledge the value of molecular data while also recognizing the practical limitations of its collection and use.

--- Now it read: Large-scale molecular screenings are still relatively rare, but those conducted have revealed high numbers (even hundreds) of potential new species—enabled by comparisons with previously sequenced taxa [10, 30, 32, 55]. Despite growing use of molecular data in species descriptions over the last decades, it has yet to become a standard practice (see results; [53]). Our results suggest that incorporating molecular data into frog descriptions significantly increases description time in some regions. While adding any procedure to the species descriptions inherently increases the time required, the increase of time can also be attributed to cryptic species, which may remain undescribed on shelves for years until identified molecularly. Sequencing type specimens during species descriptions offers benefits for taxonomy and biodiversity science. DNA-barcodes linked to identified and re-examinable type specimens establish a reliable connection between genetic data and physical vouchers from the outset [66]. This enables unambiguous specimen assignment to newly described species often without taxonomic expertise—except in cases of introgression, hybridization, or human error [67]. Therefore, by leveraging new methodologies and increasingly affordable molecular tools, the widespread use of DNA barcodes to screen collected material should ideally become a standard practice among taxonomists where feasible, especially in poorly surveyed regions [68].

L464 This is simply not true, temper and give context.

---Response: We have revised the sentence to provide additional context and accuracy.

--- Before it reads: Earth's tropical regions remain largely unexplored.

--- Now it reads: Tropical regions harbor most of Earth's terrestrial biodiversity, yet they remain highly threatened and among the least scientifically understood [64–65].

L473 Again good in principle, but in reality barcoding often just highlights moderately divergent lineages that are difficult to interpret and require detail analyses, and often further collections to identify turnover and contact points - see comment above about sampling

---Response: We partially agree with the reviewer. As already stated in the manuscript, “the reliance on barcodes alone does not provide sufficient evidence for robust species delimitation.” Importantly, we consistently refer to these lineages as ‘candidate’ species, not as ‘new’ species. This distinction underscores that such lineages must undergo taxonomic scrutiny to decide if there is enough evidence to name them. Nonetheless, we argue that making genetic data from geographically explicit samples publicly available plays a crucial role in drawing attention to previously unrecognized biodiversity. Highlighting candidate species and making their data publicly available not only fosters future taxonomic assessments but also contributes valuable data for other biological disciplines. While we acknowledge that mitochondrial divergence alone is not definitive, in many cases (with exceptions), deeply divergent mitochondrial lineages do correspond with nuclear differentiation and ultimately result in the recognition of distinct species.

--- Before it read: Genetically characterizing the collected material and making the sequences with specific localities publicly available would not only highlight candidate species, thereby promoting their formal description, but also provide valuable data for other disciplines.

--- Now it reads: Genetically characterizing the collected material and publicly releasing the georeferenced sequences would not only highlight candidate species—thereby promoting their formal description—but also provide valuable data for other disciplines such as biogeography, evolutionary biology, and ecology.

L501 Agreed

---Response: Thanks.

L509 I feel like this paragraph emphasising the need to pragmatism, directly conflicts with the earlier section which states that everything must be barcoded

---Response: We appreciate the reviewer’s concern. We believe there is a clear distinction between the comprehensive inclusion (in the original description) of natural history, biogeography, or osteological data and the inclusion of a minimal genetic marker (e.g., a DNA barcode), which we argue should be considered standard practice, when the sociopolitical context allows it. Including basic genetic information in species descriptions not only facilitates future identifications—especially for non-specialists or early-career taxonomists—but also enables a broad range of downstream analyses across disciplines.

In this sense, our recommendation for including barcodes is not at odds with the call for pragmatism, but rather reflects a practical and scalable baseline that enhances taxonomy. For example, while providing a full osteological description can be scientifically valuable, its utility in future comparative studies (unless osteology is the only line of evidence supporting the species hypothesis) often requires specialized training. On the other hand, sequencing a short gene fragment (e.g., ~300 bp) allows for straightforward comparisons—even by early-stage researchers—and makes the identity of the species more accessible, unless specific complications like mitochondrial introgression are present.

To make this concrete: consider a young zoologist conducting fieldwork in Ecuador who needs to work with the genus *Pristimantis* for the first time. Having access to a reliable barcode database would greatly ease the process of species identification, compared to relying solely on morphological traits, which often require lot of time (comparing many specimens) to get familiar with. We acknowledge that this is partly a matter of perspective, but we strongly believe that incorporating basic genetic data is one of the most effective and pragmatic ways to advance taxonomy in diverse tropical regions.

L516 THis para has some interesting ideas, but does not follow through on them

---Response: The paragraph was intended to raise key questions rather than fully resolve them, as determining minimum standards for species descriptions or deciding how comprehensive they should be is beyond the scope of this manuscript. Our goal was to introduce these unresolved issues as a way of framing the discussion that follows in subsequent sections.

L522 This is true, but it increasingly feels like this discussion is a wide ranging journey through lots of ideas that are not really clearly linked back to the key results of the análisis

---Response: In this paragraph, we introduce a major impediment to taxonomic progress: the limited accessibility of taxonomic data and the lack of standardization when such data are available. This point

directly connects to the following paragraph, where we discuss the potential of cybertaxonomy to address these challenges. While some parts of the discussion are not direct outputs of our quantitative analyses, they emerge logically from our findings and are relevant to the broader context of species description practices. Indeed, the practical difficulties of accessing type specimens, locating data papers, dealing with non-standardized data, or physically traveling to multiple collections are issues likely encountered during the description of many of the ~900 species analyzed in our study. We believe these are critical topics to raise in order to contextualize the structural limitations that affect taxonomic workflows today.

L531 Can we please stop pretending there is a "solution" - we need to look for ways to streamline and speed up the process where possible I agree, but pretending there is some way to magically speed up the rate of species descriptions x10 with existing resources is not really helping anyone. By far then most productive frog taxonomists are sill doing it largely the old-fashioned way (although sometime using newer data types) - they are just more organised, more resourced and/or more efficient than others - I don't see anything changing that in the near future

---Response: We want to clarify that we did not intend to frame the manuscript as a "crisis and solution" narrative. Our goal was to describe and discuss the current state of species description, including its challenges. We fully agree that there is no "magic bullet," and we do not suggest otherwise. However, we believe that some strategies and pathways may help address specific bottlenecks, which is why we included them in the discussion. To reflect this nuance, we have replaced the word "solution" with "strategy" throughout the manuscript.

Regarding the second point, the main goal of this paragraph is to emphasize the need for publicly available and easily reusable data—something that, whether an author uses morphology alone or multiple lines of evidence, is likely to be widely agreed upon.

L538 You would need make the description ICZN compliant (not trivial). Is this a platform just for frogs? Is there a peer-reviewed process involved here? This is an interesting idea - but there is a lot of complexity here.

---Response: We have now included this relevant information in the revised paragraph, which was previously omitted. The scientific work and the naming process will remain ICZN-compliant and follow the standard procedures, including submitting drafts for peer review, editing, and publication. The cybertaxonomy (which could be useful for all taxa, not only for frogs) begins after this traditional process. At that stage, all data associated with the paper should be uploaded in a standardized format to facilitate easy submission—either by the journal or the author—into a publicly accessible platform. Additionally, we have specified that any information uploaded to the platforms should be traceable, either through ORCID-identified users or associated published bibliography.

---The new paragraph now reads: A previously suggested strategy to enhance data accessibility and thereby accelerate species descriptions, without compromising species hypothesis is "wikifying" taxonomic data (i.e., cybertaxonomy [83–86]). This approach integrates traditional taxonomic practices with modern digital platforms. The formal description would continue to follow (unless a better solution is found) established taxonomic procedures (i.e., peer-reviewed publications that comply with the ICZN). However, information about a species or candidate species would be available on a centralized digital platform uploaded by ORCID-identified researchers or through referenced literature. This would draw attention to these undescribed lineages and raise awareness among colleagues working on or possessing information about the same taxa [21, 87–88]. If centralized, this system would liberate taxonomic data from conventional publication formats, make it easy to find all information about a species, and facilitate the continuous updating of species descriptions and data before, during, and after their formal description (e.g., DNA sequences, traits, and distributional data). In addition, it could promote the release of data that, due to their magnitude or nature, are not usually published but are nonetheless of interest (e.g., morphological measurements of already described species). This strategy would allow the mass description of species following a (to be defined) minimum standard to ensure reliable descriptions, with the possibility of later adding information to update them ([21, 53]; and references therein). Consequently, it would not be necessary to delay naming species to provide the most complete description possible. Species could be included in the conservation scheme from the outset, and although the act of naming itself remains static, their descriptions and biological content could remain dynamic and be updated over time. Leveraging the

fact that a small number of journals are responsible for the vast majority of published taxonomic papers, these leading journals—as suggested by Miller et al [86]—could take the lead and coordinate with other journals to request standardized taxonomical data (e.g., morphometric values, variable labelling, coordinates) and routinely upload it to digital platforms. Once a shared minimum standard for reliable descriptions and a standardized data submission format is established, other journals and authors could follow the same templates, ensuring consistency and broad adoption across the field.

L542 Only once someone has gone through and given them a realist status or simailar - even most described species are effectively invisible to the average conservationist, especially in the tropics

---Response: We will likely agree that our world is failing alarmingly to protect biodiversity. However, under the current rules, once a species has been formally described, it becomes possible to take conservation action if the necessary resources and, above all, the political will are in place. Matters such as indifference toward species loss fall outside the scope of this manuscript.

L546 Legislation in some does protect populations or other below species units, so this statement as worded is generally true, not nut correct

---Response: To avoid any misinterpretation, we have removed this sentence. However, we refer specifically to undescribed species that often, prior to formal description, do not qualify even as populations—though in some cases they may be considered divergent populations. Essentially, these entities do not exist until described.

L547 This is fine as you refer the IUCN specifically

---Response: We have removed the previous sentence to specifically refer to the IUCN.

L551 Careful with wording here, it makes it sound like the taxonomist are dithering around unaware of what is happening, in some cases this is true, but in others they are totally aware and desperate to get things names, but just face many constraints on getting the work done

---Response: We have rephrased to avoid any misinterpretation.

--- Before it read: In some cases, taxonomists describe species whose existence has been known (to specialists) for decades, and when they finally name them, they realize that the locality where it was first collected has been completely destroyed

--- Now it reads: In some cases, taxonomists describe species whose existence has been known (to specialists) for decades, but because formal naming can be delayed by multiple external constraints, when they finally name them, they sadly realize that the locality where it was first collected has been completely destroyed

L554 In Melanesia - I would say whether things are named of not does not have a massive impact on their conservation prospects - it might improve them slightly on balance, but other factors are probably more important - like whether the world warms 2 or 4 degrees, or whether chytrid gets into the island or not

--- Response: Melanesia is likely an exception compared to other tropical regions. It harbors immense biodiversity while experiencing low levels of habitat destruction. In many other tropical areas, high biodiversity coincides with extensive deforestation, which creates a different dynamic. In an ideal world, if there is sufficient will and scientific evidence, deforestation can be locally halted, potentially improving conservation outcomes.

L557 I think this is true - but again potentially much less so in tropical regions

---Response: As the reviewer pointed out, protecting populations is indeed viable under certain circumstances; however, it is generally the species level that receives most attention. Issues such as indifference toward species loss, while important, fall outside the scope of this manuscript.

L556 I agree this is an interesting idea. The question would be whether this will be picked up by non-specialists in their work to prioritise regions. One intermediate step here is that taxonomic specialists can

use there knowledge of undescribed diversity to try and map and highlight key areas - See Fred Kraus's work on Milne Bay Province for an example

Response: The purpose of publishing genetic and distributional data on undescribed species is not only to prioritize regions but also to enable a wide range of downstream analyses at different scales. Such data allows other researchers, often using methodologies unfamiliar to taxonomists, to uncover meaningful biodiversity patterns. Once a sequence is available in GenBank and can be geographically attributed to a region, many studies can utilize this data. In fact, it has already been used in macroecological and phylogenetic studies to prioritize taxa and understand biogeographic patterns (see for example; https://www.pnas.org/doi/10.1073/pnas.1714891115, https://onlinelibrary.wiley.com/doi/full/10.1111/jbi.14491, https://www.nature.com/articles/s44185-023-00024-4,).

Reviewer #2 Word Comments

Reviewer 2 made numerous changes to the text. We have accepted the vast majority of them. The changes that were not accepted are discussed below:

L32 new species descriptions accumulate *similar rapidity*. We have not accepted this change because candidate species are flagged by the hundreds, while species descriptions increase at a steady rate of approximately 100 species per year.

L51 Populations *of animals and plants* are being reduced, contracted, and extirpated; species are becoming extinct at an unprecedented rate… We have changed the sentence using 'Wildlife instead of 'animals and plants'.

L102 This is especially true for frogs, where obtaining call recordings —often a source of key diagnostic characters— can be ~~daunting~~ *time-consuming*. The phrase "time-consuming" appears earlier in the text, and we wish to avoid repetition. More importantly, we feel that "daunting" better captures the broader challenges involved in obtaining call recordings—such as logistical difficulties, species-specific behavior, and environmental constraints—which go beyond the issue of time alone.
