# Supplementary material for: Croaking for haste: How long does it take to describe a frog species since its discovery?
Source: PLoS One. 2026 Jan 23;21(1):e0323855. doi: 10.1371/journal.pone.0323855 (PMC12829843; doi:10.1371/journal.pone.0323855)
Supplement: Attachment — Submitted filename: PONE-D-25-19328_R1 Oliver comments.pdf [file pone.0323855.s018.pdf]

PLOS ONE

Croaking for haste: How long does it take to describe a frog species since its discovery?

--Manuscript Draft--

**Manuscript Number:**	PONE-D-25-19328R1
**Article Type:**	Research Article
**Full Title:**	Croaking for haste: How long does it take to describe a frog species since its discovery?
**Short Title:**	Croaking for haste
**Corresponding Author:**	Ignacio Jose De la Riva, Ph.<br>Museo Nacional de Ciencias Naturales<br>Madrid, SPAIN
**Keywords:**	Amphibians; Biodiversity crisis; conservation; Linnean shortfall; Species descriptions; Taxonomy
**Abstract:**	Global biodiversity faces severe anthropogenic threats, with alarming extinction rates projected for the near future. Most of Earth's diversity remains undescribed, meaning countless species are doomed to extinction before being documented. Since current conservation laws typically consider only described species, the lag time in achieving a representative global biodiversity inventory is a crucial issue impeding effective conservation. Amphibians, the most endangered vertebrate class, exemplify the challenge: while the number of threatened species rises, new species descriptions rapidly increase, and hundreds of candidate species are flagged annually. We analyzed all anuran species described from 2000 to 2023 across four biodiversity-rich tropical regions to investigate the time required to describe new frog species. We quantified the time needed to collect the type series, the number and timing of expeditions, the lag between collection and publication, and the total time between first specimen collection and article publication. Additionally, we explored temporal trends and the effect of selected abiotic variables. On average, it takes 11.3 years to formally describe a frog species since the collection of the first specimen, with 4.5 years spent collecting the type series and 6.8 years on description and publication. These figures were consistent across three of the four regions analyzed. Alarmingly, the time required to describe new species is globally increasing, exacerbating the so-called taxonomic impediment. Thirty-six percent of species were named within five years of first collection, highlighting the need for continued collecting, biological collections as reservoirs of undescribed diversity, and calling for specimen revision after expeditions. These results raise concerns about the effectiveness of current taxonomic and conservation practices addressing the biodiversity crisis. We call for a global effort to prioritize taxonomic research and discuss taxonomic and conservation approaches. Under current practices, and given the observed timelines, we will lose the race against extinction for many species.
**Order of Authors:**	Albert Carné
	Alberto Sánchez-Vialas
	Claudia Lansac
	Miriam Moreno
	Ignacio Jose De la Riva, Ph.
**Response to Reviewers:**	We uploaded a formatted PDF ("Response_to_reviewers") with detailed responses to the editor and reviewers. However, we paste the non-formatted version here:<br>MS #PONE-D-25-19328 Croaking for haste: How long does it take to describe a frog species since its discovery?<br><br>Authors' replies to the comments of the Editor and Reviewers<br>We thank the editor and reviewers for their positive and constructive comments. We have followed almost all of these. We reply to each comment in the following pages and explain how we dealt with them during the manuscript revision. Please, find below

Powered by Editorial Manager® and ProduXion Manager® from Aries Systems Corporation

our responses (in black) to the editor's and reviewers' comments (in blue). Each response is preceded by "---Response" for easy identification. All changes in the newly uploaded manuscript have been highlighted using track changes. We hope that the manuscript is now suitable for publication in PLOS ONE.

PLOS ONE
Dear Dr. De la Riva,
Thank you for submitting your manuscript to PLOS ONE. After careful consideration, we feel that it has merit but does not fully meet PLOS ONE's publication criteria as it currently stands. Therefore, we invite you to submit a revised version of the manuscript that addresses the points raised during the review process.
Please submit your revised manuscript by Aug 11 2025 11:59PM. If you will need more time than this to complete your revisions, please reply to this message or contact the journal office at plosone@plos.org. When you're ready to submit your revision, log on to https://www.editorialmanager.com/pone/ and select the 'Submissions Needing Revision' folder to locate your manuscript file. Please include the following items when submitting your revised manuscript: A rebuttal letter that responds to each point raised by the academic editor and reviewer(s). You should upload this letter as a separate file labeled 'Response to Reviewers'. A marked-up copy of your manuscript that highlights changes made to the original version. You should upload this as a separate file labeled 'Revised Manuscript with Track Changes'. An unmarked version of your revised paper without tracked changes. You should upload this as a separate file labeled 'Manuscript'.
If you would like to make changes to your financial disclosure, please include your updated statement in your cover letter. Guidelines for resubmitting your figure files are available below the reviewer comments at the end of this letter. If applicable, we recommend that you deposit your laboratory protocols in protocols.io to enhance the reproducibility of your results. Protocols.io assigns your protocol its own identifier (DOI) so that it can be cited independently in the future. For instructions see: https://journals.plos.org/plosone/s/submission-guidelines#loc-laboratory-protocols. Additionally, PLOS ONE offers an option for publishing peer-reviewed Lab Protocol articles, which describe protocols hosted on protocols.io. Read more information on sharing protocols at https://plos.org/protocols?utm_medium=editorial-email&utm_source=authorletters&utm_campaign=protocols.
We look forward to receiving your revised manuscript.
Kind regards,
Alex Slavenko
Academic Editor
PLOS ONE
Journal Requirements:
1. When submitting your revision, we need you to address these additional requirements.
Please ensure that your manuscript meets PLOS ONE's style requirements, including those for file naming. The PLOS ONE style templates can be found at https://journals.plos.org/plosone/s/file?id=wjVg/PLOSOne_formatting_sample_main_body.pdf and https://journals.plos.org/plosone/s/file?id=ba62/PLOSOne_formatting_sample_title_authors_affiliations.pdf
---Response: We have checked and our files meet PLOS ONE'S style requirements.
2. Thank you for stating the following financial disclosure: AC was funded by MCIN/AEI/10.13039/501100011033, contract for industrial doctorates aid DIN2021-011964.
Please state what role the funders took in the study. If the funders had no role, please state: "The funders had no role in study design, data collection and analysis, decision to publish, or preparation of the manuscript." If this statement is not correct you must amend it as needed. Please include this amended Role of Funder statement in your cover letter; we will change the online submission form on your behalf.
---Response: We have added the suggested information and included it at the end of the cover letter.
--- Before it read: AC was funded by MCIN/AEI/10.13039/501100011033, contract for industrial doctorates aid DIN2021-011964.
--- Now it reads: AC was funded by MCIN/AEI/10.13039/501100011033, contract for industrial doctorates aid DIN2021-011964. The funders had no role in study design, data collection and analysis, decision to publish, or preparation of the manuscript.

3. When completing the data availability statement of the submission form, you indicated that you will make your data available on acceptance. We strongly recommend all authors decide on a data sharing plan before acceptance, as the process can be lengthy and hold up publication timelines. Please note that, though access restrictions are acceptable now, your entire data will need to be made freely accessible if your manuscript is accepted for publication. This policy applies to all data except where public deposition would breach compliance with the protocol approved by your research ethics board. If you are unable to adhere to our open data policy, please kindly revise your statement to explain your reasoning and we will seek the editor's input on an exemption. Please be assured that, once you have provided your new statement, the assessment of your exemption will not hold up the peer review process.
---Response: Perhaps we selected this option in error. All data were already included in the initial submission and were uploaded as supplementary files.
4. We note that Figure 1a in your submission contain map images which may be copyrighted. All PLOS content is published under the Creative Commons Attribution License (CC BY 4.0), which means that the manuscript, images, and Supporting Information files will be freely available online, and any third party is permitted to access, download, copy, distribute, and use these materials in any way, even commercially, with proper attribution. For these reasons, we cannot publish previously copyrighted maps or satellite images created using proprietary data, such as Google software (Google Maps, Street View, and Earth). For more information, see our copyright guidelines: http://journals.plos.org/plosone/s/licenses-and-copyright.
We require you to either present written permission from the copyright holder to publish these figures specifically under the CC BY 4.0 license, or remove the figures from your submission:
a. You may seek permission from the original copyright holder of Figure 1a to publish the content specifically under the CC BY 4.0 license.
We recommend that you contact the original copyright holder with the Content Permission Form (http://journals.plos.org/plosone/s/file?id=7c09/content-permission-form.pdf) and the following text:
“I request permission for the open-access journal PLOS ONE to publish XXX under the Creative Commons Attribution License (CCAL) CC BY 4.0 (http://creativecommons.org/licenses/by/4.0/). Please be aware that this license allows unrestricted use and distribution, even commercially, by third parties. Please reply and provide explicit written permission to publish XXX under a CC BY license and complete the attached form.”
Please upload the completed Content Permission Form or other proof of granted permissions as an "Other" file with your submission.
In the figure caption of the copyrighted figure, please include the following text: “Reprinted from [ref] under a CC BY license, with permission from [name of publisher], original copyright [original copyright year].”
b. If you are unable to obtain permission from the original copyright holder to publish these figures under the CC BY 4.0 license or if the copyright holder’s requirements are incompatible with the CC BY 4.0 license, please either i) remove the figure or ii) supply a replacement figure that complies with the CC BY 4.0 license. Please check copyright information on all replacement figures and update the figure caption with source information. If applicable, please specify in the figure caption text when a figure is similar but not identical to the original image and is therefore for illustrative purposes only.
The following resources for replacing copyrighted map figures may be helpful:
USGS National Map Viewer (public domain): http://viewer.nationalmap.gov/viewer/
The Gateway to Astronaut Photography of Earth (public domain): http://eol.jsc.nasa.gov/sseop/clickmap/
Maps at the CIA (public domain): https://www.cia.gov/library/publications/the-world-factbook/index.html and https://www.cia.gov/library/publications/cia-maps-publications/index.html
NASA Earth Observatory (public domain): http://earthobservatory.nasa.gov/
Landsat: http://landsat.visibleearth.nasa.gov/
USGS EROS (Earth Resources Observatory and Science (EROS) Center) (public domain): http://eros.usgs.gov/#
Natural Earth (public domain): http://www.naturalearthdata.com/
---Response: The data we used to create the map comes from (https://public.opendatasoft.com/explore/dataset/world-administrative-

boundaries/information/) and is licensed under the Open Government Licence v3.0, which is compatible with the Creative Commons Attribution License (CC BY 4.0). We have included this information in the figure caption.
--- Before it read: Time lags and trends in anuran amphibian species descriptions. (a) Cumulative number of frog species described over time. (b) Time lags in the description process. (c) Average description speed per year. Green: description sensu stricto, blue: collection, gray: overall process.
--- Now it reads: Time lags and trends in anuran amphibian species descriptions. (a) Cumulative number of frog species described over time. (b) Time lags in the description process. (c) Average description speed per year. Green: description sensu stricto, blue: collection, gray: overall process. The world map showing country boundaries was obtained from OpenDataSoft (https://public.opendatasoft.com/explore/dataset/world-administrative-boundaries/information/) and is licensed under the Open Government Licence v3.0, which is compatible with the Creative Commons Attribution License (CC BY 4.0).
Additional Editor Comments:
I have now received two reviews for this manuscript. I agree with both reviewers that this manuscript presents an interesting analysis of an impressive dataset, and has some solid results and discussions on a very timely research matter. However, I also share some of reviewer 2's concerns. Some of these are mostly editorial (i.e., the manuscript is quite long and overly detailed in some cases and can be streamlined, as per the excellent suggestion of reviewer 2) and some are related to the content. I agree that some statements made are overly broad and sweeping, and the language can be toned down at times. I also personally have some concerns about advocating fast-track taxonomy and wikification as solutions to the issue. I agree with reviewer 2, also based on my personal experience, that in many cases doing taxonomy properly just takes a whole lot of time, and without investing more resources into there's not much that can be done to speed it up.
---Response: We acknowledge that the manuscript in its original form was quite long and detailed. Following Reviewer #2's suggestions, we have reduced the length of some sections by focusing on the general patterns, which has shortened the manuscript. Additionally, following the editor's and Reviewer #2's suggestions, we have toned down the language throughout the manuscript. Finally, we have placed greater emphasis on the need for increased economic resources for taxonomy.
Overall, I think this is a solid manuscript with a lot of potential, and I encourage the authors to read and adress the reviewer comments carefully if they wish to submit a revised version.
Reviewers' comments:
Reviewer's Responses to Questions
Comments to the Author
1. Is the manuscript technically sound, and do the data support the conclusions?
The manuscript must describe a technically sound piece of scientific research with data that supports the conclusions. Experiments must have been conducted rigorously, with appropriate controls, replication, and sample sizes. The conclusions must be drawn appropriately based on the data presented.
Reviewer #1: Yes
Reviewer #2: Partly
2. Has the statistical analysis been performed appropriately and rigorously?
Reviewer #1: I Don't Know
Reviewer #2: I Don't Know
3. Have the authors made all data underlying the findings in their manuscript fully available?
The PLOS Data policy requires authors to make all data underlying the findings described in their manuscript fully available without restriction, with rare exception (please refer to the Data Availability Statement in the manuscript PDF file). The data should be provided as part of the manuscript or its supporting information, or deposited to a public repository. For example, in addition to summary statistics, the data points behind means, medians and variance measures should be available. If there are restrictions on publicly sharing data—e.g. participant privacy or use of data from a third party—those must be specified.
Reviewer #1: Yes
Reviewer #2: Yes
4. Is the manuscript presented in an intelligible fashion and written in standard English?

PLOS ONE does not copyedit accepted manuscripts, so the language in submitted articles must be clear, correct, and unambiguous. Any typographical or grammatical errors should be corrected at revision, so please note any specific errors here.
Reviewer #1: Yes
Reviewer #2: Yes
5. Review Comments to the Author
Please use the space provided to explain your answers to the questions above. You may also include additional comments for the author, including concerns about dual publication, research ethics, or publication ethics. (Please upload your review as an attachment if it exceeds 20,000 characters)
Reviewer #1: General Assessment:
This manuscript presents a well-conceived and original study based on a remarkable dataset. To the best of my knowledge, it is the first work to quantitatively assess the full duration of the taxonomic description process—from the initial collection of specimens to the eventual publication of species descriptions. The research addresses an important and timely issue in biodiversity science and has the potential to make a significant contribution to the field.
The authors' approach to categorizing and formalizing their dataset is methodologically rigorous, transparent, and well justified. Their assumptions are clearly detailed and appear reasonable and reproducible. However, as I do not have expertise in statistics, I am not in a position to assess the appropriateness or robustness of the statistical tools and models employed in the analysis.
The discussion is pertinent and well articulated. I find the authors' conclusions and recommendations compelling, especially their suggestions for streamlining the taxonomic workflow to enhance the pace of species inventory. That said, I am not certain that there is a clear consensus within the taxonomic community on some of the proposed recommendations, which highlights the value of this work in stimulating a constructive debate around best practices.
Minor Comments:
I would appreciate it if the authors could briefly explain, in the Materials and Methods section, the meaning and relevance of the statistical indices reported in Table 1 (e.g., Skew, MAD, Kurtosis). For readers who are not trained statisticians, a concise clarification of what each index measures and how it contributes to the analysis would improve accessibility.
---Response: We thank the reviewer for their thoughtful and constructive comments, and for their careful reading of our manuscript.
Some of these metrics are already indirectly described in lines 283–291, where we summarize the values obtained for each (or nearly each) metric. However, we agree with Reviewer #1 that a brief explanation of the metrics would improve clarity. Therefore, we have added this information to the Materials and Methods section.
--- Before it read: To summarize and visualize the three periods, we calculated their durations in years for easier interpretation and representation. We obtained the central tendency and position statistics of each period using the base package in R [43], and we calculated asymmetry and dispersion statistics using the psych package [44]. We used the ggplot2 package [45] to represent the results using violin and boxplots. This process was conducted independently for each genus and region, and merging the four regional datasets to obtain global estimates.
--- Now it reads: To summarize and visualize the three periods, we calculated their durations in years for easier interpretation and representation. Using the base package in R [46], we obtained central tendency statistics including the mean, trimmed mean (mean after excluding 10% of extreme values from both ends to reduce the influence of outliers), and median, along with position statistics such as the minimum and maximum. Using the psych package [47], we then calculated measures of asymmetry, including skewness (an asymmetry coefficient where positive values indicate a long right tail, meaning most observations are concentrated below the mean with some high values) and kurtosis (a measure of tail weight and peakedness where high values indicate heavy tails and a sharp central peak compared to a normal distribution). Using the psych package, we also calculated dispersion measures such as the Standard Deviation (S.D.), Median Absolute Deviation (M.A.D.; a robust dispersion measure; calculated as the median of absolute differences from the median, less sensitive to outliers than S.D.), range, and Standard Error (S.E.). We used the ggplot2 package [48] to represent the results using violin and boxplots. This process was conducted independently for each genus and region, followed by merging the four regional datasets to obtain global estimates.

Conclusion:
In summary, this is a well-executed, well-written, and highly informative article. It presents an original contribution to the literature on taxonomic research. I have no major criticisms and only a single minor suggestion for clarification. I recommend this manuscript for publication after this small revision is addressed.
---Response: Thanks.
Reviewer #2:
This paper is an interesting read that provides information on the temporal distribution of key steps on the road to describing frog species in tropical areas. I think with careful revision it could be worth publishing. I have made some more detailed comments on a word version of the MS, but also make some broader comments here.
Tone of writing - Overall I suggest there may be room to be a bit more tempered in some of the claims made. As examples - for vertebrates (and especially terrestrial verts) it is defendable to argue that we do have moderately good framework for understanding patterns in many areas (i.e the existing framework is not a complete disaster). In developing countries, the claim that naming species leads to their protection is contestable at best, as conservation, let alone conservation of small brown frogs, is low on the priority list. In these places in the short-term individual species focused conservation is also not likely to get off the ground – broader region-based approaches are probably more likely to get traction. This is not to say the taxonomy is not important – it totally is and can feed into the above – but it is not the be all and end all to getting conservation happening in tropical countries. Basically - I suggest temper the tone a little in places.
---Response: We thank the reviewer for his thoughtful and constructive comments, as well as for his careful reading of our manuscript. Below, we respond to both the general comments and the specific remarks provided in the annotated version.
We appreciate the suggestion to temper some of our language and recognize that certain statements may have been more strongly worded than intended. We have reviewed the entire manuscript to identify and revise such instances.
Our intent was to highlight that our understanding of diversity and its spatial patterns remains incomplete and will continue like this until we achieve a complete—or at least representative—picture of actual diversity. In amphibians and reptiles, for instance, with over 100 species from each group described annually, taxonomic knowledge is clearly far from complete, reflecting the dynamic and still-developing nature of our understanding. We agree that in some regions, particularly for terrestrial vertebrates, a relatively well-developed framework exists for recognizing broad biodiversity patterns. However, regions such as Melanesia, Madagascar, and other tropical areas—where hundreds of species have been described recently and many more remain undescribed—demonstrate that substantial portions of biodiversity are still undocumented, and that our understanding is still improving and will continue to shift with further discoveries. We have revised the related text to avoid any misinterpretation.
We agree with the reviewer that taxonomy does not guarantee conservation outcomes and that conservation faces challenges unrelated to taxonomic progress. We do not claim that species descriptions directly lead to conservation, but rather that they are a necessary first step—since we cannot conserve what we do not recognize or delimit. This point has been clarified in the text to avoid misinterpretation. We also acknowledge that species-focused conservation is often impractical in regions with limited resources and low political will, which is why our discussion emphasizes the value of region-based approaches considering the undescribed (but known) species. While charismatic species may occasionally act as flagships for conservation, the documentation of multiple well-justified endemics (e.g., brown frogs) can also support habitat protection, aligning with broader goals such as 30 x 30. Ultimately, as noted, conservation decisions depend on socio-political and economic factors beyond taxonomy alone.
Results – I think these can be rationalised a bit. They are quite long at the moment, but many cases are really just verbal summaries of the tables. Try and cut them down. For instance instead of by region summaries – just say the global trends, and note where regions differ from this. The discussion of months where types where collected is not interesting and should be largely or completely cut. There is a high risk that any differences are largely idiosyncratic so I would strongly suggest focusing on the global trends.
---Response: We have substantially reduced the length of certain sections, focusing on global trends as suggested by Reviewer #2.

Powered by Editorial Manager® and ProduXion Manager® from Aries Systems Corporation

--- The section ‘Number of field campaings and temporal patterns of type specimen collection’ has been shortened from 402 to 159 words and now reads:
“Half of the species (476; 53.1%) described between 2000 and 2023 required a single field trip to collect the entire type series, with an average of eight collected specimens per species. Two trips were required for 20.3% of species, three for 11.7%, and four for 6.1%. The remaining ca. 8% were described on the basis of collections made over between five and 23 field trips.
Considering the four regions, species descriptions between 2000 and 2023 were based on type specimens collected between 1893 and 2023, with the majority collected between 1999 and 2016, with some regional differences (S1-S5 Fig).
In Melanesia and Ecuador (S4-S5 Fig), type specimen collection was relatively evenly distributed throughout the year (defined as each month having at least 40% of the number of specimens collected in the peak month). In contrast, Madagascar and India exhibit marked seasonal peaks in collection activity, with certain months showing higher numbers of type specimens collected than others (S2-S3 Fig).”
--- The section ‘Temporal evolution of variables’ has been reduced from 827 to 643 words, following the reviewer’s suggested rationale to focus on the most informative and broadly relevant variables.
Discussion:
Following from the above the discussion is my opinion too long, and includes a lot of general thoughts that are not well connected with the key results and/or quite sweeping. I would suggest this needs a thorough rewrite to make it much more focused.
I would also strongly suggest not framing this around crisis and solution – we have been hearing this for 20 years now (at least) and it has not achieved a great deal. For what it is worth, here is my summary based on working in Melanesia, which likely has over 800 frog species and about 5 active taxonomists (none of which are paid full time to be taxonomists). First there is not going to be a magic bullet, my uni does see describing frogs as a priority activity for me, and neither do most people in Melanesia. That said I do think it is of great value and agree with the authors. So what we can do however is look at the resources we have, the shape the job to be done, and try and prioritise things on criteria such as which things are likely to be most threatened, which descriptions are easiest, and which descriptions will have most impact. If we do this smart we may then be able to attract support to keep working on the job. When people are really keen to work in this space we can support and enable them, while trying to be honest about their job prospects, but we also can’t engineer a trained and employed New Guinea frog taxonomist out of thin air. I am not against new technologies at all, but with 5 people in the space (three which are over 60) we have to be realistic about what we can get done. I would suggest a more focused discussion about how to prioritise resources and potentially to advocate for a few more could be a useful thing that can be done here.
---Response: We appreciate the reviewer’s thoughtful comments and their candid reflection based on first-hand experience in Melanesia. We have taken this feedback seriously and revised the discussion to improve the focus and structure. We acknowledge that the discussion section is relatively long, but we believe it is necessary to properly contextualize our findings and the broader implications of our results.
While some of the discussion topics are not directly derived from our quantitative results, they emerge logically from them, and we believe they are important to address in the context of species description processes.
We did not intend to frame the manuscript strictly as a “crisis and solution” narrative. Rather, we aimed to describe and discuss the current state of species description—including its challenges. We fully agree that there is no “magic bullet,” and we do not suggest otherwise. However, we believe that strategies and pathways for improvement may help address specific bottlenecks and are, therefore, discussed.
Finally, we understand the reviewer’s point about prioritizing species for description. However, in many cases, such prioritization is difficult without first initiating the species delimitation process. Unless a species is highly distinct (e.g., morphologically unique, phylogenetically isolated, or strikingly colored), it is often not possible to anticipate how easily it can be described compared to other undescribed species, its conservation status, the impact of its description, or its broader significance. Therefore, while we agree with the theoretical importance of prioritization, we believe it is not straightforward in practice.
I have noted in the marked up word doc some other cases where sweeping statements

Powered by Editorial Manager® and ProduXion Manager® from Aries Systems Corporation

are good in principle, but in practice can pose issues, or need to be much more fully fleshed out.
---Response: We have carefully reviewed the marked-up Word document and addressed all the noted instances. Below, you will find our detailed responses to each of the points raised.
Areas for consideration with respect to interpretation and analyses
The time from collection to description is actually shorter than I expected. For me this emphasises that we really need to keep collecting (as we and many other have stated in lit). Most new species are relatively newly collected, indicating that the reference libraries of life (museums) have collections that are far from complete. This point has been made, but given increasing hurdles to fieldwork and vouchering, it does not hurt to make it again. If we don't keep collecting - especially in poorly known areas, we are not gunna get the job done.
---Response: We agree that the fact that a substantial proportion of species were collected only a few years prior to their description is significant and worth highlighting. We have therefore revised the relevant paragraph to further emphasize the importance of continued collecting:
--- Before it read: Only 36% of species described since 2000 were collected within a five-year timeframe, suggesting that new species are rarely identified in the field and that targeted sampling campaigns are uncommon. The remaining species were collected earlier —up to 125 years ago— and described much later. This underscores the importance of public collections as reservoirs of potential new species but, at the same time, it concerns about the lack of resources, time, or interest dedicated to examining the collected material.
--- Now it reads: Thirty-six percent of frog species described since 2000 were named within five years of first collection, highlighting the importance of continued collecting. A substantial proportion of recently described species rely on newly collected material, which underscores the need to maintain active fieldwork, particularly in poorly known areas, at a time when logistical, political, and regulatory barriers to collecting and vouchering are increasing. In contrast, the remaining 64% of species were collected earlier —up to 125 years ago— and described much later. This suggests that new species are not always recognized as such in the field, or, if they are, their formal description is not prioritized. It also reflects that targeted sampling aimed at collecting specific, suspected new species is relatively uncommon. They also support the critical role of public scientific collections as reservoirs of undocumented diversity, while also raising concerns about the lack of resources, time, or interest dedicated to examining the collected material.
Is lag time correlated with the number of active taxonomists? I know in Melanesia there are only four living scientists that have described more than five species in this region. I suggest an analysis of how numbers of active authors may influence description speed. This could also illustrate how few people are working on some diverse faunas which would show how tenuous all this knowledge actually is.
---Response: Thank you for this valuable suggestion. In the first submission, we did include the number of active taxonomists as a variable in our analysis as can be seen in the table S2 ‘N_aut_taxon’.
Defining who qualifies as an “active taxonomist” is not straightforward, as (arguably) any author listed on a species description could be considered one.
To address this, we extracted the names of all authors from taxonomic papers published since 2000 and counted the number of times each name appeared across the dataset. We then defined "active taxonomists" as those whose number of appearances exceeded the mean plus the standard deviation. This threshold allowed us to identify highly recurrent contributors. Using this approach, in Melanesia, we identified S. J. Richards, R. Günther, F. Kraus, P. M. Oliver, and A. Allison as active taxonomists—each appearing at least 20 times in species descriptions after the year 2000. This, we think that seems to align well with ‘active taxonomists’ in that region. However, in regions like Ecuador, this threshold excluded some significant but less active (in the last 20 years) taxonomists, whose expertise and impact are nonetheless important.
When we attempted to include the number of active taxonomists per paper as a variable, we found it to be highly correlated with the total number of authors. Due to this strong correlation, we excluded the "number of active taxonomists" from the final model to avoid redundancy, as both variables would have explained essentially the same variation. Instead, we retained the total number of authors to also test the "author inflation" hypothesis.

I would suggest the 11.3 year lag is likely an underestimate going forward, as the sample is biased towards frogs that are being described. The frogs that remain undescribed are increasingly, the hard ones – complexes of confusing or morphologically similar species that are difficult to resolve. One could speculate that lag time may increase as we increasingly get stuck on these species. But only put this in if space opens up – more important in my opinion to rationalise and focus discussion around some of the points raised earlier.
---Response: Thank you for this thoughtful observation. We understand the concern that our estimate of an 11.3-year lag may underestimate future delays, especially as some species pending description are likely part of morphologically cryptic or complex groups (i.e., the difficult ones) that may require more time and advanced tools (e.g., molecular data) to resolve. However, our dataset already includes a number of species described since 2000 that belong to such complexes, suggesting that some of these challenges are already reflected in the current lag time. Moreover, many species in our dataset were collected over 50 and up to 125 years ago, reflecting these 'difficult cases' and counterbalancing the inclusion of more recently collected, 'easier to describe' species. We believe this helps capture a range of descriptive difficulties, although we agree that lag times may increase as taxonomic efforts increasingly focus on the more complex portions of biodiversity.
Keywords
Crisis is misspelt, order words alphabetically
---Response: thanks for the observation, we have made the changes.
6. PLOS authors have the option to publish the peer review history of their article (what does this mean?). If published, this will include your full peer review and any attached files.
If you choose "no", your identity will remain anonymous but your review may still be made public.
Do you want your identity to be public for this peer review? For information about this choice, including consent withdrawal, please see our Privacy Policy.
Reviewer #1: No
Reviewer #2: Yes: Paul Oliver
[NOTE: If reviewer comments were submitted as an attachment file, they will be attached to this email and accessible via the submission site. Please log into your account, locate the manuscript record, and check for the action link "View Attachments". If this link does not appear, there are no attachment files.]
While revising your submission, please upload your figure files to the Preflight Analysis and Conversion Engine (PACE) digital diagnostic tool, https://pacev2.apexcovantage.com/. PACE helps ensure that figures meet PLOS requirements. To use PACE, you must first register as a user. Registration is free. Then, login and navigate to the UPLOAD tab, where you will find detailed instructions on how to use the tool. If you encounter any issues or have any questions when using PACE, please email PLOS at figures@plos.org. Please note that Supporting Information files do not need this step.
Reviewer #2 attached file comments:
L63 True for many groups of inverts, probably not true for plants and vertebrates, suggest clarify
---Response: In this paragraph we are talking about global diversity (in general) for which the statement 'largest proportion of biodiversity is comprised by undescribed species' is completely applicable.
L80 There its now a good ref for barcoding suggesting high levels on unrecognised diversity in Melanesia as well - file:///Users/s2984027/Downloads/biogeography-18-001_article-137988_en_1-4.pdf
---Response: Done. We have added this reference in three different sections of the paper.
L93 Often these steps are not linear like this though, there can often be back and forth while working what units are worthy of the designation of 'species"
---Response: We have partly rephrased to include the reviewers' point.
--- Before it read: Regardless of the chosen approach, describing a species involves five steps, each depending on the previous one —the five D's of taxonomy [36]: discovery, delimitation, diagnosis, description and specimen determination (although not necessarily by the same person). None of these steps can be achieved without previous field work.
--- Now it reads: Regardless of the chosen approach, describing a species involves five steps —the five D's of taxonomy [38]: discovery, delimitation, diagnosis, description

	and specimen determination (although not necessarily by the same person). While these steps are conceptually linear, in practice, the process is often iterative, with researchers revisiting and redefining previous steps as new information emerges or as the species boundaries are reassessed. None of these steps can be achieved without previous field work.<br>L94 Good point - I like this<br>---Response: Thanks.<br>L97 Suggest list some different possible data sources here<br>---Response: We have added some lines of evidence typically used in herpetology.<br>--- Before it read: To decide whether a lineage merits to be named, data from different lines of evidence should ideally be gathered, which requires time and cannot always be achieved within a single field campaign.<br>--- Now it reads: To decide whether a lineage merits to be named, data from different lines of evidence (e.g., morphology, genetics, bioacoustics, ecology) should ideally be gathered, which requires time and cannot always be achieved within a single field trip.<br>L113 We coined a term for this - 'cryptic extinction risk' which may be useful here - https://link.springer.com/article/10.1007/s10531-022-02412-x You could also cite the "dark extinction" papers here<br>---Response: We appreciate the suggestion and have now include the reference. While we recognize the relevance of the term "cryptic extinction risk", we have opted to retain the term "silent extinction", given its broader use.<br>L118 I think reframing these as aims rather than questions reads a bit better<br>---Response: We have followed the reviewer suggestion and changed the goals from questions to aims.<br>L135 And largely in practice..<br>---Response: We appreciate the suggestion. Indeed, we found that only a few taxonomists (sensu lato—that is, authors involved in species descriptions) have participated in species descriptions across more than one country (e.g., R.K. Abraham, A. Fouquet, A. Hamidy, C.R. Hutter, J. Köhler, A. Ohler, and R.A. Pyron), and their involvement in two countries simultaneously is very limited. We have therefore updated the sentence to reflect that the selected regions are taxonomically independent both in principle and largely in practice, as suggested.<br>--- Before it read: These regions are taxonomically independent and, in principle, do not share taxonomists, which helps minimize regional biases in research practices.<br>--- Now it reads: These regions are taxonomically independent and, in principle —and largely in practice— do not share taxonomists, which helps control for regional biases in research practices.<br>L151 There is an ongoing discussion about whether to include the Maluku biota as part of the Melanesian biota. I think either approach is defendable, but you might want to check whether your search terms ending up capturing this region or not<br>---Response: We did not include the Maluku biota because ASW only allows selection of the "South Moluccas" region, which represents only the southern part of the Maluku Islands. Since this filter does not cover the entire Maluku archipelago, we chose to exclude all Maluku Islands from our analysis. Additionally, using the "South Moluccas" filter yields five species, all described before the year 2000. As our analysis focuses exclusively on species described after 2000, applying or omitting this filter would have no effect on the results.<br>L157 This is true, but focusing in on Melanesia, it remains true that a vast majority of descriptions are still based largely on morph and calls, so yes we have new tools, but in very poorly known areas, the older approaches often remain very dominate, reflecting the fact that these biotas are still rife wth obviously different frogs<br>---Response: We agree with Reviewer #2's observation. As our results show, Melanesia currently lags behind in the use of molecular data, with fewer than 40% of species descriptions including genetic data. However, this is likely to change soon, given the recent development of a comprehensive mitochondrial barcoding database for the entire region (Ferreira et al., 2025). Furthermore, we believe that the decision to use molecular data in species descriptions often reflects the author's preference rather than an absolute necessity. Incorporating molecular data does not automatically imply that the species are cryptic relative to their congeners, just as relying solely on morphological traits does not necessarily indicate a larger number of obviously distinct frog species compared to other regions.<br>L195 Authors discretion here - but I feel like field campaign is a bit dramatic - suggest fieldwork and field trip is fine!<br>---Response: We have replaced all instances of "field campaign" with "field trip"

Powered by Editorial Manager® and ProduXion Manager® from Aries Systems Corporation

throughout the manuscript.
L233 This is for the pooled 4 region dataset right, perhaps make that super clear?
---Response: Yes, we have clarified.
--- Before it read: We evaluated the temporal evolution of several variables including the overall description process, the collection, the description sensu stricto…
--- Not it reads: For each region and globally, we evaluated the temporal evolution of several variables including the overall description process, the collection, the description sensu stricto...
L239 Ok this answers my question I think, but perhaps add a clarifying section above as well
---Response: Please, see previous comment.
L241 As written not clear what the percentage is of - percentage of descriptions using genetic evidence?
---Response: We have rephrased.
--- Before it read: In addition, we counted the number of species described each year and the number of species described using genetics to compute the percentage.
--- Not it reads: Additionally, we counted the number of species described each year and the number of those that used genetic data, in order to compute the percentage of species descriptions incorporating genetic evidence per year.
L250 Not a competition - but yay for Melanesia!!! :)
---Response: Haha, yes!
L276 All this is in the table, is possible to just give the verbal summary, and point to the table for the numbers?
---Response: We agree that the essential information is already presented in the table; however, since these represent the main results, we prefer to keep the verbal summary in the text for clarity. That said, we have substantially shortened other sections as suggested by Reviewer #2 to balance the manuscript's length.
L281 I guess Melanesia does not win the competition here!
---Response: Given the points Reviewer #2 raised during this revision, we fully understand that Melanesia does not come out on top in this comparison.
L297 This probably not important, but in a lot of places I feel like one decimal place would be a more meaningful cut-off to use in this paper
---Response: We reviewed the entire manuscript (except the tables and p-values) and reduced the number of decimal places to one for better clarity and consistency.
L303 I will bet a lot go money some of this is an artefact of the fact that this is when universities are not teaching, and lecturers can go into the field - I would be inclined to downplay these monthly patterns somewhat - I think other parts of the paper are more interesting
---Response: Following the recommendation, we have significantly shortened this section (see general Result's comment). While we agree that some of the monthly patterns may be influenced by external factors such as academic calendars, we believe a brief mention still adds useful context regarding seasonal biases in sampling.
L305 These summaries are basically the same as what is in the figures, could consider cutting or further rationilising
---Response: We have significantly reduced the length of this section (see previous comment and general comment).
L317 The main researchers in this region have not had lecturing duties, which may explain the broader spread of collections across the year. Again I don't think this is supper interesting and would suggest cutting.
---Response: We have significantly reduced the length of this section (see previous comment and general comment).
L347 That's an interesting bit of information
---Response: We agree, and we have addressed it in the discussion section.
L350 As above - I found this section to detailed, my strong suggestion would be to go through and rationalise. For instance you could give global trends, but only mention regional trends when they conflict, and otherwise point to the figures
---Response: The section 'Temporal evolution of variables' has been reduced from 827 to 643 words following the reviewer's suggested rationale. However, the evolution of many of these variables is interesting in itself, so it is not straightforward to reduce the length without losing relevant information.
L357 Is this basically saying same thing as first two sentences?
---Response: Yes, we have removed the sentence.
L420 Arguable - EO Wilson was onto this in the 80/90's, Gerald Durrell was writing about it in the 60 and 70's… Could cut these last two sentences, and join with next

	paragraph<br>---Response: We agree with the reviewer that awareness of the extinction crisis began decades ago, as reflected in the work of Wilson, Durrell, and others. Our original use of “generation” was meant to refer to all people currently living on Earth. However, we recognize that this wording may have been ambiguous and have rephrased the passage accordingly.<br>--- Before it read: We are the first generation to fully comprehend that we are in the midst of an extinction event, while the majority of species remain undiscovered and undescribed. We may also be the last generation having the opportunity to explore and document Earth’s biodiversity [37, 51].<br>--- Now it reads: In the past century, we have come to realize that our planet is facing a biodiversity crisis, while the majority of species remain undiscovered and undescribed. Unless we reverse the current trend of threats, we may be among the last generations with the opportunity to explore and document Earth’s biodiversity [39, 54].<br>L444 Don’t follow the logic here, in my experience often the opposite also occurs, things are thought to be new, but careful reveals they are not<br>---Response: We agree that both scenarios can occur and this sentence could be misinterpreted. We have rephrased the paragraph to improve clarity and avoid overgeneralization. Our point is that if 64% of species were described more than five years after collection, this suggests that many new species are either not recognized in the field or are not prioritized for description. In our view, if a species is clearly recognized as new in the field, it should not take ten years or more for its formal description. We acknowledge that delays can occur for many reasons, but taking a decade (or much more) to describe something identified as new from the outset seems excessive, and the less parsimonious explanation. We have rephrased to make it clearer and to include the crucial role of keep collecting.<br>--- Before it read: Only 36% of species described since 2000 were collected within a five-year timeframe, suggesting that new species are rarely identified in the field and that targeted sampling campaigns are uncommon. The remaining species were collected earlier —up to 125 years ago— and described much later. This underscores the importance of public collections as reservoirs of potential new species but, at the same time, it concerns about the lack of resources, time, or interest dedicated to examining the collected material.<br>--- Now it reads: Thirty-six percent of frog species described since 2000 were named within five years of first collection, highlighting the importance of continued collecting. A substantial proportion of recently described species rely on newly collected material, which underscores the need to maintain active fieldwork, particularly in poorly known areas, at a time when logistical, political, and regulatory barriers to collecting and vouchering are increasing. In contrast, the remaining 64% of species were collected earlier —up to 125 years ago— and described much later. This suggests that new species are not always recognized as such in the field, or, if they are, their formal description is not prioritized. It also reflects that targeted sampling aimed at collecting specific, suspected new species is relatively uncommon. They also support the critical role of public scientific collections as reservoirs of undocumented diversity, while also raising concerns about the la...
**Additional Information:**	
**Question**	**Response**
**Financial Disclosure**<br><br>Enter a financial disclosure statement that describes the sources of funding for the work included in this submission. Review the submission guidelines for detailed requirements. View published research articles from *PLOS ONE* for specific examples.<br><br>This statement is required for submission	Yes

and **will appear in the published article** if the submission is accepted. Please make sure it is accurate.<br><br>**Funded studies**<br>Enter a statement with the following details:<br>• Initials of the authors who received each award<br>• Grant numbers awarded to each author<br>• The full name of each funder<br>• URL of each funder website<br>• Did the sponsors or funders play any role in the study design, data collection and analysis, decision to publish, or preparation of the manuscript?<br><br>Did you receive funding for this work?	
Please add funding details.<br>as follow-up to "**Financial Disclosure**<br><br>Enter a financial disclosure statement that describes the sources of funding for the work included in this submission. Review the submission guidelines for detailed requirements. View published research articles from *PLOS ONE* for specific examples.<br><br>This statement is required for submission and **will appear in the published article** if the submission is accepted. Please make sure it is accurate.<br><br>**Funded studies**<br>Enter a statement with the following details:<br>• Initials of the authors who received each award<br>• Grant numbers awarded to each author<br>• The full name of each funder<br>• URL of each funder website<br>• Did the sponsors or funders play any role in the study design, data collection and analysis, decision to publish, or preparation of the manuscript?<br><br>Did you receive funding for this work?"	AC was funded by MCIN/AEI/10.13039/501100011033, contract for industrial doctorates<br>aid DIN2021-011964. The funders had no role in study design, data collection and analysis, decision to publish,<br>or preparation of the manuscript.

Please select the country of your main research funder (please select carefully as in some cases this is used in fee calculation).<br>as follow-up to "**Financial Disclosure**<br><br>Enter a financial disclosure statement that describes the sources of funding for the work included in this submission. Review the submission guidelines for detailed requirements. View published research articles from *PLOS ONE* for specific examples.<br><br>This statement is required for submission and **will appear in the published article** if the submission is accepted. Please make sure it is accurate.<br><br>**Funded studies**<br>Enter a statement with the following details:<br>• Initials of the authors who received each award<br>• Grant numbers awarded to each author<br>• The full name of each funder<br>• URL of each funder website<br>• Did the sponsors or funders play any role in the study design, data collection and analysis, decision to publish, or preparation of the manuscript?<br><br>Did you receive funding for this work?"	SPAIN - ES
**Competing Interests**<br><br>Use the instructions below to enter a competing interest statement for this submission. On behalf of all authors, disclose any competing interests that could be perceived to bias this work—acknowledging all financial support and any other relevant financial or non-financial competing interests.<br><br>This statement is required for submission and **will appear in the published article** if the submission is accepted. Please make sure it is accurate and that any funding	The authors have declared that no competing interests exist.

sources listed in your Funding Information later in the submission form are also declared in your Financial Disclosure statement.<br><br>View published research articles from *PLOS ONE* for specific examples.<br><br>**NO authors have competing interests**<br><br>Enter: *The authors have declared that no competing interests exist.*<br><br>**Authors with competing interests**<br><br>Enter competing interest details beginning with this statement:<br><br>*I have read the journal's policy and the authors of this manuscript have the following competing interests: [insert competing interests here]*<br><br>* typeset	
**Ethics Statement**<br><br>Enter an ethics statement for this submission. This statement is required if the study involved:<br><br>• Human participants<br>• Human specimens or tissue<br>• Vertebrate animals or cephalopods<br>• Vertebrate embryos or tissues<br>• Field research<br><br>Write "N/A" if the submission does not require an ethics statement.<br><br>General guidance is provided below. Consult the submission guidelines for detailed instructions. **Make sure that all information entered here is included in the Methods section of the manuscript.**	This study did not involve any experiments or direct interaction with live animals. All data analyzed were obtained exclusively from previously published literature. Therefore, ethical approval from an Institutional Animal Care and Use Committee (IACUC) or other ethics board was not required for this research.

**Format for specific study types**<br><br>**Human Subject Research (involving human participants and/or tissue)**<br>• Give the name of the institutional review board or ethics committee that approved the study<br>• Include the approval number and/or a statement indicating approval of this research<br>• Indicate the form of consent obtained (written/oral) or the reason that consent was not obtained (e.g. the data were analyzed anonymously)<br><br>**Animal Research (involving vertebrate animals, embryos or tissues)**<br>• Provide the name of the Institutional Animal Care and Use Committee (IACUC) or other relevant ethics board that reviewed the study protocol, and indicate whether they approved this research or granted a formal waiver of ethical approval<br>• Include an approval number if one was obtained<br>• If the study involved *non-human primates*, add *additional details* about animal welfare and steps taken to ameliorate suffering<br>• If anesthesia, euthanasia, or any kind of animal sacrifice is part of the study, include briefly which substances and/or methods were applied<br><br>**Field Research**<br><br>Include the following details if this study involves the collection of plant, animal, or other materials from a natural setting:<br>• Field permit number<br>• Name of the institution or relevant body that granted permission	
**Data Availability**<br><br>Authors are required to make all data underlying the findings described fully available, without restriction, and from the time of publication. PLOS allows rare exceptions to address legal and ethical concerns. See the PLOS Data Policy and FAQ for detailed information.	Yes - all data are fully available without restriction

A Data Availability Statement describing where the data can be found is required at submission. Your answers to this question constitute the Data Availability Statement and **will be published in the article**, if accepted.<br><br>**Important:** Stating 'data available on request from the author' is not sufficient. If your data are only available upon request, select 'No' for the first question and explain your exceptional situation in the text box.<br><br>Do the authors confirm that all data underlying the findings described in their manuscript are fully available without restriction?	
**Describe where the data may be found in full sentences. If you are copying our sample text, replace any instances of XXX with the appropriate details.**<br><br>• If the data are **held or will be held in a public repository**, include URLs, accession numbers or DOIs. If this information will only be available after acceptance, indicate this by ticking the box below. For example: *All XXX files are available from the XXX database (accession number(s) XXX, XXX.*).<br>• If the data are all contained **within the manuscript and/or Supporting Information files**, enter the following: *All relevant data are within the manuscript and its Supporting Information files.*<br>• If neither of these applies but you are able to provide **details of access elsewhere**, with or without limitations, please do so. For example:<br><br>*Data cannot be shared publicly because of [XXX]. Data are available from the XXX Institutional Data Access / Ethics Committee (contact via XXX) for researchers who meet the criteria for access to confidential data.*<br><br>*The data underlying the results presented in the study are available from (include the name of the third party*	All relevant data are within the manuscript and its Supporting Information files.

and contact information or URL).<br>• This text is appropriate if the data are owned by a third party and authors do not have permission to share the data.<br><br>* typeset	
Additional data availability information:	Tick here if the URLs/accession numbers/DOIs will be available only after acceptance of the manuscript for publication so that we can ensure their inclusion before publication.

Powered by Editorial Manager® and ProduXion Manager® from Aries Systems Corporation

Manuscript Click here to access/download;Manuscript;1_Manuscript.docx

Title: Croaking for haste: How long does it take to describe a frog species since its discovery?

Authors: Albert Carné[1, 2, 3*], Alberto Sánchez-Vialas[2], Claudia Lansac[2, 3], Miriam Moreno[5, 6], Ignacio De la Riva[2*]

Corresponding authors: albert.carne@mncn.csic.es (AC), iriva@mncn.csic.es (IDLR)

Short title: Croaking for haste

Affiliations:

[1]Science and Business S.L., Edificio CITEXVI, Vigo, Spain.

[2]Department of Biodiversity and Evolutionary Biology, Museo Nacional de Ciencias Naturales (MNCN), CSIC, Madrid Spain.

[3]Department of Biodiversity Ecology and Evolution, Faculty of Biological Sciences, Complutense University of Madrid, Madrid, Spain.

[4]Biodiversity Research Institute (IMIB), University of Oviedo-Principality of Asturias-CSIC, Mieres, Spain.

[5]Department of Biology of Organisms and Systems, University of Oviedo, Oviedo, Spain.

ORCID IDs:

Albert Carné: https://orcid.org/0000-0002-7920-8159

Claudia Lansac: https://orcid.org/0000-0002-6082-1252

Alberto Sánchez-Vialas: https://orcid.org/0000-0003-0068-7669

Miriam Moreno: https://orcid.org/0009-0009-1568-4795

Ignacio De la Riva: https://orcid.org/0000-0001-5064-4507

Abstract

Global biodiversity faces severe anthropogenic threats, with alarming extinction rates projected for the near future. Most of Earth's diversity remains undescribed, meaning countless species are doomed to extinction before being documented. Since current conservation laws typically consider only described species, the lag time in achieving a representative global biodiversity inventory is a crucial issue impeding effective conservation. Amphibians, the most endangered vertebrate class, exemplify the challenge: while the number of threatened species rises, new species descriptions rapidly increase, and hundreds of candidate species are flagged annually. We analyzed all anuran species described from 2000 to 2023 across four biodiversity-rich tropical regions to investigate the time required to describe new frog species. We quantified the time needed to collect the type series, the number and timing of expeditions, the lag between collection and publication, and the total time between first specimen collection and article publication. Additionally, we explored temporal trends and the effect of selected abiotic variables. On average, it takes 11.3 years to formally describe a frog species since the collection of the first specimen, with 4.5 years spent collecting the type series and 6.8 years on description and publication. These figures were consistent across three of the four regions analyzed. Alarmingly, the time required to describe new species is globally increasing, exacerbating the so-called taxonomic impediment. Thirty-six percent of species were named within five years of first collection, highlighting the need for continued collecting, biological collections as reservoirs of undescribed diversity, and calling for specimen revision after expeditions. These results raise concerns about the effectiveness of current taxonomic and conservation practices addressing the biodiversity crisis. We call for a global effort to prioritize taxonomic research and discuss taxonomic and conservation approaches. Under current practices, and given the observed timelines, we will lose the race against extinction for many species.

Introduction

Global biodiversity is being assaulted by Global Change. Wildlife populations are being reduced, contracted, and extirpated; species are becoming extinct at an unprecedented rate, and entire ecosystems are disappearing before we have the opportunity to properly document them [1–4]. The loss of a species is irreversible and its consequences are unpredictable [5]. The highest rates of decline and extinction are occurring in the tropics, hyper diverse areas where scientific knowledge remains limited, but which are known to contain high levels of undiscovered and undescribed diversity [3, 6–10].

Species are the fundamental unit to measure biodiversity. Only by recognizing the actual species richness of a given region can we reliably understand large-scale ecological and evolutionary trends (e.g., [11]). How many species are on Earth remain an unresolved question —the Linnean shortfall [12–13], partly because of incomplete sampling and the lack of robust biodiversity characterization and extrapolation approaches [14–15]. However, there are three aspects of consensus: first, the largest proportion of biodiversity is comprised by undescribed species [15]; second, the urgency to discover and describe this diversity has never been greater —naming species is a first step towards their conservation, and overcoming the Linnean shortfall is crucial for understanding distributional patterns and, consequently, for reaching effective conservation planning [16–17]; third, we must explore new taxonomic approaches, as the estimated time required to document the remaining biodiversity far exceeds what may be available due to ongoing biodiversity threats [18–19].

Traditional taxonomic approaches, largely based on morphology, face significant challenges in addressing the urgency of the current biodiversity crisis. Integrative taxonomy emerged to accommodate and unify new concepts and methods from various disciplines to ensure the proper description of taxa, taxonomic stability over time, and preventing taxonomic inflation by integrating multiple lines of evidence [18, 20]. A “fast-track” taxonomy proposal arose from the pressing need to characterize and describe as much diversity as possible before it disappears,

significantly shortening the description time by deliberately omitting previously collected material and reducing the most time-consuming parts of the process (i.e., high-quality images vs. detailed and extensive descriptions, reducing descriptions vs. enhancing diagnoses) [21–22]. However, considering the overwhelming number of candidate species awaiting description worldwide (e.g., [10, 23–32], but see [33]), the global richness estimates [15], and the escalating biodiversity crisis, this strategy is likely unable to keep pace with current and projected extinction rates [34], nor to achieve a representative inventory of global species within a reasonable time frame [22]. Some authors, not without receiving criticisms, have attempted the so-called "turbo-taxonomy", which consists in describing large numbers of taxa providing minimal information on each species, sometimes even just a barcoding sequence (e.g., [35]). While this approach may help overcoming the Linnean shortfall, questions arise about the usefulness and reliability of such species "descriptions" [36]. Although "fast-track" and "turbo" taxonomy are sometimes seen as synonyms [36], we distinguish them as two different approaches. We consider as "fast-track" those descriptions that, while concise, include all necessary sections to allow a third party to identify the species without the need for a molecular lab (e.g., [37]), while "turbo" are those minimalist descriptions that do not (e.g., [35]).

Regardless of the chosen approach, describing a species involves five steps —the five D's of taxonomy [38]: discovery, delimitation, diagnosis, description and specimen determination (although not necessarily by the same person). While these steps are conceptually linear, in practice, the process is often iterative, with researchers revisiting and redefining previous steps as new information emerges or as the species boundaries are reassessed. None of these steps can be achieved without previous field work. Fieldwork is time-consuming and, particularly in tropical regions, can be challenging, expensive, and logistically complex. To decide whether a lineage merits to be named, data from different lines of evidence (e.g., morphology, genetics, bioacoustics, ecology) should ideally be gathered, which requires time and cannot always be achieved within a single field trip. Compiling such evidence, preferably including multiple specimens for the type series, can be challenging when cryptic, secretive, seasonal, narrowly-

distributed species, or those with low population densities are involved. This is especially true for frogs, where obtaining call recordings —often a source of key diagnostic characters— can be daunting. Specimens representing populations that clearly merit recognition as distinct species can remain unstudied on shelves for years [39], which negatively impacts their conservation prospects, as undescribed species are more vulnerable to extinction [40].

Amphibians are the most endangered vertebrate class worldwide. More than 41% of described species are under threat, and the number of species at high risk of extinction continues to increase [41]. Threats are ubiquitous throughout the amphibian tree of life, with habitat loss and degradation (primarily due to deforestation), climate change, and emerging diseases being the greatest risks to amphibian persistence [41]. There is a geographical bias, with those regions with greater diversity and lesser available information, i.e., the tropics, being the most affected [3, 42–43]. Paradoxically, current rates of amphibian species description are high (ca. 150 sp. nov./year) and, during the last decade, hundreds of further candidate species have been flagged globally [10, 30–32]. This indicates that we are still far from overcoming the Linnean amphibian shortfall, that the number of threatened species has likely been underestimated, and that many species are facing silent extinction before they are named or even discovered [44].

Here we analyze the type series metadata of all anuran species described since the year 2000 from four tropical regions, to globally and regionally estimate: (1) how long it takes to describe a frog species since the first type specimen is collected using either traditional or contemporary methodologies; (2) how long it takes to get the complete type series for describing a frog species; (3) how long it takes to describe a frog species since all the type material has been collected; (4) how many field trips are needed to describe a frog species; (5) whether description times are decreasing or increasing over time; and (6) the abiotic factors that are related to some frogs being described in a notably shorter time than others, and how these factors have evolved over time.

Materials and methods

This study did not involve any experiments or direct interaction with live animals. All data analyzed were obtained exclusively from previously published literature. Therefore, ethical approval from an Institutional Animal Care and Use Committee (IACUC) or other ethics board was not required for this research.

Region selection, data acquisition and curation

We selected four regions across the globe based on two criteria: high frog species richness (i.e., > 300 species), and an exponential rate of accumulated frog species descriptions. The regions chosen were Ecuador, India, Madagascar, and Melanesia. These regions are taxonomically independent and, in principle —and largely in practice— do not share taxonomists, which helps control for regional biases in research practices. By selecting geographically scattered regions, we aim to investigate the time span of the species description process on a global scale, diluting regional biases to obtain a more accurate timeframe that can be considered globally applicable.

We retrieved the list of described frog species for each region, up to December 2023, from Amphibian Species of the World (ASW; [45]), filtering by higher taxa (Anura) and country, and retaining only native species (i.e., discarding those introduced). Since Melanesia includes two countries —Indonesia and Papua New Guinea—, and parts of Indonesia extend well beyond the region, we could not easily obtain the species list from the online database. Therefore, in accordance with the definition of the Melanesian region provided by Oliver et al. [31], we compiled the species list by selecting “Indonesia-Papua Region”, “Papua New Guinea”, “Solomon Islands”, and “Fiji” (which also includes smaller nearby islands) in ASW.

To determine the species description lags for a contemporary timeframe, we filtered each regional species-list by year, retaining only those species described from the year 2000 (included) onward. We decided to exclude data predating 2000 as software and genetic data —assumed to accelerate species discovery, diagnosis, and description— became widely available and utilized (with regional variability) only in the 21st century. Genetic techniques have become crucial for

describing species, but also for detecting cryptic species and screening regions (and public collections) to unveil overlooked and cryptic diversity (e.g., [10, 30]).

For each species, we retrieved the publication and all collection dates of the type series (holotype and paratypes) from the original descriptions, excluding additional and referred specimens. We only considered unique collection dates; that is, if multiple specimens were collected on the same day, we registered only one date. To deal with imprecise dates (e.g., 5–7 December 2008, February 2007) we used the midpoint date (e.g., 6 December 2008 and 14 February 2007, in the provided examples).

Lag times

We made several necessary assumptions regarding the species description process, and defined three time periods: “overall description process”, “collection”, and “description *sensu stricto*”. We acknowledge that defining these periods and making these assumptions is an oversimplification, but it is necessary to draw conclusions (see Discussion).

We consider the “overall description process” as the time span between the collection date of the first (i.e., the oldest) specimen included in the type series and the publication date of the article introducing the new species to the scientific community. Within this overall process, we differentiate the two other sub-periods, the “collection” and the “description *sensu stricto*”.

The collection period refers to the fieldwork conducted to gather the entire type series, extending from the collection of the first specimen to the last. We assume that all type specimens were considered essential for the species description, which was delayed until the completion of the type series, and that the inclusion of both recently collected and older specimens was not arbitrary. Additionally, we regard the first collected type specimen as the first scientific encounter with the species (i.e., the moment a researcher truly discovers the species), even if it was not immediately recognized as a new taxon in the field and this insight occurred years later.

The description *sensu stricto* is the process encompassing taxon delimitation, diagnosis, description, and determination (*sensu* [38]), spanning from the collection of the last specimen of

the type series to the publication date. At this point, we assumed that once the last specimen of the type series was collected, the description *sensu stricto* started.

To summarize and visualize the three periods, we calculated their durations in years for easier interpretation and representation. Using the *base* package in R [46], we obtained central tendency statistics including the mean, trimmed mean (mean after excluding 10% of extreme values from both ends to reduce the influence of outliers), and median, along with position statistics such as the minimum and maximum. Using the *psych* package [47], we then calculated measures of asymmetry, including skewness (an asymmetry coefficient where positive values indicate a long right tail, meaning most observations are concentrated below the mean with some high values) and kurtosis (a measure of tail weight and peakedness where high values indicate heavy tails and a sharp central peak compared to a normal distribution). Using the *psych* package, we also calculated dispersion measures such as the Standard Deviation (S.D.), Median Absolute Deviation (M.A.D.; a robust dispersion measure; calculated as the median of absolute differences from the median, less sensitive to outliers than S.D.), range, and Standard Error (S.E.). We used the *ggplot2* package [48] to represent the results using violin and boxplots. This process was conducted independently for each genus and region, followed by merging the four regional datasets to obtain global estimates.

Number of field trips and temporal patterns of type specimen collection

To estimate the number of field trips required to collect the entire type series for each species description, we calculated the time span between the sorted collection dates of the specimens in the type series. We considered as distinct field trips those collection events separated by more than one month (i.e., 30 days).

To estimate global and regional temporal patterns in the collection of type specimens (i.e., the number of field trips conducted to collect type specimens each month and year), we calculated

the number of specimens collected per month and year, and represented the collection frequencies using heatmaps with the *ggplot2* package.

Effect of abiotic variables on the description lags

To understand why certain species were described faster than others, we investigated the effect of several abiotic variables on the description *sensu stricto*. We did not analyze the effect of these variables on the overall and collection processes, as the values of these variables are typically determined post-collection.

We included three categorical variables: (1) whether genetics were involved in the species description (YES/NO), (2) the region, and (3) the genus; also, we considered five continuous variables: (1) the number of authors of the description paper, (2) the number of specimens in the type series, (3) the number of species described in each paper, (4) the number of field trips required to collect the type series (see above), and (5) the number of species currently recognized in the genus.

Before fitting the models, we ensured that no numerical variable had a correlation greater than 70% with any other to avoid multicollinearity. For each of the four regions and the global analysis (i.e., combining the four regions into a single dataset), we fitted generalized linear mixed models (GLMM) using the *glmmTMB* R package [49]. In the global model, we included "genus" and "region" as random effects to account for the hierarchical structure of the data. We excluded "region" in the regional models. All models used a Gaussian family with an "identity" link function. We log-transformed the response variable to meet or improve the model assumptions.

To further assess the significance of the relationships, we performed an analysis of variance using the *Anova()* function from the *car* R package [50], which allowed us to evaluate the influence of the predictor variables without considering the factor levels.

We evaluated the model performance using the *DHARMa* package [51], by obtaining the simulated residuals from the model to assess the deviation from the expected distribution, uniformity, outliers, and dispersion. Additionally, we fitted null models (i.e., only including the

random effects) and compared these with the empirical models to determine whether the inclusion of fixed effects in the empirical model, provided a significantly better fit to the data. We conducted this comparison using *anova()* from the *stats* package. Finally, we used the *effects* package [50] to represent the effects of the variables.

Temporal evolution of variables

For each region and globally, we evaluated the temporal evolution of several variables including the overall description process, the collection, the description *sensu stricto*, the number of authors, the number of species described each year, the number of taxonomic papers published annually, the number of species described in each paper, the number of type specimens in each species' type series, the percentage of species described using genetics each year, and the number of field trips undertaken to collect the type series.

To analyze the temporal evolution of these variables, we averaged the yearly values for each region and globally (e.g., the mean time invested in the collection of type specimens for each species described in 2006). Additionally, we counted the number of species described each year and the number of those that used genetic data, in order to compute the percentage of species descriptions incorporating genetic evidence per year.

We used the *ggplot2* package to generate all plots, applying local regressions (LOESS) to fit curves to the data for visualization purposes. To assess the temporal significance of the aforementioned variables, we calculated *p-values* using linear (LM, lm()) or generalized additive models (GAMs, gam()) from the *stats* package with default parameters [46]. We selected the best method based on the comparison of their coefficient of determination (R^2).

Results

Species described since the year 2000 and lag times

Nine hundred and twenty-four frog species have been described across the four analyzed regions between 2000 and 2023. Melanesia leads with 286 newly described frog species, followed by

Madagascar with 215, Ecuador with 213, and India with 210. This represents an average description rate of ca. 10 new species per region each year. After excluding unavailable papers and species with unreported collection or publication dates, we analyzed a total of 2,981 unique collection dates from 896 species; 269 species from Melanesia, 215 from Madagascar, 208 from Ecuador, and 204 from India (S1 Table and S2 Table).

Thirty-six percent of the analyzed species were described within five years of the collection of the first type specimen, and ca. 26% of the species between five and ten years of the first collection. The remaining ca. 40% of the species were described between 10 and 125 years after the first type specimen collection (Fig 1).

Fig 1. Percentage of frog species described since their first collection. Percentage of frog species described as a function of the time elapsed since the collection of the first specimen.

The mean and confidence interval for the global overall description process (i.e., from the collection of the first type-specimen to the paper publication date) is 11.3±0.8 years, with 4.5±0.7 years dedicated to collecting the type series and 6.8±0.5 years focused on the description *sensu stricto* (see Fig 2 and Table 1 for central tendency, position, asymmetry, and dispersion statistics).

Fig 2. Time lags and trends in anuran amphibian species descriptions. (**a**) Cumulative number of frog species described over time. (**b**) Time lags in the description process. (**c**) Average description speed per year. Green: description *sensu stricto*, blue: collection, gray: overall process. The world map showing country boundaries was obtained from OpenDataSoft (https://public.opendatasoft.com/explore/dataset/world-administrative-boundaries/information/) and is licensed under the Open Government Licence v3.0, which is compatible with the Creative Commons Attribution License (CC BY 4.0).

Table 1. Summary statistics for each analyzed region across the different subperiods of the species description process. The table presents the minimum (Min.), mean, standard deviation (S.D.), 10% trimmed mean (Trim. mean), median, median absolute deviation (M.A.D), maximum (Max.), range, skewness (Skew), kurtosis, standard error (S.E.), and 95% confidence interval for the mean (Interval).

	GLOBAL	Ecuador	India	Madagascar	Melanesia
**Species**	896	208	204	215	269
**Overall description process**					
Min.	0.42	0.5	0.42	0.99	0.61
Mean	11.26	11.79	5.77	12.43	14.08

S.D.	12.63	12.58	6.16	10.48	16.18
Trim. mean	8.91	9.43	4.78	11.25	11.01
Median	7.28	6.85	3.87	9.57	8.81
M.A.D	6.59	5.86	3.4	7.84	7.59
Max	125.74	82.95	60.04	110.86	125.74
Range	125.32	82.45	59.62	109.88	125.13
Skew	3.5	2.03	4.08	4.16	3.22
Kurtosis	20.48	5.17	28.75	34.34	15.31
S.E.	0.42	0.87	0.43	0.71	0.99
Interval (95%)	11.26 ± 0.82	11.79 ± 1.71	5.77 ± 0.85	12.43 ± 1.40	14.08 ± 1.93
**Description *sensu stricto***					
Min	0.18	0.33	0.18	0.35	0.57
Mean	6.79	5.54	3.88	6.19	10.45
S.D.	7.52	6.8	3.6	4.46	10.3
Trim. Mean	5.33	4.15	3.27	5.56	8.43
Median	4.41	3.35	2.81	5.3	6.92
M.A.D	3.74	2.83	2.37	3.72	6.33
Max	54.62	51.48	23.44	19.91	54.62
Range	54.45	51.14	23.26	19.56	54.05
Skew	2.85	3.27	2.21	1.21	1.96
Kurtosis	10.57	14.09	6.8	0.82	3.93
S.E.	0.25	0.47	0.25	0.3	0.63
Interval (95%)	6.79 ± 0.49	5.54 ± 0.92	3.88 ± 0.49	6.19 ± 0.60	10.45 ± 1.23
**Collection**					
Min	0	0	0	0	0
Mean	4.47	6.25	1.89	6.24	3.64
S.D.	9.93	10.58	5.13	9.96	11.54
Trim. Mean	2.2	3.92	0.75	4.52	0.92
Median	0.08	1.32	0	3.07	0.01
M.A.D	0.12	1.96	0	4.56	0.02
Max	104.93	79.97	55.84	104.67	104.93
Range	104.93	79.97	55.84	104.67	104.93
Skew	4.94	2.84	6.54	5.05	5.51
Kurtosis	35.46	11.73	59.3	43.02	36.14
S.E.	0.33	0.73	0.36	0.68	0.7
Interval (95%)	4.47 ± 0.65	6.25 ± 1.44	1.89 ± 0.70	6.24 ± 1.33	3.64 ± 1.38

We found significant disparities among regions. India leads in the speed of species descriptions, averaging 5.8±0.9 years of overall process, 1.9±0.7 years for collection, and 3.9±0.5 years for description. Ecuador follows with an overall process time of 11.8±1.7 years, 6.3±1.4 years for collection, and 5.5±0.9 years for description. Madagascar closely trails, with an overall process of 12.4±1.4 years, comprising 6.2±1.3 years for collection and 6.2±0.6 years for description. Finally, the region that takes the longest is Melanesia, with an overall process time of 14.1±1.9 years, including 3.6±1.4 years for collection and 10.5±1.2 years for description (Fig 2 and Table 1).

In all three periods (i.e., overall, collection, and description) and across all five regions (i.e., four independent regions, plus global), we obtained high standard deviation values, with values

exceeding the mean value in 11 out of 15 cases. This indicates a significant dispersion of the data around the mean. The median values consistently fell below the mean, suggesting a skewed distribution. Skewness values were greater than zero in all cases, pointing to positive asymmetry. Kurtosis values consistently exceeded zero, signaling heavy tails and the presence of more extreme values (outliers) that would typically occur in a normal distribution. Additionally, the standard error values remained low in all cases, especially in comparison to the standard deviation, indicating that the obtained mean is a fairly reliable estimate of the population mean.

Number of field trips and temporal patterns of type specimen collection

Half of the species (476; 53.1%) described between 2000 and 2023 required a single field trip to collect the entire type series, with an average of eight collected specimens per species. Two trips were required for 20.3% of species, three for 11.7%, and four for 6.1%. The remaining ca. 8% were described on the basis of collections made over between five and 23 field trips.

Considering the four regions, species descriptions between 2000 and 2023 were based on type specimens collected between 1893 and 2023, with the majority collected between 1999 and 2016, with some regional differences (S1-S5 Fig).

In Melanesia and Ecuador (S4-S5 Fig), type specimen collection was relatively evenly distributed throughout the year (defined as each month having at least 40% of the number of specimens collected in the peak month). In contrast, Madagascar and India exhibit marked seasonal peaks in collection activity, with certain months showing higher numbers of type specimens collected than others (S2-S3 Fig).

Effect of abiotic variables on the description lags

Empiric models differed significantly from the null models and presented lower AIC values, indicating that the models are better than randomness and that the fixed variables have a genuine effect on the data (S3 Table). All models met the model assumptions, except for Ecuador, where

minor violations were observed (S6 Fig). Adding random effects ("genus" in all cases, and both "genus" and "region" in the global analysis) significantly increased the percentage of variance explained by the models (S4 Table).

In all regions except Ecuador, the number of authors had a significant effect on species description time, with an increase in the number of authors associated with a faster description in the global model, Ecuador, and India, and with slower description times in Madagascar and Melanesia. The number of type specimens did not have a significant effect in any region. The number of described species within a genus was marginally significant in Madagascar, where richer genera are associated with faster species descriptions. This variable was not significant in other regions. The number of field trips significantly influenced the description times in the global model and in India, was marginally significant in Ecuador and Madagascar, and showed no significant effect in Melanesia. Across all regions, however, an increase in the number of field trips to collect the type series was associated with shorter description times, perhaps because much of the work had already been done and only complementary data, such as vocalizations, were needed. The number of species described per paper was significant in the global model, Ecuador, and Melanesia, but not in Madagascar or India. An increase in the number of species per paper was associated with longer description times in all regions except in Madagascar, where the effect was the opposite (but not significant). The use of molecular data in species descriptions had a significant effect in the global model and in India but not in Ecuador, Madagascar, or Melanesia. In all regions except Madagascar, incorporating genetic data was associated with longer description times (S7 Fig, S4 Table).

Temporal evolution of variables

Globally, the overall description process decreased from 16 years in 2000 to 7.2 years by 2012. However, after 2012, the description lag increased again, reaching a current value of ca. 11 years (Fig 3). Both collection and description *sensu stricto* followed similar declining trends until 2012, after which the description time rose significantly, while collection time remained stable. Statistical analyses showed that both overall description time and description *sensu stricto* varied

significantly with the year (p-value$_{GAM}$: 0.023 and 0.015, respectively), while collection time remained stable (non-significant).

Fig 3. Temporal evolution of variables in the four analyzed regions. (**a**) Overall description process (black), collection (blue), and description *sensu stricto* (green), (**b**) number of authors in each species description (mean), (**c**) number of published taxonomic papers (count), (**d**) Taxonomic papers that include genetics (percentage), (**e**) number of species described each year (count), (**f**) number of species described in each paper (mean), (**g**) number of type specimens in the type series (mean), (**h**) number of field trips conducted to collect the entire type series (mean).

Ecuador, India, and Melanesia broadly follow the global pattern in the overall description (p-value$_{GAM}$: 0.01, 0.03, and n.s., respectively), in the description *sensu sticto* (p-value$_{GAM}$: 0.01, n.s., and n.s. respectively), and in the collection processes (n.s.). In contrast, Madagascar shows a considerable increase in both the overall description process and description *sensu stricto* (p-value$_{GAM}$: 0.03 and <0.001, respectively), as well as a moderate, non-significant increase in the collection time (S8-S11 Fig).

The number of authors contributing to species descriptions has significantly increased globally (p-value$_{LM}$: <0.001). In Ecuador and India, the number rose from ca. two to five authors, where it appears to have plateaued (p-values$_{GAM}$: <0.001), while in Madagascar and Melanesia, it continues to rise (p-values$_{GAM}$: <0.006) (S8-S11 Fig).

The number of taxonomic papers published globally each year increased from ca. 12 to 23, plateaued until 2020, and then declined (p-value$_{GAM}$: 0.01). Ecuador, India, and Melanesia follow similar patterns (p-values$_{GAM}$: <0.001, <0.001, and n.s.). In contrast, Madagascar shows high variability, with an overall mean of ca. five annual papers, with no significant trend (S8-S11 Fig).

The proportion of taxonomic papers incorporating genetic data has significantly increased globally, reaching 90% by 2022 (p-value$_{GAM}$: <0.001). Madagascar shows the earliest widespread usage, with 100% of papers incorporating genetic data since 2004, with few exceptions (p-value$_{GAM}$: <0.001). In India, the use of genetics remained low (ca. 25%) until 2010, followed by an increase to the current 100% (p-value$_{GAM}$: <0.001). In Ecuador, usage has increased steadily,

currently exceeding 80% (p-value$_{GAM}$: <0.001). In contrast, Melanesia lags behind, with only ca. 40% of papers incorporating genetic data (p-value: 0.005) (S8-S11 Fig).

The number of species described annually increased globally from ca. 25 to 45 until 2010, when it plateaued (p-value$_{GAM}$: 0.01). Ecuador shows a similar trend, reaching a plateau of ca. 12 species per year by 2018 (p-value$_{GAM}$: <0.001). India shows a non-significant steady increase until 2012, followed by a decline. Madagascar and Melanesia show great variability with an overall non-significant continuous increase without reaching a plateau in Madagascar and an overall decrease in Melanesia (S8-S11 Fig).

The average number of species described per paper shows a globally non-significant positive trend. Ecuador and Madagascar show similar patterns, while in India and Melanesia, the number increased until ca. 2013, followed by a decline (S8-S11 Fig).

The number of type specimens in a type series has globally decreased from ca. 20 to fewer than 10 (p-value$_{LM}$ 0.005). A similar pattern is observed in Melanesia (p-value$_{LM}$ 0.003). Ecuador and India show no significant trends. In Madagascar, the overall trend is a steady decrease, although some recently described species have reversed that trend (p-value$_{GAM}$ 0.004) (S8-S11 Fig).

The number of field trips required to collect the entire type series globally shows huge dispersion, with an overall stable trend between 1.5 and 3 field trips required to collect the type series (S8-S11 Fig).

Discussion

Anuran species descriptions amid a biodiversity crisis

Taxonomists have named more than 2.3 million species [52] at a pace of 20,000 species descriptions per year [53]. However, this represents only a small fraction of the world's predicted species richness [15]. In the past century, we have come to realize that our planet is facing a biodiversity crisis, while the majority of species remain undiscovered and undescribed. Unless

we reverse the current trend of threats, we may be among the last generations with the opportunity to explore and document Earth's biodiversity [39, 54].

Here, we evaluated the time required to describe a frog species with a focus on four megadiverse tropical regions. Despite being the most endangered class of vertebrates [41], amphibians are being described at remarkably high rates, and the numerous candidate species flagged worldwide suggest that true amphibian richness —and consequently the number of threatened species— has been, and remains, underestimated [10, 30, 32, 55].

Our results suggest that the mean description time from the collection of the first type specimen to publication is 11.3 years per species, with 4.5 years dedicated to the type series collection and 6.8 years to the description *sensu stricto*. We found similar values across three of the four analyzed regions, with India showing lower values (see Results). Fontaine et al. [39] found similar "shelf" figures for tetrapods analyzing a random set of species described in 2007. Our results indicate that, for frogs, the description times are not decreasing at the global level. While continental regions (e.g., India and Ecuador) exhibit a trend of declining description times, insular regions (e.g., Madagascar and Melanesia) show an increase. This pattern may be explained by the increasing presence and activity of local taxonomists in continental countries, while in the studied insular regions, taxonomy has historically been led by foreign institutions, which are experiencing a reduction in the number of specialists [56]. Overall, our estimate of the global pattern is U-shaped, with current description times being longer than those in 2012 but shorter than those in 2000.

Thirty-six percent of frog species described since 2000 were named within five years of first collection, highlighting the importance of continued collecting. A substantial proportion of recently described species rely on newly collected material, which underscores the need to maintain active fieldwork, particularly in poorly known areas, at a time when logistical, political, and regulatory barriers to collecting and vouchering are increasing. In contrast, the remaining 64% of species were collected earlier —up to 125 years ago— and described much later. This suggests that new species are not always recognized as such in the field, or, if they are, their

formal description is not prioritized. It also reflects that targeted sampling aimed at collecting specific, suspected new species is relatively uncommon. They also support the critical role of public scientific collections as reservoirs of undocumented diversity, while also raising concerns about the lack of resources, time, or interest dedicated to examining the collected material. Public collections contain a wealth of overlooked specimens (e.g., [57–61]). In fact, as Bebber et al. [62] noticed, a significant percentage of the world's undescribed species have already been collected and lodged in museums, but await further scientific investigation and description. In the context of the current biodiversity crisis, some authors have suggested prioritizing the collection and preservation of as many specimens as possible as testimonies of a vanishing biodiversity, ensuring future generations can describe species that may already be extinct [39–63]. Collecting efforts are essential for addressing the geographical and taxonomic gaps that still exist. To maximize their contribution to biodiversity discovery, collecting should be paired with proper specimen processing (including tissue sampling for genetic analysis) and thorough taxonomic revision to promptly detect candidate species and prioritize their description. To describe as many species as possible while they still exist, it is essential to reverse decades of funding cuts to taxonomy. Increased investment would support the creation and maintenance of much-needed taxonomic positions, expand sampling efforts, and enable thorough scrutiny of both recent and historical collections. In parallel, adopting streamlined species description methodologies (e.g., "fast-track taxonomy" in the sense explained above) and integrating molecular data to support rapid identifications can improve efficiency of species discovery and descriptions.

Tropical regions harbor most of Earth's terrestrial biodiversity, yet they remain highly threatened and among the least scientifically understood [64–65]. Large-scale molecular screenings are still relatively rare, but those conducted have revealed high numbers (even hundreds) of potential new species—enabled by comparisons with previously sequenced taxa [10, 30, 32, 55]. Despite growing use of molecular data in species descriptions over the last decades, it has yet to become a standard practice (see results; [53]). Our results suggest that incorporating molecular data into frog descriptions significantly increases description time in some regions. While adding any

procedure to the species descriptions inherently increases the time required, the increase of time can also be attributed to cryptic species, which may remain undescribed on shelves for years until identified molecularly. Sequencing type specimens during species descriptions offers benefits for taxonomy and biodiversity science. DNA-barcodes linked to identified and re-examinable type specimens establish a reliable connection between genetic data and physical vouchers from the outset [66]. This enables unambiguous specimen assignment to newly described species often without taxonomic expertise—except in cases of introgression, hybridization, or human error [67]. Therefore, by leveraging new methodologies and increasingly affordable molecular tools, the widespread use of DNA barcodes to screen collected material should ideally become a standard practice among taxonomists where feasible, especially in poorly surveyed regions [68]. Genetically characterizing the collected material and publicly releasing the georeferenced sequences would not only highlight candidate species—thereby promoting their formal description—but also provide valuable data for other disciplines such as biogeography, evolutionary biology, and ecology. Such efforts could help reduce the Wallacean shortfall [12] by revealing new localities and improving our understanding of intraspecific diversity (e.g., macrogenetics; [69]). Although the ideal species description involves partial sequencing to allow DNA-based identification, genetic comparison, and phylogenetic contextualization, the use of molecular data is not strictly necessary. We acknowledge that socio-economic constraints and legal restrictions on specimen or tissue export in many countries unfortunately limit the widespread implementation of these molecular methods.

Given the number of species still awaiting discovery and description, it is evident that we need more effective solutions for inventory and protect Earth’s amphibian fauna before many species vanish. Over the average 11-year span than takes to formally describe a frog species since the first specimen is collected, vast areas of currently suitable habitats are likely to disappear if current rates of habitat destruction continue [70–71].

Types of species descriptions and future perspectives

A foundational task of taxonomy is comparing the variability of target specimens against a set of related species to assess whether the observed variability justifies considering them as a distinct unit [72]. Early species classifications were constrained by the morphological traits available at the time, leading to minimalist and often rudimentary descriptions due to the limited methodologies and understanding [73–74]. Today, species descriptions are in general more complex, meticulous, and precise. However, significant disparities exist in their current structure and length. Traditional taxonomic practice follows a comprehensive approach to species descriptions, which, over time, incorporated new methodologies and lines of evidence within an integrative framework. This sometimes led to excessively detailed and lengthy descriptions. Although concise species descriptions are not new in taxonomy—Linnaeus, Boulenger, and others provided brief yet sufficient accounts, especially when species diversity was less understood and few cryptic taxa were recognized—what is termed here ‘fast-track taxonomy’ builds on this earlier practice to address the increasingly pressing need to describe species rapidly amid an accelerating biodiversity crisis. This approach does not compromise rigor, since it focuses on essential diagnostic traits, provides concise but accurate descriptions, and incorporates high-quality images to compensate for reduced textual detail. This results in shorter species accounts, ultimately allowing the description of hundreds of species in a single article (e.g., [37–74]). Even more, within the framework of “turbo-taxonomy” [75], some authors describe hundreds of species based only on consensus barcode DNA sequence or molecular diagnostic characters, using these as the principal basis of species delimitation and diagnosis (e.g., [35–76]). However, this approach has been widely criticized, as reliance on mitochondrial DNA or barcodes alone does not provide sufficient evidence for robust species delimitation [77], and it also precludes comparison with already described but unsequenced taxa, increasing the risk of redundant nomenclature.

There is an undeniable urgency to describe as many species as possible before they disappear. However, speeding up the process of naming species should not compromise the quality of species hypotheses [22–36]. Overcoming the Linnean shortfall while ignoring the Wallacean and other

shortfalls (see: [78]) makes little sense if the ultimate goal is to conserve species. Identifying species without understanding where they occur or how they persist in their ecosystems limits the effectiveness of conservation efforts. However, must every shortfall be addressed in the initial process of description? Certain standards are clearly necessary to ensure taxonomic descriptions remain consistent, comparable, and facilitate the work of future taxonomists. Although new methods have emerged to disentangle taxonomic challenges derived from poor descriptions or poorly preserved specimens (e.g., [79]), we still face the consequences of brief historical descriptions, scattered (or lost) type material, and imprecise type localities, which deter anyone from tackling certain taxonomic decisions [21–73].

Although integrative taxonomy has been a good practice of many taxonomists along history, it is in the last decades when taxonomy is truly becoming multidisciplinary, integrating approaches and techniques from disciplines such as genetics, biogeography, bioacoustics, morphometry, and osteology (CT scans). While these approaches may enhance accuracy and provide more information about species, they often require complex techniques and tools that can discourage non-specialists (but see iTaxotools; [80]). But how much complexity is necessary for a species description? Must every bone be described, or all biogeographical affinities revealed upfront?

Another challenge in taxonomy is that much of the original biological materials used in species descriptions are often scattered across museum collections worldwide, making it difficult and expensive to revisit and re-study. Additionally, extensive biodiversity data literature exists, comprising different sets of information. While genetic data are now centralized and easy to retrieve (e.g., GenBank, [81]; BOLD, [82]), other crucial data produced during species descriptions are either absent from accessible repositories, incomplete, or insufficiently standardized, limiting their reuse [53–54]. Even when available, these data may be inaccessible to many due to paywalls, such as those in Zootaxa, one of the most popular taxonomic journals. This hinders taxonomists in resource-limited regions from accessing free descriptions to describe vanishing species in hyperdiverse tropical countries.

A previously suggested strategy to enhance data accessibility and thereby accelerate species descriptions, without compromising species hypothesis is "wikifying" taxonomic data (i.e., cybertaxonomy [83–86]). This approach integrates traditional taxonomic practices with modern digital platforms. The formal description would continue to follow (unless a better solution is found) established taxonomic procedures (i.e., peer-reviewed publications that comply with the ICZN). However, information about a species or candidate species would be available on a centralized digital platform uploaded by ORCID-identified researchers or through referenced literature. This would draw attention to these undescribed lineages and raise awareness among colleagues working on or possessing information about the same taxa [21, 87–88]. If centralized, this system would liberate taxonomic data from conventional publication formats, make it easy to find all information about a species, and facilitate the continuous updating of species descriptions and data before, during, and after their formal description (e.g., DNA sequences, traits, and distributional data). In addition, it could promote the release of data that, due to their magnitude or nature, are not usually published but are nonetheless of interest (e.g., morphological measurements of already described species). This strategy would allow the description of species following a (to be defined) minimum standard to ensure reliable descriptions, with the possibility of later adding information to update them ([21, 53]; and references therein). Consequently, it would not be necessary to delay naming species to provide the most complete description possible. Species could be included in the conservation scheme from the outset, and although the act of naming itself remains static, their descriptions and biological content could remain dynamic and be updated over time. Leveraging the fact that a small number of journals are responsible for the vast majority of published taxonomic papers, these leading journals—as suggested by Miller et al [86]—could take the lead and coordinate with other journals to request standardized taxonomical data (e.g., morphometric values, variable labelling, coordinates) and routinely upload it to digital platforms. Once a shared minimum standard for reliable descriptions and a standardized data submission format is established, other journals and authors could follow the same templates, ensuring consistency and broad adoption across the field. Ultimately, as in many other fields, AI is expected to play an important role in several aspects of taxonomic practice,

most likely contributing to the acceleration of species descriptions. However, discussing the plausible developments of this new tool for taxonomy is beyond the scope of this paper.

Species can be protected through direct conservation efforts by targeting specific species or indirectly by safeguarding their habitats. Undescribed species are not assessed by the IUCN and are generally excluded from biological inventories used to prioritize conservation areas. With the current figures of species descriptions, if we must wait until a species is formally named to protect it, we will lose the race against extinction for many of them. In some cases, taxonomists describe species whose existence has been known (to specialists) for decades, but because formal naming can be delayed by multiple external constraints, when they finally name them, they sadly realize that the locality where it was first collected has been completely destroyed (e.g., [89–90]). This highlights a critical gap in conservation: undescribed species constitute the majority of Earth's biodiversity, but lack formal recognition and, consequently, legal protection, making them more vulnerable to extinction [40].

Therefore, the biggest challenge faced by undescribed species is that current conservation legislation is generally (named)species-centered. Since descriptions are slow and take increasingly longer, many species do not (and will not) have any form of protection unless they happen to occur within a protected area by chance. Using cybertaxonomy could enable including undescribed species in the conservation scheme from the outset and/or enhance a region-centered conservation approach (i.e., indirect conservation). In the latter, we propose accounting for species that have been known for years but remain unnamed—many of which we have substantial data on (e.g., [37]). These species could be used to prioritize conservation areas based on species “richness”, aligning with global targets such as the 30 × 30 initiative, which aims to protect 30% of the planet’s land and oceans by 2030 [91]. This approach would prioritize long-term conservation efforts by considering a more realistic diversity inventory. To achieve this, we suggest publishing all available information on undescribed species, such as DNA barcodes and distribution data, to make it available for regional or global studies. We acknowledge that this approach, in some occasions, could inflate the species inventory of a given area. However, the

worst outcome would be conserving divergent conspecific lineages, which, in any case, represent important genetic variability that should be preserved.

Taxonomy crisis

Taxonomy is a foundational science. Almost all biological studies take place downstream ([38, 54]). Contradictorily, is an undervalued biological discipline [63]. Taxonomic papers receive few citations and are often published in low-impact journals [92–93] threatening the careers of early-career researchers with low citation indices [94–95]. Additionally, funding for taxonomy-focused projects is steadily declining [54, 87, 96–97]. To secure funding and publication in high-impact journals, these projects often need to be framed within cutting-edge methodologies or broader questions. This can result in using a sledgehammer to crack a nut, ultimately extending description times.

Whether taxonomists are facing extinction is hotly debated [56, 63, 93, 98–100]. Some authors argue that the number of authors involved in species descriptions has increased exponentially [56, 101–103]. Others contend that, despite this author inflation, not all authors listed in taxonomic papers can be considered taxonomists [99, 103]. On a global scale, there is a regional turnover, with historically leading taxonomic institutions losing taxonomists, while other regions are increasing their numbers [7, 56]. Our results confirm that the number of authors involved in frog species descriptions has been steadily increasing. However, we found a globally significant relationship between the increase in the number of authors and a reduction in the description time. These results contradict the presumed undesirable effects of author inflation hypothesis, indicating that task distribution among authors may lead to more efficient species descriptions. However, we did not find this relationship in all regions, suggesting that such improved efficiency may depend on the particularities of each research group.

Our results suggest that, since ca. 2010, the number of published frog taxonomical papers, the number of frog species described each year, and the number of species described per paper have remained stable. This contrasts with the growth in scientific productivity, partially driven by

advancements in computational power, new and affordable sequencing technologies, and data analysis software [53, 79–80, 104–106]. Unchanged species diagnosis and description procedures have been flagged as the main cause [21, 53]. Moreover, the number of species described per taxonomist has generally declined. While some authors have attributed this trend to a lack of new species to describe [21, 98, 101, 103], the large number of candidate species flagged annually and current rates of species descriptions suggest otherwise. This decline may reflect a growing shortage of taxonomic specialists and an increased participation in taxonomy of researchers from other fields [99, 103, 107]. This trend may be further exacerbated by the fact that describing a new species, while inherently engaging for most biologists, it is little rewarded. As a result, despite not being taxonomists, some researchers venture into describing one or a few species.

Working in consortiums has been proposed as a strategy to increase the number of species described per article, thus improving the impact metrics of both articles and journals. While this strategy may increase impact metrics, an issue remains: who will be the first author? Is it more advantageous for early-career researchers to be the first author of a less-cited article or to be one among many contributors in a consortium article? Regarding description time, our results suggest that while an increase in the number of authors is associated with shorter description times at a global scale, a higher the number of species described per paper (which is expected to increase the impact metric) has the opposite effect, with more species leading to longer times required to describe each one.

Study caveats

We had to define two subperiods in the species description process and make oversimplified assumptions to analyze data and draw general conclusions. First, the oldest collected specimen in the type series may not represent the species' first scientific encounter, as earlier specimens might have been overlooked (deliberately or not) or stored elsewhere. Even if it was the first encounter, it might not have been recognized as new in the field. In some cases, collectors are not researchers, and the collected material is often not examined immediately after fieldwork. Second, a species can be described using only the holotype (or even part of it). Since not all specimens in the type

series are essential for the species description, the process might not be delayed until their collection. Third, including ancient specimens in the type series of species description was not always strictly necessary, and their inclusion extended the calculated lag time between the first collection and publication. Fourth, the description *sensu stricto* rarely begins immediately after the last type specimen is collected. There are multiple factors that can delay the description of a species for decades. Lastly, we acknowledge that our figures may contain a level of error affecting all species in different degrees, which we did not account for: the time span between manuscript submission and publication. This interval varies depending on the journal and the length of the review process, which can sometimes be lengthy. For the purpose of this study, we have assumed that this interval is part of the species description.

Acknowledgements

We want to acknowledge colleagues who provided fruitful discussions on this issue in the past years, specially Matthijs P. van den Burg and Santiago Castroviejo-Fisher. We are grateful to Ernesto Recuero for his critical reading of the manuscript. We thank the academic editor, Alex Slavenko, as well as Paul Oliver and one anonymous reviewer for their valuable comments and suggestions.

References

1. Lees AC, Pimm SL. Species, extinct before we know them?. Curr Biol. 2015; 25(5): R177–R180.
2. McCallum ML. Vertebrate biodiversity losses point to a sixth mass extinction. Biodiv Cons. 2015; 24(10): 2497–2519.
3. Ceballos G, Ehrlich PR, Dirzo R. Biological annihilation via the ongoing sixth mass extinction signaled by vertebrate population losses and declines. PNAS. 2017; 114(30):E6089-E6096.
4. Ceballos G, Ehrlich PR. The misunderstood sixth mass extinction. Science. 2018; 360(6393): 1080–1081.
5. Young HS, McCauley DJ, Galetti M, Dirzo, R. Patterns, causes, and consequences of anthropocene defaunation. Annu Rev Ecol Evol Syst. 2016; 47: 333–358.
6. Tracewski Ł, Butchart SH, Di Marco M, Ficetola GF, Rondinini C, Symes A, et al. Toward quantification of the impact of 21st-century deforestation on the extinction risk of terrestrial vertebrates. Conserv Biol. 2016; 30(5): 1070–1079.
7. Prathapan KD, Rajan PD. Advancing taxonomy in the global south and completing the grand Linnaean enterprise. Megataxa. 2020; 1(1): 73–77.
8. Button S, Borzée A. A new multi-metric approach for quantifying global biodiscovery and conservation priorities reveals overlooked hotspots for amphibians. [Preprint]. arXiv:2308.08829. 2023 [cited 2024 Dec 12]. Available from: https://arxiv.org/abs/2308.08829.
9. Finn C, Grattarola F, Pincheira-Donoso D. More losers than winners: investigating Anthropocene defaunation through the diversity of population trends. Biol Rev. 2023; 98(5):1732–1748.

10. Carné A, Vieites DR. A race against extinction: The challenge to overcome the Linnean amphibian shortfall in tropical biodiversity hotspots. Divers Distrib. 2024; 30(12): 1–13.
11. Burriel-Carranza B, Tejero-Cicuéndez H, Carné A, Mochales-Riaño G, Talavera A, Al Saadi S, et al. Integrating genomics and biogeography to unravel the origin of a mountain biota: The case of a reptile endemicity hotspot in Arabia. Syst Biol. 2024; syae032.
12. Lomolino MV. Conservation biogeography. In: Lomolino MV, Heaney LR, editors. Frontiers of biogeography: New directions in the geography of nature. Sunderland (MA): Sinauer; 2004. p. 293–296.
13. Brown JH, Lomolino MV. Biogeography. 2nd ed. Sunderland (MA): Sinauer Press; 1998.
14. Mora C, Tittensor DP, Adl S, Simpson AG, Worm B. How many species are there on Earth and in the ocean? PLoS Biol. 2011; 9(8): e1001127.
15. Larsen BB, Miller EC, Rhodes MK, Wiens JJ. Inordinate fondness multiplied and redistributed: the number of species on earth and the new pie of life. Q Rev Biol. 2017; 92(3): 229–265.
16. Mace GM. The role of taxonomy in species conservation. Phil Trans R Soc B. 2004; 359(1444): 711–719.
17. Nori J, Semhan R, Abdala CS, Rojas-Soto O. Filling Linnean shortfalls increases endemicity patterns: conservation and biogeographical implications for the extreme case of *Liolaemus* (Liolaemidae, Squamata) species. Zool J Linn Soc. 2022; 194(2): 592–600.
18. Padial JM, Miralles A, De la Riva I, Vences M. The integrative future of taxonomy. Front Zool. 2010; 7(1): 1–14.
19. Scheffers BR, Joppa LN, Pimm SL, Laurance WF. What we know and don't know about Earth's missing biodiversity. Trends Ecol Evol. 2012; 27: 501–510.
20. Padial JM, Castroviejo-Fisher S, Koehler J, Vila C, Chaparro JC, De la Riva I. Deciphering the products of evolution at the species level: the need for an integrative taxonomy. Zool Scr. 2009; 38(4): 431–447.
21. Riedel A, Sagata K, Suhardjono YR, Tänzler R, Balke M. Integrative taxonomy on the fast track-towards more sustainability in biodiversity research. Front Zool. 2013; 10: 1–9.
22. Vences M. The promise of next-generation taxonomy. Megataxa. 2020; 1(1): 35–38.
23. Meegaskumbura M, Bossuyt F, Pethiyagoda R, Manamendra-Arachchi K, Bahir M, Milinkovitch MC, Schneider CJ. Sri Lanka: an amphibian hot spot. Science. 2002; 298(5592): 379–379.
24. Fouquet A, Gilles A, Vences M, Marty C, Blanc M, Gemmell NJ. Underestimation of species richness in Neotropical frogs revealed by mtDNA analyses. PLoS ONE. 2007; 2(10):e1109.
25. Clare EL, Lim BK, Fenton MB, Hebert PD. Neotropical bats: estimating species diversity with DNA barcodes. PLoS ONE. 2011; 6(7): e22648.
26. Funk WC, Caminer M, Ron SR. High levels of cryptic species diversity uncovered in Amazonian frogs. Proc R Soc B. 2012; 279(1734): 1806–1814.
27. Milá B, Tavares ES, Munoz Saldana A, Karubian J, Smith TB, Baker AJ. A trans-Amazonian screening of mtDNA reveals deep intraspecific divergence in forest birds and suggests a vast underestimation of species diversity. PLoS ONE. 2012; 7(7): e40541.
28. Tänzler R, Sagata K, Surbakti S, Balke M, Riedel A. DNA barcoding for community ecology-how to tackle a hyperdiverse, mostly undescribed Melanesian fauna. PLoS ONE. 2012; 7(1): e28832.
29. Veijalainen A, Wahlberg N, Broad GR, Erwin TL, Longino JT, Sääksjärvi IE. Unprecedented ichneumonid parasitoid wasp diversity in tropical forests. Proc R Soc Ser B Biol. Sci. 2012; 279(1748): 4694–4698.
30. Vacher JP, Chave J, Ficetola FG, Sommeria-Klein G, Tao S, Thébaud C, et al. Large-scale DNA-based survey of frogs in Amazonia suggests a vast underestimation of species richness and endemism. J Biogeogr. 2020; 47(8): 1781–1791.
31. Oliver PM, Bower DS, McDonald PJ, Kraus F, Luedtke J, Neam K, et al. Melanesia holds the world's most diverse and intact insular amphibian fauna. Comm Biol. 2022; 5(1): 1182.
32. Ferreira F, Oliver P, Kraus F, Günther R, Richards S, Tjaturadi B, et al. Molecular and acoustic evidence for large-scale underestimation of frog species diversity on New Guinea. Front Biogeogr. 2025;18:e137988.
33. Chan KO, Hutter CR, Wood Jr PL, Su YC, Brown RM. (2022). Gene flow increases phylogenetic structure and inflates cryptic species estimations: a case study on widespread Philippine puddle frogs (*Occidozyga laevis*). Syst. Biol. 2022; 71(1): 40-57.
34. Cowie RH, Bouchet P, Fontaine B. The Sixth Mass Extinction: fact, fiction or speculation?. Biol Rev. 2022; 97(2): 640–663.
35. Sharkey MJ, Janzen DH, Hallwachs W, Chapman EG, Smith MA, Dapkey T, et al. Minimalist revision and description of 403 new species in 11 subfamilies of Costa Rican braconid parasitoid wasps, including host records for 219 species. ZooKeys. 2021; 1013:1–665.

36. Fernandez-Triana JL. Turbo taxonomy approaches: lessons from the past and recommendations for the future based on the experience with Braconidae (Hymenoptera) parasitoid wasps. ZooKeys. 2022; 1087: 199-220.
37. Rakotoarison A, Scherz M, Glaw F, Köhler J, Andreone F, Franzen M, et al. Describing the smaller majority: integrative taxonomy reveals twenty-six new species of tiny microhylid frogs (genus *Stumpffia*) from Madagascar. Vertebr Zool. 2017; 67: 271–398.
38. Favret C. The 5 'D's of Taxonomy: A User's Guide. Q Rev Biol. 2024; 99(3): 131–156.
39. Fontaine B, Perrard A, Bouchet P. 21 years of shelf life between discovery and description of new species. Curr Biol. 2012; 22(22): R943–R944.
40. Liu J, Slik F, Zheng S, Lindenmayer DB. Undescribed species have higher extinction risk than known species. Conserv Lett. 2022; 15(3): e12876.
41. Luedtke JA, Chanson J, Neam K, Hobin L, Maciel AO, Catenazzi A, et al. Ongoing declines for the world's amphibians in the face of emerging threats. Nature. 2023; 622(7982): 308–314.
42. Stuart SN, Chanson JS, Cox NA, Young BE, Rodrigues AS, Fischman DL, et al. Status and trends of amphibian declines and extinctions worldwide. Science. 2004; 306: 1783–1786.
43. Stuart SN, Hoffmann M, Chanson JS, Cox NA, Berridge RJ, Ramani PP, Young BE, editors. Threatened Amphibians of the World. Barcelona, Spain: Lynx Edicions; Gland, Switzerland: IUCN; Arlington, Virginia, USA: Conservation International; 2008.
44. McDonald PJ, Brown RM, Kraus F, Bowles P, Arifin U, Eliades SJ, et al. Cryptic extinction risk in a western Pacific lizard radiation. Biodivers Conserv. 2022; 31(8): 2045–2062.
45. Frost DR. Amphibian Species of the World: An Online Reference. Version 6.2 [Internet]. 2024 [cited 2024 Dec 12]. Available from: https://amphibiansoftheworld.amnh.org/index.php
46. R Core Team. R: A language and environment for statistical computing. R Foundation for Statistical Computing; 2024. Available from: https://www.R-project.org/.
47. Revelle W. psych: Procedures for Psychological, Psychometric, and Personality Research. R package version 2.4.6. Northwestern University, Evanston, Illinois; 2024. Available from: https://CRAN.R-project.org/package=psych.
48. Wickham H. ggplot2: Elegant Graphics for Data Analysis. Springer-Verlag New York; 2016.
49. Brooks ME, Kristensen K, van Benthem KJ, Magnusson A, Berg CW, Nielsen A, et al. glmmTMB balances speed and flexibility among packages for zero-inflated generalized linear mixed modeling. The R Journal. 2017; 9(2): 378–400.
50. Fox J, Weisberg S. An R companion to applied regression. 3rd ed. Thousan Oaks (CA):Sage; 2019.
51. Hartig F. DHARMa: Residual Diagnostics for Hierarchical (Multi-Level/Mixed) Regression Models [Internet]. R package version 0.4.7. 2024 [cited 2024 Dec 12]. Available from: https://CRAN.R-project.org/package=DHARMa.
52. Bánki O, Roskov Y, Döring M, Ower G, Hernández Robles DR, Plata Corredor CA, et al. Catalogue of Life [Internet]. Version 2024-07-18. Amsterdam (Netherlands): Catalogue of Life; 2024 [cited 2024 Dec 12]. Available from: https://doi.org/10.48580/dgbqz.
53. Miralles A, Bruy T, Wolcott K, Scherz MD, Begerow D, Beszteri B, et al. Repositories for taxonomic data: where we are and what is missing. Syst Biol. 2020; 69(6): 1231–1253.
54. Wheeler QD, Raven PH, Wilson EO. Taxonomy: impediment or expedient?. Science. 2004; 303(5656): 285–285.
55. Jaramillo AF, De la Riva I, Guayasamin JM, Chaparro JC, Gagliardi Urrutia G, Gutiérrez R, et al. Vastly underestimated radiation of Amazonian salamanders (Plethodontidae: Bolitoglossa) and implications about plethodontid diversification. Mol Phylogenet Evol. 2020; 149: 1–23.
56. Costello MJ, May RM, Stork NE. Can we name Earth's species before they go extinct? Science. 2013; 339(6118): 413–416.
57. Sánchez-Vialas A, Calvo-Revuelta M, Castroviejo-Fisher S, De la Riva I. Synopsis of the amphibians of Equatorial Guinea based upon the authors' field work and Spanish natural history collections. Proc Calif Acad Sci. 2020; 66:137–230.
58. Sánchez-Vialas A, Calvo-Revuelta M, De la Riva I. Synopsis of the reptiles of Equatorial Guinea. Zootaxa. 2022; 5202(1):1–197.
59. Sánchez-Vialas A, Copete-Mosquera LA, Calvo-Revuelta M. Contributions to the amphibians and reptiles of Myanmar: insights from the Leonardo Fea legacy housed at the Museo Nacional de Ciencias Naturales of Madrid. Zootaxa. 2024, 5457(1): 1–64.
60. Brown RM, Calvo-Revuelta M, Goyes Vallejos J, Sánchez-Vialas A, De la Riva I. On the second (or, rather, the first) specimen of the recently described *Calliophis salitan* (Squamata: Elapidae), with the first report of the species from Mindanao Island, southern Philippines. Herpetology notes. 2021; 14: 1027–1035.

61. López-Estrada EK, Sánchez-Vialas A, Manzanilla J, Piñango C, Ruiz JL, García-París M. An overview of the taxonomy and geographic distribution of Venezuelan *Epicauta* (Coleoptera: Meloidae). Ann Zool. 2022; 72(1): 9–47.
62. Bebber DP, Carine MA, Wood JR, Wortley AH, Harris DJ, Prance GT, et al. Herbaria are a major frontier for species discovery. PNAS. 2010; 107(51): 22169–22171.
63. Engel MS, Ceríaco LM, Daniel GM, Dellapé PM, Löbl I, Marinov M, et al. The taxonomic impediment: a shortage of taxonomists, not the lack of technical approaches. Zool J Linn Soc. 2021; 193(2): 381–387.
64. Moura MR, Jetz W. Shortfalls and opportunities in terrestrial vertebrate species discovery. Nat Ecol Evol. 2021; 5(5):631–9.
65. Šmíd J. Geographic and taxonomic biases in the vertebrate tree of life. J Biogeogr. 2022;49(12):2120–9.
66. Buckner JC, Sanders RC, Faircloth BC, Chakrabarty P. The critical importance of vouchers in genomics. Elife. 2021;10:e68264.
67. Carné A, Vieites DR, van den Burg MP. In Vouchers We (Hope to) Trust: Unveiling Hidden Errors in GenBank's Tetrapod Taxonomic Foundations. Mol Ecol. 2025; e17812.
68. Padial JM, De la Riva I. Integrative taxonomists should use and produce DNA barcodes. Zootaxa. 2007; 1586: 67–68.
69. Blanchet S, Prunier JG, De Kort H. Time to go bigger: emerging patterns in macrogenetics. Trends Genet. 2017; 33(9): 579–580.
70. Vieilledent G, Grinand C, Rakotomalala FA, Ranaivosoa R, Rakotoarijaona JR, Allnutt TF, et al. Combining global tree cover loss data with historical national forest cover maps to look at six decades of deforestation and forest fragmentation in Madagascar. Biol Conserv. 2018; 222: 189–197.
71. Silva Junior CH, Pessôa AC, Carvalho NS, Reis JB, Anderson LO, Aragão LE. The Brazilian Amazon deforestation rate in 2020 is the greatest of the decade. Nat Ecol Evol. 2021; 5(2): 144–145.
72. Mayr E. Principles of systematic zoology. McGraw-Hill; 1969.
73. Godfray HCJ. Challenges for taxonomy. Nature. 2002; 417(6884): 17–19.
74. Riedel A, Narakusumo RP. One hundred and three new species of *Trigonopterus* weevils from Sulawesi. ZooKeys. 2019; 828: 1–153.
75. Butcher BA, Smith MA, Sharkey MJ, Quicke DL. A turbo-taxonomic study of Thai *Aleiodes* (*Aleiodes*) and *Aleiodes* (*Arcaleiodes*) (Hymenoptera: Braconidae: Rogadinae) based largely on COI barcoded specimens, with rapid descriptions of 179 new species. Zootaxa. 2012; 3457(1): 1–232.
76. Meierotto S, Sharkey MJ, Janzen DH, Hallwachs W, Hebert PD, Chapman EG, et al. A revolutionary protocol to describe understudied hyperdiverse taxa and overcome the taxonomic impediment. Dtsch Entomol. 2019; 66(2): 119–145.
77. Reyes-Velasco J. A revision of recent taxonomic changes to the eyelash palm pitviper, *Bothriechis schlegelii* (Serpentes, Viperidae). Herpetozoa. 2024; 37: 305–318.
78. Hortal J, de Bello F, Diniz-Filho JAF, Lewinsohn TM, Lobo JM, Ladle RJ. Seven shortfalls that beset large-scale knowledge of biodiversity. Annu Rev Ecol Evol. Syst. 2015; 46(1): 523–549.
79. Rancilhac L, Bruy T, Scherz MD, Pereira EA, Preick M, Straube N, et al. Target-enriched DNA sequencing from historical type material enables a partial revision of the Madagascar giant stream frogs (genus *Mantidactylus*). J Nat Hist. 2020; 54(1–4): 87–118.
80. Vences M, Miralles A, Brouillet S, Ducasse J, Fedosov A, Kharchev V, et al. iTaxoTools 0.1: Kickstarting a specimen-based software toolkit for taxonomists. Megataxa. 2021; 6:77–92.
81. Benson DA, Karsch-Mizrachi I, Lipman DJ, Ostell J, Sayers EW. GenBank. Nucleic Acids Res. 2010; 38: 46–51.
82. Ratnasingham S, Hebert PD. BOLD: The Barcode of Life Data System (http://www. barcodinglife. org). Mol Ecol Notes. 2007; 7(3): 355–364.
83. Godfray HC.J, Clark BR, Kitching IJ, Mayo SJ, Scoble MJ. The web and the structure of taxonomy. Syst. Biol. 2007; 56(6): 943–955.
84. Mietchen D, Hagedorn G, Förstner KU, Kubke MF, Koltzenburg C, Hahnel M, Penev L. Wikis in scholarly publishing. Information Services and Use. 2011; 31(1-2): 53–59.
85. Maddison DR, Guralnick R, Hill A, Reysenbach AL, McDade LA. Ramping up biodiversity discovery via online quantum contributions. Trends Ecol Evol. 2012; 27(2): 72–77.
86. Miller J, Dikow T, Agosti D, Sautter G, Catapano T, Penev L, et al.. From taxonomic literature to cybertaxonomic content. BMC biology. 2012; 10(1): 87.
87. Godfray Jr HCJ. Linnaeus in the information age. Nature. 2007; 446(7133): 259–260.
88. Wheeler QD, Knapp S, Stevenson DW, Stevenson J, Blum SD, Boom BM, et al. Mapping the biosphere: exploring species to understand the origin, organization and sustainability of biodiversity. Syst Biodivers. 2012; 10(1): 1–20.

89. Barratt CD, Lawson LP, Bittencourt-Silva GB, Doggart N, Morgan-Brown T, Nagel, P, et al. A new, narrowly distributed, and critically endangered species of spiny-throated reed frog (Anura: Hyperoliidae) from a highly threatened coastal forest reserve in Tanzania. Herpetol J. 2017; 27(1):13-24.
90. Vences M, Köhler J, Scherz MD, Hutter CR, Maheritafika HMR, Rafanoharana JM, et al. Four new species of forest-dwelling mantellid frogs from Madagascar allied to *Gephyromantis moseri*. Spixiana. 2024; 46(2):297-319.
91. Dinerstein E, Vynne C, Sala E, Joshi AR, Fernando S, Lovejoy TE, et al. A global deal for nature: Guiding principles, milestones, and targets. Sci Adv. 2019; 5(4): eaaw2869.
92. Agnarsson I, Kuntner M. Taxonomy in a changing world: seeking solutions for a science in crisis. Syst Biol. 2007; 56(3): 531–539.
93. Drew LW. Are We Losing the Science of Taxonomy?. BioScience. 2011; 61(12): 942–946.
94. Krell FT. Why impact factors don't work for taxonomy. Nature. 2002; 415(6875): 957–957.
95. Walter DE, Winterton S. Keys and the crisis in taxonomy: extinction or reinvention?. Annu Rev Entomol. 2007; 52(1): 193–208.
96. Britz R, Hundsdörfer A, Fritz U. Funding, training, permits—the three big challenges of taxonomy. Megataxa. 2020; 1(1): 49–52.
97. Löbl I, Klausnitzer B, Hartmann M, Krell FT. The silent extinction of species and taxonomists—An appeal to science policymakers and legislators. Diversity. 2023; 15(10):1053.
98. Tancoigne E, Dubois A. Taxonomy: no decline, but inertia. Cladistics. 2013; 29(5): 567–570.
99. De Carvalho MR, Ebach MC, Williams DM, Nihei SS, Trefaut Rodrigues M, Grant T, et al. Does counting species count as taxonomy? On misrepresenting systematics, yet again. Cladistics. 2014; 30(3): 322–329.
100. Bradford-Grieve J. Is there a taxonomic crisis?. New Zealand Science Review. 2016; 73(3–4): 83–86.
101. Joppa LN, Roberts DL, Pimm SL. The population ecology and social behaviour of taxonomists. Trends Ecol Evol. 2011; 26(11): 551–553.
102. Bacher S. Still not enough taxonomists: reply to Joppa et al. Trends Ecol Evol. 2012; 27(2): 65–66.
103. Bebber DP, Wood JR, Barker C, Scotland RW. Author inflation masks global capacity for species discovery in flowering plants. New Phytol. 2014; 201(2):700–706.
104. Wetterstrand K. DNA sequencing costs [Internet]. Available from: www.genome.gov/sequencingcostsdata. [cited 2024 Nov 26].
105. Puillandre N, Brouillet S, Achaz G. ASAP: assemble species by automatic partitioning. Mol Ecol Resour. 2021; 21(2): 609–620.
106. Ramírez JL, Valdivia P, Rosas-Puchuri U, Valdivia NL. SPdel: A pipeline to compare and visualize species delimitation methods for single-locus datasets. Mol Ecol Resour. 2023; 23(8): 1959–1965.
107. Tancoigne E, Ollivier G. Evaluating the progress and needs of taxonomy since the Convention on Biological Diversity: going beyond the rate of species description. Aust Syst Bot. 2017; 30(4): 326–336.

Supporting information

S1 Fig. Heatmap of global specimen collections. Heatmap showing the distribution of specimen collection events in the four analyzed regions over time. The x-axis represents the months of the year, while the y-axis represents the years of collection. The color intensity indicates the number of specimens collected in each time period.

S2 Fig. Heatmap of specimen collections in Madagascar. Heatmap showing the distribution of specimen collection events in Madagascar over time. The x-axis represents the months of the year, while the y-axis represents the years of collection. The color intensity indicates the number of specimens collected in each time period.

S3 Fig. Heatmap of specimen collections in India. Heatmap showing the distribution of specimen collection events in India over time. The x-axis represents the months of the year, while the y-axis represents the years of collection. The color intensity indicates the number of specimens collected in each time period.

S4 Fig. Heatmap of specimen collections in Melanesia. Heatmap showing the distribution of specimen collection events in Melanesia over time. The x-axis represents the months of the year, while the y-axis represents the years of collection. The color intensity indicates the number of specimens collected in each time period.

S5 Fig. Heatmap of specimen collections in Ecuador. Heatmap showing the distribution of specimen collection events in Ecuador over time. The x-axis represents the months of the year, while the y-axis represents the years of collection. The color intensity indicates the number of specimens collected in each time period.

S6 Fig. Residual diagnostics from the DHARMa R package for the fitted models.

S7 Fig. Significant effect plots of variables on description *sensu stricto*. Colored boxes differentiate the regions. Gray= global, Purple = Ecuador, Orange= Madagascar, Green = India, and Blue = Melanesia.

S8 Fig. Temporal evolution of variables in Ecuador. (**a**) Overall description process (black), collection (blue), and description *sensu stricto* (green), (**b**) Nº of authors in each species description (mean), (**c**) Number of published taxonomic papers (count), (**d**) Taxonomic papers that include genetics (percentage), (**e**) Nº of species described each year (count), (**f**) Nº of species described in each paper (mean), (**g**) Nº of type specimens in the type series (mean), (**h**) Nº of field trips conducted to collect the entire type series (mean).

S9 Fig. Temporal evolution of variables in India. (**a**) Overall description process (black), collection (blue), and description *sensu stricto* (green), (**b**) Nº of authors in each species description (mean), (**c**) Number of published taxonomic papers (count), (**d**) Taxonomic papers that include genetics (percentage), (**e**) Nº of species described each year (count), (**f**) Nº of species described in each paper (mean), (**g**) Nº of type specimens in the type series (mean), (**h**) Nº of field trips conducted to collect the entire type series (mean).

S10 Fig. Temporal evolution of variables in Madagascar. (**a**) Overall description process (black), collection (blue), and description *sensu stricto* (green), (**b**) Nº of authors in each species description (mean), (**c**) Number of published taxonomic papers (count), (**d**) Taxonomic papers that include genetics (percentage), (**e**) Nº of species described each year (count), (**f**) Nº of species described in each paper (mean), (**g**) Nº of type specimens in the type series (mean), (**h**) Nº of field trips conducted to collect the entire type series (mean).

S11 Fig. Temporal evolution of variables in Melanesia. (**a**) Overall description process (black), collection (blue), and description *sensu stricto* (green), (**b**) Nº of authors in each species description (mean), (**c**) Number of published taxonomic papers (count), (**d**) Taxonomic papers that include genetics (percentage), (**e**) Nº of species described each year (count), (**f**) Nº of species described in each paper (mean), (**g**) Nº of type specimens in the type series (mean), (**h**) Nº of field trips conducted to collect the entire type series (mean).

S1 Table. Collection and publication dates for each species and region extracted from the literature.

S2 Table. Lag times of collection (MEX_MIN_YEAR_COLPUB), description *sensu stricto* (MIN_YEAR_COLPUB) and overall process (MAX_YEAR_COLPUB) for each species and region. Categorical and numerical variables used in models are also included.

S3 Table. Statistical comparison of AIC values between empiric (fixed + random variables) and null (random variables only) models.

S4 Table. p-values from the GLMM models and evaluation metrics for global and regional analyses.

Figure_1

Click here to access/download;Figure;2_Fig_1.eps

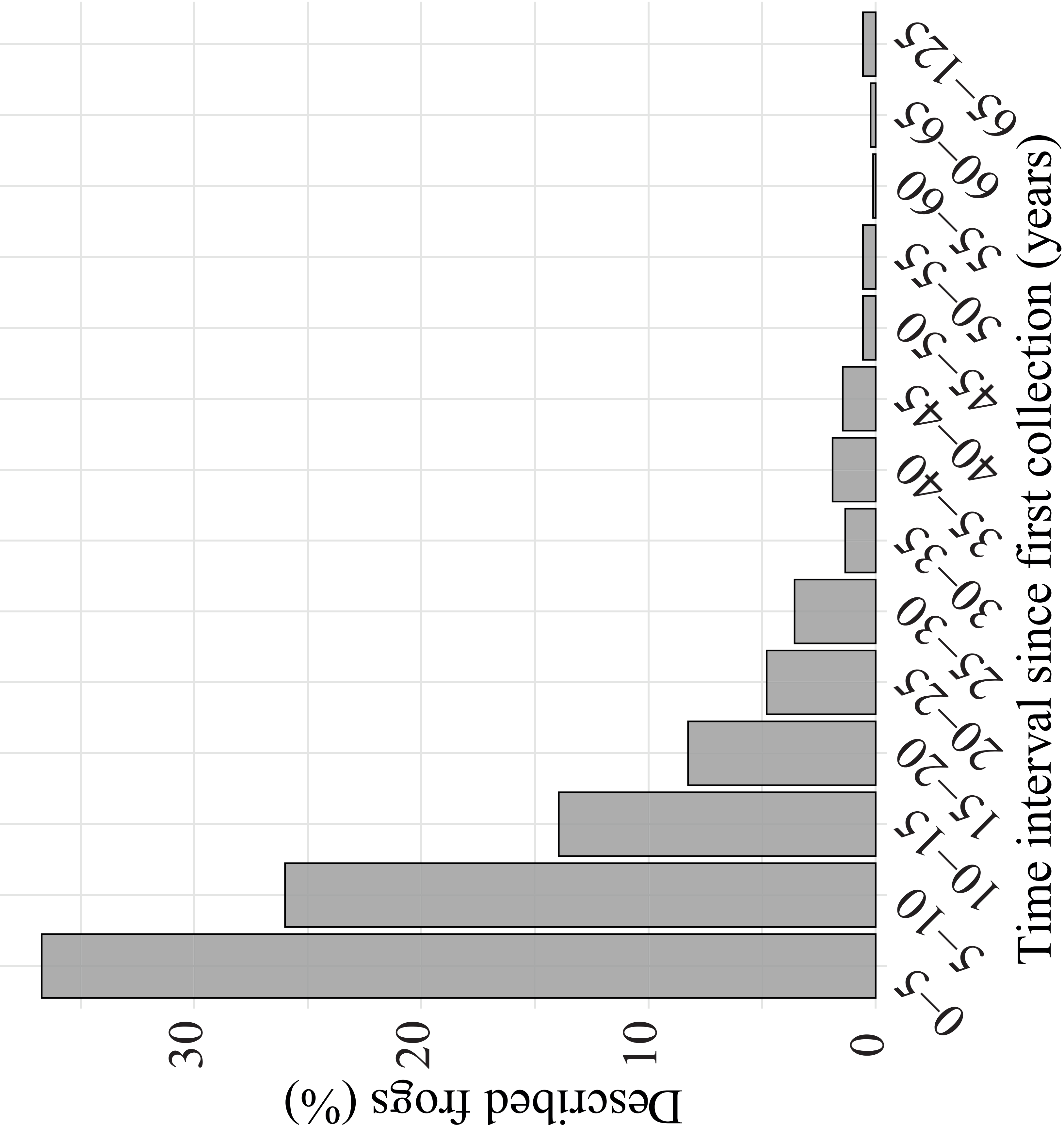

Figure 2

Click here to access/download;Figure;3_Fig_2.eps

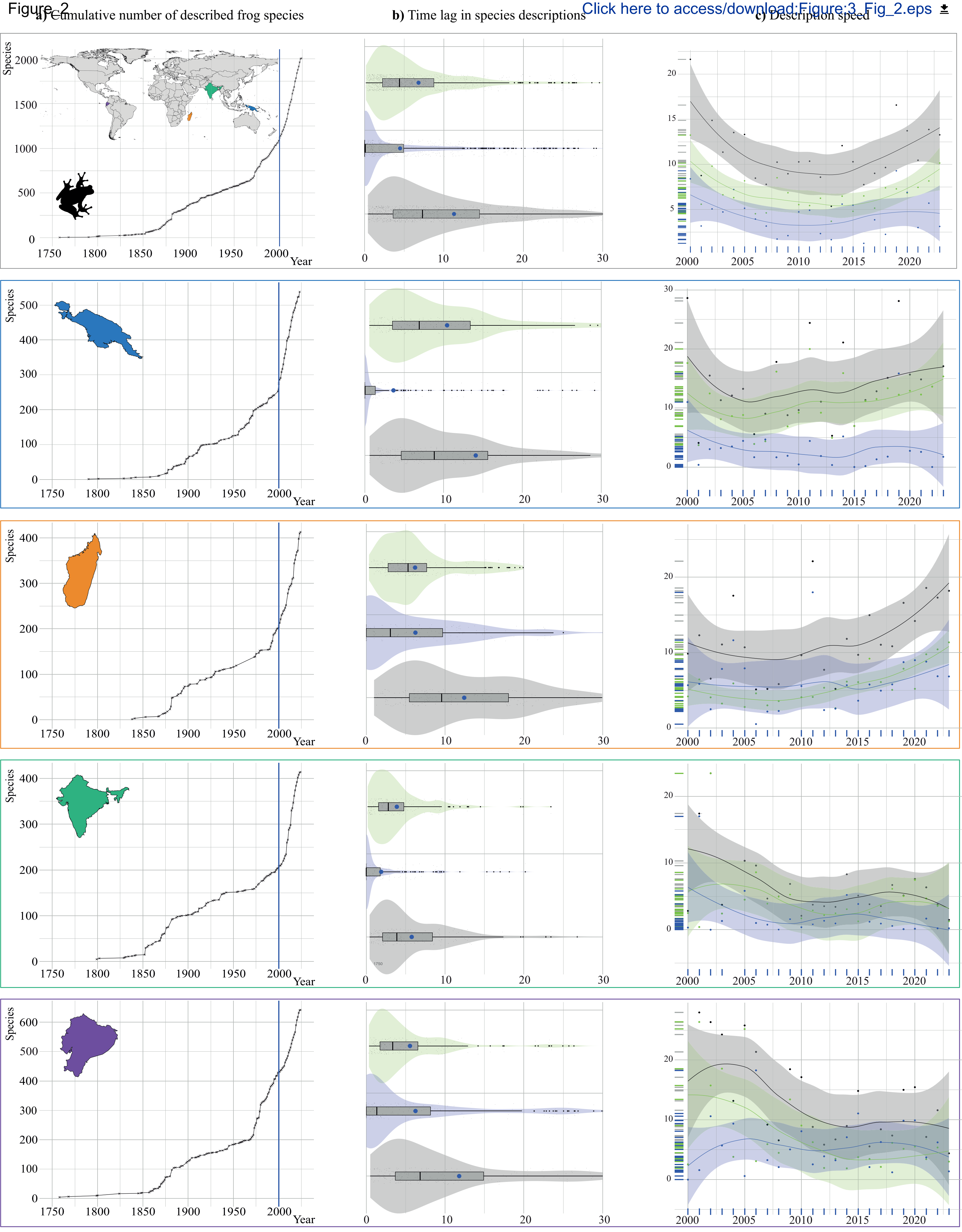

Figure_3

Click here to access/download;Figure;4_fig_3.eps

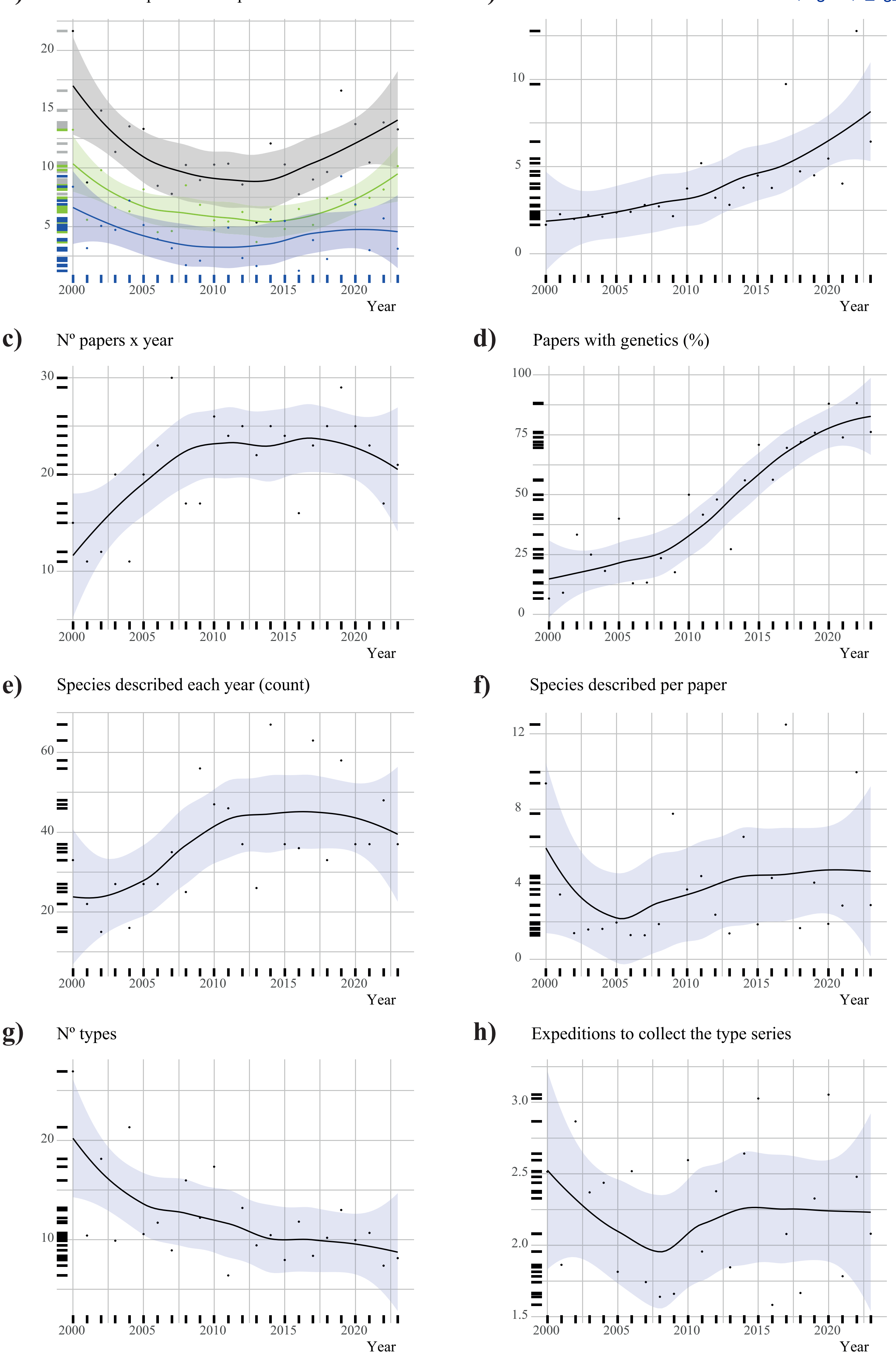

S1_Fig

Click here to access/download
Supporting Information
5_S1_Fig.pdf

S2_Fig

Click here to access/download
Supporting Information
6_S2_Fig.pdf

S3_Fig

Click here to access/download
Supporting Information
7_S3_Fig.pdf

S4_Fig

Click here to access/download
Supporting Information
8_S4_Fig.pdf

S5_Fig

Click here to access/download
Supporting Information
9_S5_Fig.pdf

S6_Fig

Click here to access/download
Supporting Information
10_S6_Fig.pdf

S7_Fig

Click here to access/download
Supporting Information
11_S7_Fig.pdf

S8_Fig

Click here to access/download
Supporting Information
12_S8_Fig.pdf

S9_Fig

Click here to access/download
Supporting Information
13_S9_Fig.pdf

S10_Fig

Click here to access/download
Supporting Information
14_S10_Fig.pdf

S11_Fig

Click here to access/download
Supporting Information
15_S11_Fig.pdf

S1_Table

Click here to access/download
Supporting Information
16_S1_Table.xlsx

S2_Table

Click here to access/download
Supporting Information
17_S2_Table.xlsx

S3_Table

Click here to access/download
Supporting Information
18_S3_Table.pdf

S4_Table

Click here to access/download
Supporting Information
19_S4_Table.pdf

Title: Croaking for haste: How long does it take to describe a frog species since its discovery?

Authors: Albert Carné[1, 2, 3*], Alberto Sánchez-Vialas[2], Claudia Lansac[2, 3], Miriam Moreno[5, 6], Ignacio De la Riva[2*]

Corresponding authors: albert.carne@mncn.csic.es (AC), iriva@mncn.csic.es (IDLR)

Short title: Croaking for haste

Affiliations:

[1]Science and Business S.L., Edificio CITEXVI, Vigo, Spain.

[2]Department of Biodiversity and Evolutionary Biology, Museo Nacional de Ciencias Naturales (MNCN), CSIC, Madrid Spain.

[3]Department of Biodiversity Ecology and Evolution, Faculty of Biological Sciences, Complutense University of Madrid, Madrid, Spain.

[4]Biodiversity Research Institute (IMIB), University of Oviedo-Principality of Asturias-CSIC, Mieres, Spain.

[5]Department of Biology of Organisms and Systems, University of Oviedo, Oviedo, Spain.

ORCID IDs:

Albert Carné: https://orcid.org/0000-0002-7920-8159

Claudia Lansac: https://orcid.org/0000-0002-6082-1252

Alberto Sánchez-Vialas: https://orcid.org/0000-0003-0068-7669

Miriam Moreno: https://orcid.org/0009-0009-1568-4795

Ignacio De la Riva: https://orcid.org/0000-0001-5064-4507

Abstract

Global biodiversity faces severe anthropogenic threats, with alarming extinction rates projected for the near future. Most of Earth's diversity remains undescribed, meaning countless species are doomed to extinction before being documented. Since current conservation laws typically consider only described species, the lag time ~~to~~ in achievinge a representative ~~inventory of~~ global biodiversity inventory is a crucial issue impeding~~for~~ effective conservation. Amphibians, the most endangered vertebrate class, exemplify the challenge: while the number of threatened species rises, new species descriptions rapidly increase, and hundreds of candidate species are flagged annually~~ worldwide~~. We analyzed all anuran species described from ~~the year~~ 2000 to 2023 across four biodiversity-rich tropical regions to investigate the time required to describe new frog species. We quantified the time needed to collect the type series, the number and timing of expeditions, the lag between collection and publication~~ of the species description~~, and the total time between first specimen collection and article publication. Additionally, we explored temporal trends and the effect of selected abiotic variables. On average, it takes 11.3 years to formally describe a frog species since the collection of the first specimen, with 4.5 years spent ~~on specimen collection~~collecting the type series and 6.8 years on description and publication. These figures were consistent across three of the four regions analyzed. Alarmingly, the time required to describe new species is globally increasing, exacerbating the so-called taxonomic impediment. Thirty-six percent~~Only 36%~~ of species were ~~described~~ named within five years of first collection,

highlighting the ~~importance ~~need ~~of ~~for continued collecting, biological collections as reservoirs of undescribed diversity, and calling for~~ while also calling for~~ specimen revision after expeditions. These results raise concerns about the effectiveness of current taxonomic and conservation practices ~~in ~~addressing the biodiversity crisis. We call for a global effort to prioritize taxonomic research and discuss ~~innovative ~~taxonomic and conservation approaches. Under current practices, and given the observed timelines, we will lose the race against extinction for many species.

Introduction

Global biodiversity is being assaulted by Global Change. Wildlife ~~Populations ~~populations are being reduced, contracted, and extirpated; species are becoming extinct at an unprecedented rate, and entire ecosystems are disappearing before we have the opportunity to properly ~~prospect~~ document them [1–4~~1–4~~]. The loss of a species is irreversible and its consequences are unpredictable [5~~5~~]. The highest rates of decline and extinction are occurring in the tropics, hyper diverse areas where scientific knowledge remains limited, ~~as ~~but which~~they~~ are known to contain high levels of undiscovered and undescribed diversity [3, 6–10~~3, 6–10~~].

Species are the fundamental unit to measure biodiversity. Only by recognizing the actual species richness of a given region can we reliably ~~discover ~~understand large-scale ecological and evolutionary trends (e.g., [11~~11~~]). How many species are on Earth remain an unresolved question —~~—~~the Linnean shortfall [12–13~~12–13~~], partly because of incomplete sampling and the lack of robust biodiversity characterization and extrapolation approaches [14–15~~14–15~~]. However, there are three aspects of consensus: first, the largest proportion of biodiversity is comprised by undescribed species [15~~15~~];~~.~~ s~~S~~econd, the urgency to discover and describe this diversity has never been greater —~~; ~~naming species is ~~the ~~a first step towards their conservation, and overcoming the Linnean shortfall is crucial for understanding distributional patterns and, consequently, for reaching effective conservation planning [16–17~~16–17~~];~~.~~ t~~T~~hird, we must explore new taxonomic approaches, as the estimated time required to document the remaining biodiversity far exceeds what may be available due to ongoing biodiversity threats [18–19~~18–19~~].

Traditional taxonomic approaches, largely based on morphology, face significant challenges in addressing the urgency of the current biodiversity crisis. Integrative taxonomy emerged to accommodate and unify new concepts and methods from various disciplines to ensure the proper description of taxa, taxonomic stability over time, and preventing taxonomic inflation by integrating multiple lines of evidence [18, 20~~18, 20~~]. A "fast-track" taxonomy proposal arose from the pressing need to characterize and describe as much diversity as possible before it disappears, significantly shortening the description time by deliberately omitting previously collected material and reducing the most time-consuming parts of the process (i.e., high-quality images vs. detailed and extensive descriptions, reducing descriptions vs. enhancing~~e~~ diagnoses) [21–22~~21–22~~]. However, considering the overwhelming number of candidate species awaiting description worldwide (e.g., [10, 23–32~~23–31~~], but see [33]), the global richness estimates [15~~15~~], and the escalating biodiversity crisis, this strategy is likely unable to keep pace with current and projected extinction rates [34~~32~~], nor to achieve a representative inventory of global species within a reasonable time frame [22~~22~~]. Some authors, not without receiving criticisms, have attempted the so-called "turbo-taxonomy", which consists in describing large numbers of taxa providing minimal information on each species, sometimes even just a barcoding sequence (e.g., [35~~33~~]). While this approach may help overcoming the Linnean shortfall, questions arise about the usefulness and reliability of such species "descriptions" [36~~34~~]. Although "fast-track" and "turbo" taxonomy are sometimes seen as synonyms [36~~34~~], we distinguish them as two different approaches. We consider as "fast-track" those descriptions that, while concise, include all necessary sections to allow a third party to identify the species without the need for a molecular lab (e.g., [37~~35~~]), while "turbo" are those minimalist descriptions that do not (e.g., [35~~33~~]).

Regardless of the chosen approach, describing a species involves five steps~~, each depending on the previous one~~ —the five D's of taxonomy [38~~36~~]: discovery, delimitation, diagnosis, description and specimen determination (although not necessarily by the same person). While

these steps are conceptually linear, in practice, the process is often iterative, with researchers revisiting and redefining previous steps as new information emerges or as the species boundaries are reassessed. None of these steps can be achieved without previous field work. Fieldwork ~~campaigns are~~is time-consuming and, particularly in tropical regions, can be challenging, expensive, and logistically complex. To decide whether a lineage merits to be named, data from different lines of evidence (e.g., morphology, genetics, bioacoustics, ecology) should ideally be gathered, which requires time and cannot always be achieved within a single ~~campaign~~field trip. Compiling such evidence, preferably including multiple specimens for the type series, can be challenging when cryptic, secretive, seasonal, narrowly-distributed species, or those with low population densities are involved. This is especially true for frogs, where obtaining call recordings —often a source of key diagnostic characters— can be daunting. Specimens representing populations that clearly merit ~~to be formally named~~recognition as distinct species can remain unstudied ~~in~~ on shelves for years [39~~37~~], which negatively impacts their conservation prospects, as undescribed species are more vulnerable to extinction [40~~38~~].

Amphibians are the most endangered vertebrate class worldwide. More than 41% of described species are under threat, and the number of species at high risk of extinction continues to increase [41~~39~~]. Threats are ubiquitous throughout the amphibian tree of life, with habitat loss and degradation (primarily due to deforestation), climate change, and emerging diseases being the greatest risks to amphibian persistence [41~~39~~]. There is a geographical bias, with those regions with greater diversity and lesser available information, i.e., the tropics, being the most affected [3, 42–43~~3, 40–41~~]. Paradoxically, current rates of amphibian species description are ~~exponential~~ high (ca. 150 sp. nov./year) and, during the last decade, hundreds of further candidate species have been flagged globally [10, 30–32~~10, 30–31~~]. This indicates that we are still far from overcoming the Linnean amphibian shortfall, that the number of threatened species has likely been underestimated, and that many species are facing silent extinction before they are named or even discovered [44].

Here we analyze the type series metadata of all anuran species described since the year 2000 from four tropical regions, to globally and regionally estimate: (1) how long it takes to describe a frog species since the first type specimen is collected using either traditional or contemporary methodologies~~?~~; (2) how long it takes to get the complete type series for describing a frog species~~?~~; (3) how long it takes to describe a frog species since all the type material has been collected~~?~~; (4) how many field ~~campaigns~~ trips are needed to describe a frog species~~?~~; (5) ~~are~~ whether ~~the~~ description times are decreasing or increasing over time~~?~~; and (6) ~~what~~ the abiotic factors that ~~determine~~ are related to~~that~~ some frogs being~~are~~ described in a ~~notorious~~ notably shorter time than others, and how ~~have~~ these factors have evolved over time.~~?~~

Materials and methods

This study did not involve any experiments or direct interaction with live animals. All data analyzed were obtained exclusively from previously published literature. Therefore, ethical approval from an Institutional Animal Care and Use Committee (IACUC) or other ethics board was not required for this research.

Region selection, data acquisition and curation

We selected four regions across the globe based on two criteria: high frog species richness (i.e., > 300 species), and an ~~current~~ exponential rate of accumulated frog species descriptions. The regions chosen were Ecuador, India, Madagascar, and Melanesia~~ (Fig 1)~~. These regions are taxonomically independent and, in principle —and largely in practice~~—~~, do not share taxonomists, which helps ~~minimize~~ control for regional biases in research practices. By selecting geographically scattered regions, we aim to investigate the time span of the species description process on a global scale, diluting regional biases to obtain a more accurate timeframe that can be considered globally applicable.

~~**Fig 1. Time lags and trends in anuran amphibian species descriptions.** (**a**) Cumulative number of frog species described over time. (**b**) Time lags in the description process. (**c**) Average description speed per year. Green: description *sensu stricto*, blue: collection, gray: overall process.~~

We retrieved the list of described frog species for each region, up to December 2023, from Amphibian Species of the World (ASW; [45~~42~~]), filtering by higher taxa (Anura) and country, and retaining only native species (i.e., discarding th~~ose~~ose introduced). Since Melanesia includes two countries —Indonesia and Papua New Guinea—, and parts of Indonesia extend well beyond the region, we could not easily obtain the species list from the online database. Therefore, in accordance with the definition of the Melanesian region provided by Oliver et al. [31~~31~~], we compiled the species list by selecting “Indonesia-Papua Region”, “Papua New Guinea”, “Solomon Islands”, and “Fiji” (which also includes smaller nearby islands) in ASW.

To determine the ~~current~~ species description lags for a contemporary timeframe~~(i.e., using contemporary methodologies)~~, we filtered each regional species-list by year, retaining only those species described from the year 2000 (included) onward. We decided to exclude data predating 2000~~,~~ as software and genetic data —assumed to accelerate species discovery, diagnosis, and description— ~~have become~~became widely available and utilized (with regional variability) only in the 21st century. Genetic techniques have become crucial for describing species, but also for detecting cryptic species and screening regions (and public collections) to unveil overlooked and cryptic diversity (e.g., [10, 30~~10, 30~~]).

For each species, we retrieved the publication and all collection dates of the type series (holotype and paratypes) from the original descriptions, excluding additional and referred specimens. We only considered unique collection dates; that is, if multiple specimens were collected on the same day, we registered only one date. To deal with imprecise dates (e.g., 5–~~-~~7 December 2008, February 2007) we used the midpoint date (e.g., 6 December 2008 and 14 February 2007, in the provided examples).

Lag times

We made several necessary assumptions regarding the species description process, and defined three time periods: “overall description process”, “collection”, and “description *sensu stricto*”. We acknowledge that defining these periods and making these assumptions is an oversimplification, but it is necessary to draw conclusions (see Discussion).

We consider the “overall description process” as the time span between the collection date of the first (i.e., the oldest) specimen included in the type series and the publication date of the article introducing the new species to the scientific community. Within this overall process, we differentiate the two other sub-periods, the “collection” and the “description *sensu stricto*”.

The collection period refers to the fieldwork ~~campaigns~~ conducted to gather the entire type series, extending from the collection of the first specimen to the last. We assume that all type specimens were considered essential for the species description, which was delayed until the completion of the type series, and that the inclusion of both recently collected and older specimens was not arbitrary. Additionally, we regard the first collected type specimen as the first scientific encounter with the species (i.e., the moment a researcher truly discovers the species), even if it was not immediately recognized as a new taxon in the field and this insight occurred years later.

The description *sensu stricto* is the process encompassing taxon delimitation, diagnosis, description, and determination (*sensu* [38~~36~~]), spanning from the collection of the last specimen of the type series to the publication date. At this point, we assumed that once the last specimen of the type series was collected, the description *sensu stricto* started.

To summarize and visualize the three periods, we calculated their durations in years for easier interpretation and representation. Using the *base* package in R [46], we obtained central tendency

statistics including the mean, trimmed mean (mean after excluding 10% of extreme values from both ends to reduce the influence of outliers), and median, along with position statistics such as the minimum and maximum. Using the *psych* package [47], we then calculated measures of asymmetry, including skewness (an asymmetry coefficient where positive values indicate a long right tail, meaning most observations are concentrated below the mean with some high values) and kurtosis (a measure of tail weight and peakedness where high values indicate heavy tails and a sharp central peak compared to a normal distribution). Using the *psych* package, we also calculated dispersion measures such as the Standard Deviation (S.D.), Median Absolute Deviation (M.A.D.; a robust dispersion measure; calculated as the median of absolute differences from the median, less sensitive to outliers than S.D.), range, and Standard Error (S.E.). ~~We obtained the central tendency and position statistics of each period using the *base* package in R [43], and we calculated asymmetry and dispersion statistics using the *psych* package [44].~~ We used the *ggplot2* package [48~~45~~] to represent the results using violin and boxplots. This process was conducted independently for each genus and region, followed by ~~and~~ merging the four regional datasets to obtain global estimates.

Number of field ~~campaigns~~ trips and temporal patterns of type specimen collection

To estimate the number of field ~~campaigns~~ trips required to collect the entire type series for each species description, we calculated the time span between the sorted collection dates of the specimens in the type series. We considered as distinct field ~~campaigns~~ trips those collection events separated by more than one month (i.e., 30 days).

To estimate global and regional temporal patterns in the collection of type specimens (i.e., the number of field ~~campaigns~~ trips conducted to collect type specimens each month and year), we calculated the number of specimens collected per month and year, and represented the collection frequencies using heatmaps with the *ggplot2* package.

Effect of abiotic variables on the description lags

To understand why certain species were described faster than others, we investigated the effect of several abiotic variables on the description *sensu stricto*. We did not analyze the effect of these variables on the overall and collection processes, as the values of these variables are typically determined post-collection.

We included three categorical variables: (1) whether genetics were involved in the species description (YES/NO), (2) the region, and (3) the genus; also, we considered five continuous variables: (1) the number of authors ~~on~~ of the description paper, (2) the number of ~~type~~ specimens in the type series, (3) the number of species described in each paper, (4) the number of field ~~campaigns~~ trips required to collect the type series (see above), and (5) the number of species currently recognized in the genus.

Before fitting the models, we ensured that no numerical variable had a correlation greater than 70% with any other to avoid multicollinearity. For each of the four regions and the global analysis (i.e., combining the four regions into a single dataset), we fitted generalized linear mixed models (GLMM) using the *glmmTMB* R package [49~~46~~]. In the global model, we included "genus" and "region" as random effects to account for the hierarchical structure of the data. We excluded "region" in the regional models. All models used a Gaussian family with an "identity" link function. We log-transformed the response variable to meet or improve the model assumptions.

To further assess the significance of the relationships, we performed an analysis of variance using the *Anova()* function from the *car* R package [50~~47~~], which allowed us to evaluate the influence of the predictor variables without considering the factor levels.

We evaluated the model performance using the *DHARMa* package [51~~48~~], by obtaining the simulated residuals from the model to assess the deviation from the expected distribution, uniformity, outliers, and dispersion. Additionally, we fitted null models (i.e., only including the

random effects) and compared these with the empirical models to determine whether the inclusion of fixed effects in the empirical model, provided a significantly better fit to the data. We conducted this comparison using *anova()* from the *stats* package. Finally, we used the *effects* package [50~~47~~] to represent the effects of the variables.

Temporal evolution of variables

For each region and globally, ~~W~~we evaluated the temporal evolution of several variables including the overall description process, the collection, the description *sensu stricto*, the number of authors, the number of species described each year, the number of taxonomic papers published annually, the number of species described in each paper, the number of type specimens in each species' type series, the percentage of species described using genetics each year, and the number of field ~~campaigns~~ trips undertaken to collect the type series.

To analyze the temporal evolution of these variables, we averaged the yearly values for each region and globally (e.g., the mean time invested in the collection of type specimens for each species described in ~~2004~~2006).~~;~~ ~~also~~Additionally, we counted the number of species described each year and the number of those that used genetic data, in order to compute the percentage of species descriptions incorporating genetic evidence per year.~~, we counted the number of species described each year and the number of species described using genetics to compute the percentage.~~

We used the *ggplot2* package to generate all plots, applying local regressions (LOESS) to fit curves to the data for visualization purposes. To assess the temporal significance of the aforementioned variables, we calculated *p-values* using linear (LM, lm()) or generalized additive models (GAMs, gam()) from the *stats* package with default parameters [46~~43~~]. We selected the best method based on the comparison of their coefficient of determination (R^2).

Results

Species described since the year 2000 and lag times

Nine hundred and twenty-four frog species have been described across the four analyzed regions between 2000 and 2023. Melanesia leads with 286 newly described frog species, followed by Madagascar with 215, Ecuador with 213, and India with 210. This represents an average description rate of ca. 10 new species per region each year. After excluding unavailable papers and species with unreported collection or publication dates, we analyzed a total of 2,981 unique collection dates from 896 species; 269 species from Melanesia, 215 from Madagascar, 208 from Ecuador, and 204 from India (S1 Table and S2 Table).

Thirty-six percent of the analyzed species were described within ~~the first~~ five years ~~after~~ of the collection of the first type specimen, and ca. 26% of the species between five and ten years ~~later~~of the first collection. The remaining ca. 40% of the species were described between ~~ten~~ 10 and 125 years after the first type specimen collection (Fig 1~~2~~).

Fig 1~~2~~. Percentage of frog species described since their first collection. Percentage of frog species described as a function of the time elapsed since the collection of the first specimen.

The mean and confidence interval for the global overall description process (i.e., from the collection of the first type-specimen to the paper publication date) is 11.3~~26~~ ± 0.8~~2~~ years, with 4.5~~47~~ ± 0.7~~65~~ years dedicated to collecting the type series and 6.8~~79~~ ± 0.5~~49~~ years focused on the description *sensu stricto* (see Fig 2~~1~~ and Table 1 for central tendency, position, asymmetry, and dispersion statistics).

Fig 21. Time lags and trends in anuran amphibian species descriptions. (**a**) Cumulative number of frog species described over time. (**b**) Time lags in the description process. (**c**) Average description speed per year. Green: description *sensu stricto*, blue: collection, gray: overall process. The world map showing country boundaries was obtained from OpenDataSoft (https://public.opendatasoft.com/explore/dataset/world-administrative-boundaries/information/) and is licensed under the Open Government Licence v3.0, which is compatible with the Creative Commons Attribution License (CC BY 4.0).

Table 1. Summary statistics for each analyzed region across the different subperiods of the species description process. The table presents the minimum (Min.), mean, standard deviation (S.D.), 10% trimmed mean (Trim. mean), median, median absolute deviation (M.A.D), maximum (Max.), range, skewness (Skew), kurtosis, standard error (S.E.), and 95% confidence interval for the mean (Interval).

	GLOBAL	Ecuador	India	Madagascar	Melanesia
**Species**	896	208	204	215	269
**Overall description process**					
Min.	0.42	0.5	0.42	0.99	0.61
Mean	11.26	11.79	5.77	12.43	14.08
S.D.	12.63	12.58	6.16	10.48	16.18
Trim. mean	8.91	9.43	4.78	11.25	11.01
Median	7.28	6.85	3.87	9.57	8.81
M.A.D	6.59	5.86	3.4	7.84	7.59
Max	125.74	82.95	60.04	110.86	125.74
Range	125.32	82.45	59.62	109.88	125.13
Skew	3.5	2.03	4.08	4.16	3.22
Kurtosis	20.48	5.17	28.75	34.34	15.31
S.E.	0.42	0.87	0.43	0.71	0.99
Interval (95%)	11.26 ± 0.82	11.79 ± 1.71	5.77 ± 0.85	12.43 ± 1.40	14.08 ± 1.93
**Description *sensu stricto***					
Min	0.18	0.33	0.18	0.35	0.57
Mean	6.79	5.54	3.88	6.19	10.45
S.D.	7.52	6.8	3.6	4.46	10.3
Trim. Mean	5.33	4.15	3.27	5.56	8.43
Median	4.41	3.35	2.81	5.3	6.92
M.A.D	3.74	2.83	2.37	3.72	6.33
Max	54.62	51.48	23.44	19.91	54.62
Range	54.45	51.14	23.26	19.56	54.05
Skew	2.85	3.27	2.21	1.21	1.96
Kurtosis	10.57	14.09	6.8	0.82	3.93
S.E.	0.25	0.47	0.25	0.3	0.63
Interval (95%)	6.79 ± 0.49	5.54 ± 0.92	3.88 ± 0.49	6.19 ± 0.60	10.45 ± 1.23
**Collection**					
Min	0	0	0	0	0
Mean	4.47	6.25	1.89	6.24	3.64
S.D.	9.93	10.58	5.13	9.96	11.54
Trim. Mean	2.2	3.92	0.75	4.52	0.92
Median	0.08	1.32	0	3.07	0.01
M.A.D	0.12	1.96	0	4.56	0.02
Max	104.93	79.97	55.84	104.67	104.93
Range	104.93	79.97	55.84	104.67	104.93
Skew	4.94	2.84	6.54	5.05	5.51
Kurtosis	35.46	11.73	59.3	43.02	36.14
S.E.	0.33	0.73	0.36	0.68	0.7
Interval (95%)	4.47 ± 0.65	6.25 ± 1.44	1.89 ± 0.70	6.24 ± 1.33	3.64 ± 1.38

We found significant disparities among regions. India leads in the speed of species descriptions, averaging 5.8~~77~~±0.9~~85~~ years of overall process, 1.9~~89~~±0.7~~0~~ years for collection, and 3.9~~88~~±0.5~~49~~ years for description. Ecuador follows with an overall process time of 11.~~79~~8±1.7~~1~~ years, 6.3~~25~~±1.44 years for collection, and 5.54±0.9~~2~~ years for description. Madagascar closely trails, with an overall process of 12.4~~3~~±1.4 years, comprising 6.24±1.3~~3~~ years for collection and 6.2~~19~~±0.6~~0~~ years for description. Finally, the region that takes the longest is Melanesia, with an overall process time of 14.1~~08~~±1.9~~3~~ years, including 3.64±1.4~~38~~ years for collection and 10.5~~45~~±1.2~~3~~ years for description (Fig 2~~1~~ and Table 1).

In all three periods (i.e., overall, collection, and description) and across all five regions (i.e., four independent regions, plus global), we obtained high standard deviation values, with values exceeding the mean value in 11 out of 15 cases. This indicates a significant dispersion of the data around the mean. The median values consistently fell below the mean, suggesting a skewed distribution. Skewness values were greater than zero in all cases, pointing to positive asymmetry. Kurtosis values consistently exceeded zero, signaling heavy tails and the presence of more extreme values (outliers) that would typically occur in a normal distribution. Additionally, the standard error values remained low in all cases, especially in comparison to the standard deviation, indicating that the obtained mean is a fairly reliable estimate of the population mean.

Number of field ~~campaigns~~ trips and temporal patterns of type specimen collection

Half of the species (476; 53.1~~3~~%) described between 2000 and 2023 required a single field ~~campaign~~ trip to collect the entire type series, with an average of eight collected specimens per species. Two trips were required for 20.3% of species, three for 11.7%, and four for 6.1%. The remaining ca. 8% were described on the basis of collections made over between five and 23 field trips.~~For 20.31% of species, two field campaigns were necessary, while 11.72% required three expeditions, and 6.14% required four. The rest (ca. 8%) of species needed between five and 23 field campaigns.~~

Considering the four regions, species descriptions between 2000 and 2023 were based on type specimens collected between 1893 and 2023, with the majority collected between 1999 and 2016, with some regional differences (S1-S5 Fig).

In Melanesia and Ecuador (S4-S5 Fig), type specimen collection was relatively evenly distributed throughout the year (defined as each month having at least 40% of the number of specimens collected in the peak month). In contrast, Madagascar and India exhibit marked seasonal peaks in collection activity, with certain months showing higher numbers of type specimens collected than others (S2-S3 Fig).

~~Considering the four regions altogether, species descriptions between 2000 and 2023 have been based on type specimens collected between 1893 and 2023. With over 60 type specimens collected each year, the years comprised between 1999 and 2016 show the highest concentration. Notably, February has been the peak month for type specimen collections, totaling 412 type specimens, followed by July with 277, November with 257, and June with 254 (S1 Fig).~~

~~In Madagascar, species descriptions have been based on specimens collected between 1900 and 2022, with a peak collection period from 2003 to 2007, during which more than 45 specimens were collected annually. February is, by far, the month with the highest number of type specimens collected, totaling 239 type specimens, followed by January with 125, November with 120, and March with 114 (S2 Fig).~~

~~Species descriptions in India have been based on specimens collected between 1954 and 2023, with the highest concentration between 2009 and 2012, with more than 25 specimens collected per year. June and July are the months with the highest number of type specimens collected, with a total of 82 type specimens collected in each month, followed by September with 53, August with 48, and May with 40 (S3 Fig).~~

~~In Melanesia, species descriptions have been based on specimens collected between 1893 and 2018, with the highest concentration between 2002 and 2005, with more than 65 specimens collected per year. April, May, September, and October are the months with the highest number of type specimens collected with between 104 and 109 specimens collected each month, followed by August with 85, July with 69, and November with 66 (S4 Fig).~~

~~Finally, the type specimens on which the species descriptions were based in Ecuador were regularly sampled between 1933 and 2022. The highest collection concentration is between 2000 and 2012, with more than 30 type specimens collected each year. July is the month with the highest number of type specimens collected with 110 type specimens, followed by February with 101, January with 86, and April with 82 (S5 Fig).~~

Effect of abiotic variables on the description lags

Empiric models differed significantly from the null models and presented lower AIC values, indicating that the models are better than randomness and that the fixed variables have a genuine effect on the data (S3 Table). All models met the model assumptions, except for Ecuador, where minor violations were observed (S6 Fig). Adding random effects ("genus" in all cases, and both "genus" and "region" in the global analysis) significantly increased the percentage of variance explained by the models (S4 Table~~, S6 Fig~~).

In all regions except Ecuador, the number of authors had a significant effect on species description time, with an increase in the number of authors associated with a faster description in the global model, Ecuador, and India, and with slower description times in Madagascar and Melanesia. The number of type specimens did not have a significant effect in any region. The number of described species within a genus was marginally significant in Madagascar, where richer genera are associated with faster species descriptions. This variable was not significant in other regions. The number of field ~~campaigns~~ trips significantly influenced~~s~~ the description times in the global model and in India, was marginally significant in Ecuador and Madagascar, and showed no significant effect in Melanesia. Across all regions, however, an increase in the number of field ~~campaigns~~ trips to collect the type series was associated with shorter description times, perhaps because much of the work had already been done and only complementary data, such as vocalizations, were needed. The number of species described per paper was significant in the global model, Ecuador, and Melanesia, but not in Madagascar or India. An increase in the number of species per paper was associated with longer description times in all regions except in Madagascar, where the effect was the opposite (but not significant). The use of molecular data in species descriptions ~~was~~ had a significant effect in the global model and in India but not in Ecuador, Madagascar, or Melanesia. In all regions except Madagascar, incorporating genetic data was associated with longer description times (S7 Fig, S4 Table).

Temporal evolution of variables

Globally, the overall description process decreased from 16 years in 2000 to 7.2 years by 2012. However, after 2012, the description lag increased again, reaching a current value of ca. 11 years (Fig 3). Both collection and description *sensu stricto* followed similar declining trends until 2012, after which the description time rose significantly, while collection time remained stable. Statistical analyses showed that both overall description time and description *sensu stricto* varied significantly with the year (p-value$_{GAM}$: 0.023 and 0.015, respectively), while collection time remained stable (non-significant). ~~In general, the overall description process has shortened since 2000, but recent trends suggest an ongoing increase (Fig 3).~~

Fig 3. Temporal evolution of variables in the four analyzed regions. (**a**) Overall description process (black), collection (blue), and description *sensu stricto* (green), (**b**) ~~Nº~~ number of authors in each species description (mean), (**c**) number~~Number~~ of published taxonomic papers (count), (**d**) Taxonomic papers that include genetics (percentage), (**e**) number~~Nº~~ of species described each year (count), (**f**) number~~Nº~~ of species described in each paper (mean), (**g**) number~~Nº~~ of type

specimens in the type series (mean), (**h**) number~~№~~ of field ~~campaigns~~ trips conducted to collect the entire type series (mean).

Ecuador, India, and Melanesia broadly follow the global pattern in the overall description (p-value$_{GAM}$: 0.01, 0.03, and n.s., respectively), in the description *sensu sticto* (p-value$_{GAM}$: 0.01, n.s., and n.s. respectively), and in the collection processes (n.s.). In contrast, Madagascar shows a considerable increase in both the overall description process and description *sensu stricto* (p-value$_{GAM}$: 0.03 and <0.001, respectively), as well as a moderate, non-significant increase in the collection time (S8-S11 Fig).

~~Ecuador and India have shown reductions in the overall description process between 2000 and 2012, after which they remained stable until recent declines (p-value$_{GAM}$: 0.01 and 0.03, respectively). The description *sensu sticto* followed the same pattern as the overall description process in both regions (p-value$_{GAM}$: 0.01 and non-significant, respectively). In India, the collection time has non-significantly decreased, while in Ecuador, it has remained stable. In contrast, Madagascar shows a considerable increase in both the overall description process and description *sensu stricto* (p-value$_{GAM}$: 0.03 and <0.001, respectively) as well as a moderate non-significant increase in the collection time. Melanesia shows a similar pattern to global trends but non-significant, with an initial decrease followed by an increase in both the overall process and description *sensu stricto*, while the collection time has slightly declined (S8-S11 Fig).~~

The number of authors contributing to species descriptions has significantly increased globally (p-value$_{LM}$: <0.001). In Ecuador and India, the number ~~of authors~~ rose from ca. two to ~~ca.~~ five authors, where it appears to have ~~reached a~~ plateaued (p-values$_{GAM}$: <0.001), while~~. In contrast, the number of authors~~ in Madagascar and Melanesia, it continues to rise (p-values$_{GAM}$: <0.006) (S8-S11 Fig).

The number of taxonomic papers published globally each year increased from ca. 12 to ~~ca.~~ 23, ~~where it remained stable~~plateaued until 2020, ~~when it began to decline again~~and then declined (p-value$_{GAM}$: 0.01). Ecuador, ~~and~~ India, and Melanesia ~~show~~ follow similar patterns (p-values$_{GAM}$: <0.001, <0.001, and n.s.). In contrast, ~~the number of papers in~~ Madagascar shows high variability, with an overall mean of ca. five annual papers ~~per year~~, ~~and~~ with no significant trend ~~. In Melanesia, there was also an increase followed by a decrease, but there is not a significant trend~~ (S8-S11 Fig).

The proportion of taxonomic papers incorporating genetic data has significantly increased globally, reaching 90~~5~~% ~~of published papers using genetics in~~by 2022 (p-value$_{GAM}$: <0.001). Madagascar shows the earliest widespread ~~usage of genetics~~usage, with 100% ~~of published~~of papers incorporating genetic data since 2004, with few exceptions (p-value$_{GAM}$: <0.001). In India, the use of genetics remained low (ca. 25%) until 2010,~~-~~ followed by a~~when only 25% of papers incorporated genetic data. Since then, there has been a marked~~n ~~-~~increase to the current 100% ~~of usage~~ (p-value$_{GAM}$: <0.001). In Ecuador, usage ~~the usage of genetics~~ has increased steadily, currently exceeding 80% (p-value$_{GAM}$: <0.001). In contrast, Melanesia lags behind ~~the other regions~~, ~~using~~ with ~~genetics in~~ only ca. 40% of ~~the published~~ papers incorporating genetic data ~~, although there has been a modest increase~~ (p-value: 0.005) (S8-S11 Fig).

The number of species described ~~each year~~annually increased globally from ca. 25 to ~~ca.~~ 45 ~~species~~ until 2010, when it ~~reached a plateau~~plateaued (p-value$_{GAM}$: 0.01).~~ A~~ Ecuador shows a similar trend~~pattern is observed in Ecuador~~, ~~where the plateau was reached~~reaching a plateau of ca. 12 species per year by~~in~~ 2018~~, with ca. 12 species described annually~~ (p-value$_{GAM}$: <0.001). India shows a non-significant steady increase until 2012, followed by a decline. Madagascar and Melanesia show great variability with an overall non-significant continuous increase without reaching a plateau in Madagascar and an overall decrease in Melanesia (S8-S11 Fig).

The average number of species described per paper shows a globally non-significant positive ~~relationship~~trend ~~with the year~~. Ecuador and Madagascar ~~exhibit~~ show similar patterns, while~~.~~ i~~I~~n

India and Melanesia, the number ~~of described species per paper~~ increased until ca. 2013~~2012 and 2014, respectively~~, followed by a ~~subsequent~~ decline (S8-S11 Fig).

The number of type specimens in a type series has globally decreased from ca. 20 ~~type specimens per species description~~ to fewer than 10 (p-value$_{LM}$ 0.005). A similar pattern is observed in Melanesia (p-value$_{LM}$ 0.003). Ecuador and India ~~do not~~ show ~~any~~ no significant trends~~ in the evolution of the number type specimens~~. In Madagascar, the overall trend is a steady decrease, although some recently described species have ~~significantly~~ reversed that trend (p-value$_{GAM}$ 0.004) (S8-S11 Fig).

The number of field ~~campaigns~~ trips required to collect the entire type series globally shows huge dispersion, with an overall stable trend between 1.5 and 3 field ~~campaigns~~ trips required to collect the type series (S8-S11 Fig).

Discussion

Anuran species descriptions amid a biodiversity crisis

Taxonomists have named more than 2.3 million species [52~~49~~] at a pace of 20,000 species descriptions per year [53~~50~~]. However, this represents only a small fraction of the world's predicted species richness [15~~15~~]. In the past century,~~We are the first generation to~~ we have come to ~~fully comprehend~~realize that ~~we~~ our planet is facing~~are in the midst of~~ a biodiversity crisis~~n extinction event~~, while the majority of species remain undiscovered and undescribed. Unless we reverse the current trend of threats, ~~W~~we may ~~also~~ be among the last generations ~~having~~ with the opportunity to explore and document Earth's biodiversity [39, 54~~37, 51~~].

Here, we evaluated the time required to describe a frog species ~~using either traditional and contemporary methodologies~~with a focus on four megadiverse tropical regions. Despite being the most endangered class of vertebrates [41~~39~~], amphibians are being described at remarkably high rates, and the numerous candidate species flagged worldwide suggest that true amphibian richness —and consequently the number of threatened species— ~~have~~ has been, and remains, ~~highly~~ underestimated [10, 30, 32, 55].

Our results suggest that the mean description time from the collection of the first type specimen to publication is 11.3 years per species, with 4.5 years dedicated to the type series collection and 6.8 years to the description *sensu stricto*. We found similar values across three of the four analyzed regions, with India showing lower values (see Results). Fontaine et al. [~~37~~39] found similar "shelf" figures for tetrapods analyzing a random set of species described in 2007. Our results indicate that, for frogs, ~~these figures are~~the description times are not decreasing at the global level. While continental regions (e.g., India and Ecuador) exhibit a trend of declining description times, insular regions (e.g., Madagascar and Melanesia) show an increase~~Our data suggest a trend of decreasing description times in continental regions (India and Ecuador), while description times are increasing in insular regions (Madagascar and Melanesia)~~. This pattern may be explained by the ~~greater~~ increasing presence and activity of local taxonomists in continental countries, while in the studied insular regions, taxonomy has historically been led by foreign institutions, which are experiencing a reduction in the number of specialists [56~~52~~]. Overall, our estimate of the global pattern is U-shaped, with current description times being longer than those in 2012 but shorter than those in 2000.

Thirty-six percent~~Only 36%~~ of frog species described since 2000 were ~~collected~~ named within~~ a~~ five -years ~~timeframe~~of first collection~~,~~ , ~~. This finding~~ highlighting~~s~~ ~~indicating that a notable proportion of described species were collected recently and highlighting~~ the importance of continued collecting. A~~, as a~~ substantial proportion of recently described species ~~discoveries~~ rely on newly collected material, which underscores the need to maintain active fieldwork, particularly in poorly known areas, at a time when logistical, political, and regulatory barriers to collecting and vouchering are increasing. ~~However~~In contrast, ~~T~~the remaining 64% of species were collected earlier —up to 125 years ago— and described much later~~,~~. This ~~,~~suggests~~ing~~ that new

species are ~~rarely~~ not always ~~identified~~recognized as such in the field, or, if they are, their formal description is not prioritized. It also ~~suggests~~reflects ~~and~~ that targeted sampling ~~campaigns~~ aimed at collecting specific, suspected new species is relatively~~are~~ uncommon. ~~Together, The remaining species were collected earlier — up to 125 years ago — and described much later.~~ ~~t~~~~This underscores~~~~reinforce the need to maintain active fieldwork, particularly in poorly known areas, at a time when logistical, political, and regulatory barriers to collecting and vouchering are increasing.~~ They also support the ~~importance of~~ critical role of public scientific collections as reservoirs of undocumented diversity~~potential new species but~~, while also raising concerns ~~at the same time, it concerns~~ about the lack of resources, time, or interest dedicated to examining the collected material. Public collections contain a wealth of overlooked specimens (e.g., [57–61~~53–57~~]). In fact, as Bebber et al. [62~~58~~] noticed, a significant percentage of the world's undescribed species have already been collected and lodged in museums, but await further scientific investigation and description~~and remain forgotten on museum shelves~~. In the ~~current~~ context of the current biodiversity crisis, some authors have suggested prioritizing the collection and preservation of as many specimens as possible as testimonies of a vanishing biodiversity, ensuring future generations can describe species that may already be extinct [39–63~~37, 59~~]. Collecting ~~specimens~~efforts are ~~is the al and~~ essential ~~of taxonomy and is crucial~~ for addressing the geographical and taxonomic gaps that still exist. To maximize their contribution to biodiversity discovery ~~and conservation~~, ~~such efforts~~collecting should ~~However, unless this strategy is~~~~collecting must~~ be paired with ~~thorough revision and~~ proper specimen processing ~~of all collected specimens~~ (including tissue sampling for genetic analysis) and thorough taxonomic revision ~~. This ensures that collected specimens contribute~~to promptly ~~to the~~~~of collected material~~detection ~~of~~candidate species and prioritize their description~~the development of~~ ~~by providing a more accurate picture of biodiversity~~. ~~, it may result in decades of specimens remaining unexamined on shelves, delaying species descriptions that could contribute to their conservation.~~ To ensure ~~that~~describe as many species as possible ~~are described~~ while they still exist, it is ~~necessary~~ essential to reverse decades of funding cuts to taxonomy. Increased investment would support the creation and maintenance of much-needed taxonomic positions, expand sampling efforts, and enable thorough scrutiny of both recent and historical collections. In parallel, ~~to~~ adopting streamlined species description methodologies (e.g., "fast-track taxonomy" in the sense explained above)~~,~~ ~~the integrati~~and integrating~~on~~ ~~of~~ molecular data to support rapid identifications can improve efficiency of species discovery and descriptions ~~to support rapid identifications, and increasing funding for taxonomic research~~. ~~Molecular screenings should become a standard practice, alongside proper processing of all collected specimens, at least to identify taxa that cannot be assigned to known species, especially after collection campaigns in poorly surveyed regions.~~

Tropical regions harbor most of Earth's terrestrial biodiversity, yet they remain highly threatened and among the least scientifically understood [64–65]. Large-scale molecular screenings are still relatively rare, but those conducted have revealed high numbers (even hundreds) of potential new species—enabled by comparisons with previously sequenced taxa [10, 30, 32, 55]. Despite growing use of molecular data in species descriptions over the last decades, it has yet to become a standard practice (see results; [53]). Our results suggest that incorporating molecular data into frog descriptions significantly increases description time in some regions. While adding any procedure to the species descriptions inherently increases the time required, the increase of time can also be attributed to cryptic species, which may remain undescribed on shelves for years until identified molecularly. Sequencing type specimens during species descriptions offers benefits for taxonomy and biodiversity science. DNA-barcodes linked to identified and re-examinable type specimens establish a reliable connection between genetic data and physical vouchers from the outset [66]. This enables unambiguous specimen assignment to newly described species often without taxonomic expertise—except in cases of introgression, hybridization, or human error [67]. Therefore, by leveraging new methodologies and increasingly affordable molecular tools, the widespread use of DNA barcodes to screen collected material should ideally become a standard practice among taxonomists where feasible, especially in poorly surveyed regions [68].

Genetically characterizing the collected material and publicly releasing the georeferenced sequences would not only highlight candidate species—thereby promoting their formal description—but also provide valuable data for other disciplines such as biogeography, evolutionary biology, and ecology. Such efforts could help reduce the Wallacean shortfall [12] by revealing new localities and improving our understanding of intraspecific diversity (e.g., macrogenetics; [69]). Although the ideal species description involves partial sequencing to allow DNA-based identification, genetic comparison, and phylogenetic contextualization, the use of molecular data is not strictly necessary. We acknowledge that socio-economic constraints and legal restrictions on specimen or tissue export in many countries unfortunately limit the widespread implementation of these molecular methods. ~~Earth's tropical regions remain largely unexplored. Large-scale molecular screenings are still relatively rare, but those conducted have revealed high numbers (even hundreds) of potential new species [10, 30, 60]. Although the use of molecular data in species descriptions has increased in the last decades, it has yet to become a standard practice (see results, [50]). Our results suggest that incorporating molecular data into frog descriptions significantly increases description time in some regions. While adding any procedure to the species descriptions inherently increases the time required, the increase of time can also be attributed to cryptic species, which may remain undescribed on shelves for years until identified molecularly. Therefore, by leveraging new methodologies and increasingly affordable molecular tools, the widespread use of barcodes on collected material should become a common practice among taxonomists [61]. Genetically characterizing the collected material and making the sequences with specific localities publicly available would not only highlight candidate species, thereby promoting their formal description, but also provide valuable data for other disciplines. Subsequent analyses could help reduce the Wallacean shortfall (i.e., limited knowledge about species' geographical distributions) by discovering new localities and improving our understanding of intraspecific diversity (e.g., macrogenetics; [62]).~~

Given the number of species still awaiting discovery and description, it is evident that we need more effective solutions for inventory and protect Earth's amphibian fauna before ~~it~~ many species ~~vanishes~~vanish. Over ~~an~~ the average 11-year span than takes to formally describe a frog species since the first specimen is collected, vast areas of currently suitable habitats are likely to disappear if current rates of habitat destruction continue [70–71~~63–64~~].

Types of species descriptions and future perspectives

A foundational task of taxonomy is comparing the variability of target specimens against a set of related species to assess whether the observed variability justifies considering them as a distinct unit [72~~65~~]. Early species classifications were constrained by the morphological traits available at the time, leading to minimalist and often rudimentary descriptions due to the limited methodologies and understanding [73–74~~21, 66~~]. Today, species descriptions are in general more complex, meticulous, and precise. However, significant disparities exist in their current structure and length. Traditional taxonomic practice follows a comprehensive approach to species descriptions, which, over time, incorporated new methodologies and lines of evidence within an integrative framework. This ~~often~~ sometimes led to excessively detailed and lengthy descriptions ~~that span tens of pages for a single species (e.g., [67])~~. Although concise species descriptions are not new in taxonomy—Linnaeus, Boulenger, and others provided brief yet sufficient accounts, especially when species diversity was less understood and few cryptic taxa were recognized—what is termed here 'fast-track taxonomy' builds on this earlier practice to address the increasingly pressing need to describe species rapidly amid an accelerating biodiversity crisis. This approach does not compromise rigor, since it focuses on essential diagnostic traits, provides concise but accurate descriptions, and incorporates high-quality images to compensate for reduced textual detail.~~In contrast, "fast-track taxonomy" accelerates species description by focusing on essential diagnostic traits, providing concise descriptions while maintaining diagnostic accuracy, and incorporating high-quality images to compensate for reduced textual detail.~~ This results in shorter species accounts, ultimately allowing the description of hundreds of species in a single article (e.g., [37–74~~35, 68~~]). Even more, within the framework of "turbo-taxonomy" [75~~69~~], some authors describe hundreds of species based only on consensus barcode

DNA sequence or molecular diagnostic characters, using these as the principal basis of species delimitation and diagnosis (e.g., [35–76~~33, 70~~]). However, this approach has been widely criticized, as reliance on mitochondrial DNA~~ (mtDNA)~~ or barcodes alone does not provide sufficient evidence for robust species delimitation [77], and it also precludes comparison with already described but unsequenced taxa, increasing the risk of redundant nomenclature~~ [71]~~.

There is an undeniable urgency to describe as many species as possible before they disappear. However, speeding up the process of naming species should not compromise the quality of species hypothese~~i~~s [22–36~~22, 34~~]. Overcoming the Linnean shortfall while ignoring the Wallacean ~~shortfall (~~and other shortfalls~~;~~ (see: [78~~72~~]) makes little sense if the ultimate goal is to conserve species. Identifying species without understanding where they occur or how they persist in their ecosystems limits the effectiveness of conservation efforts. However, must every shortfall be addressed in the initial process of description? Certain standards are clearly necessary to ensure taxonomic descriptions remain~~s~~ consistent, comparable, and facilitate the work of future taxonomists. Although new methods have emerged to disentangle taxonomic challenges derived from poor descriptions or poorly preserved specimens (e.g., [79~~73~~]), we still face the consequences of brief historical descriptions, scattered (or lost) type material, and imprecise type localities, which deter anyone from ~~attempting~~ tackling certain taxonomic decisions [21–73~~21, 66~~]~~)~~.

Although integrative taxonomy has been a good practice of many taxonomists along history, it is in the last decades when taxonomy is truly becoming multidisciplinary, ~~Taxonomy is becoming increasingly multidisciplinary,~~ integrating approaches and techniques from disciplines such as genetics~~(omics)~~, biogeography, bioacoustics, morphometry, and osteology (CT scans). While these approaches may enhance accuracy and provide more information about species, they often require complex techniques and tools that can discourage non-specialists (but see iTaxotools; [80~~74~~]). But how much complexity is necessary for a species description? Must every bone be described, or all biogeographical affinities revealed upfront?

Another challenge in taxonomy is that much of the original biological materials used in species descriptions are often scattered across museum collections worldwide, making it difficult and expensive to revisit and re-study. Additionally, extensive biodiversity data literature exists, comprising different sets of information. While genetic data are now centralized and easy to retrieve (e~~i~~.g~~e~~., GenBank, [81~~75~~]; BOLD, [82~~76~~]), other crucial data produced during species descriptions are either absent from accessible repositories, incomplete, or insufficiently standardized, limiting their reuse [53–54~~50–51~~]. Even when available, these data may be inaccessible to many due to paywalls, such as those in Zootaxa, one of the most popular taxonomic journals. This hinders taxonomists in resource-limited regions from accessing free descriptions to describe vanishing species in hyperdiverse tropical countries.

A previously ~~potential~~ suggested ~~solution~~ strategy to enhance ~~both~~ data accessibility and thereby ~~the speed of~~accelerate species descriptions, without compromising species hypothesis is “wikifying” taxonomic data~~y~~ (i.e., cybertaxonomy [83–86]). This approach integrates traditional taxonomic practices with modern digital platforms. The formal description would continue to follow (unless a better solution is found) established taxonomic procedures (i.e., peer-reviewed publications that comply with the ICZN). However, information about a species or candidate species would be available on a centralized digital platform uploaded by ORCID-identified researchers or through referenced literature. This would draw attention to these undescribed lineages and raise awareness among colleagues working on or possessing information about the same taxa [21, 87–88]. If centralized, this system would liberate taxonomic data from conventional publication formats, make it easy to find all information about a species, and facilitate the continuous updating of species descriptions and data before, during, and after their formal description (e.g., DNA sequences, traits, and distributional data). In addition, it could promote the release of data that, due to their magnitude or nature, are not usually published but are nonetheless of interest (e.g., morphological measurements of already described species). ~~This approach begins after formal taxonomic practices are completed (i.e., peer-reviewed publications~~

that comply with the ICZN), and complements them by integrating the resulting data into modern digital platformsintegrates traditional taxonomic practices with modern digital platforms. This liberates taxonomic data from conventional publication formats and makes it centralized, dynamic, and easily reusable by others. If centralized, it would allow for the dynamic updating and sharing of species descriptions and data, enhancing accessibility and collaboration within the scientific community. It would promote concise, versioned species pages that can be continuously updated with new information (e.g., DNA sequences, traits, and distributional data) improving the efficiency of biodiversity research [21, 77–78]. This strategy would allow the mass description of species following a (to be defined) minimum standard to ensure reliable descriptions, with the possibility of later adding information to improve update them ([21, 5321, 50]; and references therein). Consequently, it would not be necessary to delay naming species to provide the most complete description possible. Species could be included in the conservation scheme from the outset, and although the act of naming itself remains static, their descriptions and biological content could remain dynamic and be updated over time. Leveraging the fact that a small number of journals are responsible for the vast majority of published taxonomic papers, these leading journals—as suggested by Miller et al [86]—could take the lead and coordinate with other journals to request standardized taxonomical data (e.g., morphometric values, variable labelling, coordinates) and routinely upload it to digital platforms. Once a shared minimum standard for reliable descriptions and a standardized data submission format is established, other journals and authors could follow the same templates, ensuring consistency and broad adoption across the field. Ultimately, as in many other fields, AI is expected to play an important role in several aspects of taxonomic practice, most likely contributing to the acceleration of species descriptions. However, discussing the plausible developments of this new tool for taxonomy is beyond the scope of this paper.

Species can be protected through direct conservation efforts by targeting specific species or indirectly by safeguarding their habitats. Under current legislation, only species bearing a scientific name are recognized for conservation. Undescribed species are not assessed by the IUCN and are generally excluded from biological inventories used to prioritize conservation areas. With the current figures of species descriptions, if we must wait until a species is formally named to protect it, we will lose the race against extinction for many of them. In some cases, taxonomists describe species whose existence has been known (to specialists) for decades, but because formal naming can be delayed by multiple external constraints,and when they finally name them, they sadly realize that the locality where it was first collected has been completely destroyed (e.g., [89–9079–80]). This highlights a critical gap in conservation: undescribed species constitute the majority of Earth's biodiversity, but lack formal recognition and, consequently, legal protection, making them more vulnerable to extinction [4038].

Therefore, the biggest challenge faced by undescribed species is that current conservation legislation is generally (describednamed)species-centered. Since descriptions are slow and take increasingly longer, many species do not (and will not) have any form of protection unless they happen to occur within a protected area by chance. A potential solution is to useUsing cybertaxonomy could enableto includinge undescribed species in the conservation scheme from the outset, or alternatively, and/or to enhance a region-centered conservation approach (i.e., indirect conservation). In the latter, we propose accounting for species that have been known for years but remain unnamed—many of which we have substantial data on (e.g., [3735]). These species could be used to prioritize conservation areas based on species "richness", aligning with global targets such as the 30 ×x 30 initiative, which aims to protect 30% of the planet's land and oceans by 2030 [9181]. This approach would prioritize long-term conservation efforts by considering a more realistic diversity inventory. To achieve this, we suggest publishing all available information on undescribed species, such as DNA barcodes and distribution data, to make it available for regional or global studies. We acknowledge that this approach, in some occasions, could inflate the species inventory of a given area. However, the worst outcome would

be conserving divergent conspecific lineages, which, in any case, represent important genetic variability that should be preserved.

Taxonomy crisis

Taxonomy is a foundational science. Almost all biological studies take place downstream ([38, 54~~36, 51~~]). Contradictorily, is an undervalued biological discipline [63~~59~~]. Taxonomic papers receive few citations and are often published in low-impact journals [92–93~~82–83~~] threatening the careers of early-career researchers with low citation indices [94–95~~84–85~~]. Additionally, funding for taxonomy-focused projects is steadily declining [54, 87, 96–97~~51, 77, 86–87~~]. To secure funding and publication in high-impact journals, these projects often need to be framed within cutting-edge methodologies or broader questions. This can result in using a sledgehammer to crack a nut, ultimately extending description time~~-sspans~~.

Whether taxonomists are facing extinction is hotly debated [56, 63, 93, 98–100~~52, 59, 83, 88–90~~]. Some authors argue that the number of authors involved in species descriptions has increased exponentially [56, 101–103~~52, 91–93~~]. Others contend that, despite this author inflation, not all authors listed in taxonomic papers can be considered taxonomists [99, 103~~89,93~~]. On a global scale, there is a regional turnover, with historically leading taxonomic institutions losing taxonomists, while other regions are increasing their numbers [7, 56~~7, 52~~]. Our results confirm that the number of authors involved in frog species descriptions has been steadily increasing. However, we found a globally significant relationship between the increase in the number of authors and a reduction in the description time. These results contradict the presumed undesirable effects of author inflation hypothesis, indicating that task distribution among authors may lead to more efficient species descriptions. However, we did not find this relationship in all regions, suggesting that such improved efficiency may depend on the particularities of each research group.

Our results suggest that, since ca. 2010, the number of published frog taxonomical papers, the number of frog species described each year, and the number of species described per paper have remained stable. This contrasts with the growth in scientific productivity, partially driven by advancements in computational power, new and affordable sequencing technologies, and data analysis software [53, 79–80, 104–106~~50, 73–74, 94–96~~]. Unchanged species diagnosis and description procedures have been flagged as the main cause [21, 53~~21, 50~~]. Moreover, the number of species described per taxonomist has generally declined. While some authors have attributed this trend to a lack of new species to describe [21, 98, 101, 103~~21, 88, 91, 93~~], the large number of candidate species flagged annually and current rates of species descriptions suggest otherwise. This decline may reflect a growing shortage of taxonomic specialists and an increased participation in taxonomy of researchers from other fields [99, 103, 107~~89, 93, 97~~]. This trend may be further exacerbated by the fact that describing a new species, while inherently engaging for most biologists, it is little rewarded. As a result, despite not being taxonomists, some researchers venture into describing one or a few species.

Working in consortiums has been proposed as a strategy to increase the number of species described per article, thus improving the impact metrics of both articles and journals. While this strategy may increase impact metrics, an issue remains: who will be the first author? Is it more advantageous for early-career researchers to be the first author of a less-cited article or to be one among many contributors in a consortium article? Regarding description time, our results suggest that while an increase in the number of authors is associated with shorter description times at a global scale, a higher the number of species described per paper (which is expected to increase the impact metric) has the opposite effect, with more species leading to longer times required to describe each one.

Study caveats

We had to define two subperiods in the species description process and make oversimplified assumptions to analyze data and draw general conclusions. First, the oldest collected specimen in the type series may not represent the species' first scientific encounter, as earlier specimens might

have been overlooked (deliberately or not) or stored elsewhere. Even if it was the first encounter, it might not have been recognized as new in the field. In some cases, collectors are not researchers, and the collected material is often not examined immediately after fieldwork. Second, a species can be described using only the holotype (or even part of it). Since not all specimens in the type series are essential for the species description, the process might not be delayed until their collection. Third, including ancient specimens in the type series of species description was not always strictly necessary, and their inclusion extended the calculated lag time between the first collection and publication. Fourth, the description *sensu stricto* rarely begins immediately after the last type specimen is collected. There are multiple factors that can delay the description of a species for decades. Lastly, we acknowledge that our figures may contain a level of error affecting all species in different degrees, which we did not account for: the time span between manuscript submission and publication. This interval varies depending on the journal and the length of the review process, which can sometimes be lengthy. For the purpose of this study, we have assumed that this interval is part of the species description.

Acknowledgements

We want to acknowledge colleagues who provided fruitful discussions on this issue in the past years, specially Matthijs P. van den Burg and Santiago Castroviejo-Fisher. We are grateful to Ernesto Recuero for his critical reading of the manuscript. We thank the academic editor, Alex Slavenko, as well as Paul Oliver and one anonymous reviewer for their valuable comments and suggestions.

References

1. Lees AC, Pimm SL. Species, extinct before we know them?. Curr Biol. 2015; 25(5): R177–R180.
2. McCallum ML. Vertebrate biodiversity losses point to a sixth mass extinction. Biodiv Cons. 2015; 24(10): 2497–2519.
3. Ceballos G, Ehrlich PR, Dirzo R. Biological annihilation via the ongoing sixth mass extinction signaled by vertebrate population losses and declines. PNAS. 2017; 114(30):E6089-E6096.
4. Ceballos G, Ehrlich PR. The misunderstood sixth mass extinction. Science. 2018; 360(6393): 1080–1081.
5. Young HS, McCauley DJ, Galetti M, Dirzo, R. Patterns, causes, and consequences of anthropocene defaunation. Annu Rev Ecol Evol Syst. 2016; 47: 333–358.
6. Tracewski Ł, Butchart SH, Di Marco M, Ficetola GF, Rondinini C, Symes A, et al. Toward quantification of the impact of 21st-century deforestation on the extinction risk of terrestrial vertebrates. Conserv Biol. 2016; 30(5): 1070–1079.
7. Prathapan KD, Rajan PD. Advancing taxonomy in the global south and completing the grand Linnaean enterprise. Megataxa. 2020; 1(1): 73–77.
8. Button S, Borzée A. A new multi-metric approach for quantifying global biodiscovery and conservation priorities reveals overlooked hotspots for amphibians. [Preprint]. arXiv:2308.08829. 2023 [cited 2024 Dec 12]. Available from: https://arxiv.org/abs/2308.08829.
9. Finn C, Grattarola F, Pincheira-Donoso D. More losers than winners: investigating Anthropocene defaunation through the diversity of population trends. Biol Rev. 2023; 98(5):1732–1748.
10. Carné A, Vieites DR. A race against extinction: The challenge to overcome the Linnean amphibian shortfall in tropical biodiversity hotspots. Divers Distrib. 2024; 30(12): 1–13.
11. Burriel-Carranza B, Tejero-Cicuéndez H, Carné A, Mochales-Riaño G, Talavera A, Al Saadi S, et al. Integrating genomics and biogeography to unravel the origin of a mountain biota: The case of a reptile endemicity hotspot in Arabia. Syst Biol. 2024; syae032.
12. Lomolino MV. Conservation biogeography. In: Lomolino MV, Heaney LR, editors. Frontiers of biogeography: New directions in the geography of nature. Sunderland (MA): Sinauer; 2004. p. 293–296.
13. Brown JH, Lomolino MV. Biogeography. 2nd ed. Sunderland (MA): Sinauer Press; 1998.
14. Mora C, Tittensor DP, Adl S, Simpson AG, Worm B. How many species are there on Earth and in the ocean? PLoS Biol. 2011; 9(8): e1001127.
15. Larsen BB, Miller EC, Rhodes MK, Wiens JJ. Inordinate fondness multiplied and redistributed: the number of species on earth and the new pie of life. Q Rev Biol. 2017; 92(3): 229–265.
16. Mace GM. The role of taxonomy in species conservation. Phil Trans R Soc B. 2004; 359(1444): 711–719.

17. Nori J, Semhan R, Abdala CS, Rojas-Soto O. Filling Linnean shortfalls increases endemicity patterns: conservation and biogeographical implications for the extreme case of *Liolaemus* (Liolaemidae, Squamata) species. Zool J Linn Soc. 2022; 194(2): 592–600.
18. Padial JM, Miralles A, De la Riva I, Vences M. The integrative future of taxonomy. Front Zool. 2010; 7(1): 1–14.
19. Scheffers BR, Joppa LN, Pimm SL, Laurance WF. What we know and don't know about Earth's missing biodiversity. Trends Ecol Evol. 2012; 27: 501–510.
20. Padial JM, Castroviejo-Fisher S, Koehler J, Vila C, Chaparro JC, De la Riva I. Deciphering the products of evolution at the species level: the need for an integrative taxonomy. Zool Scr. 2009; 38(4): 431–447.
21. Riedel A, Sagata K, Suhardjono YR, Tänzler R, Balke M. Integrative taxonomy on the fast track-towards more sustainability in biodiversity research. Front Zool. 2013; 10: 1–9.
22. Vences M. The promise of next-generation taxonomy. Megataxa. 2020; 1(1): 35–38.
23. Meegaskumbura M, Bossuyt F, Pethiyagoda R, Manamendra-Arachchi K, Bahir M, Milinkovitch MC, Schneider CJ. Sri Lanka: an amphibian hot spot. Science. 2002; 298(5592): 379–379.
24. Fouquet A, Gilles A, Vences M, Marty C, Blanc M, Gemmell NJ. Underestimation of species richness in Neotropical frogs revealed by mtDNA analyses. PLoS ONE. 2007; 2(10):e1109.
25. Clare EL, Lim BK, Fenton MB, Hebert PD. Neotropical bats: estimating species diversity with DNA barcodes. PLoS ONE. 2011; 6(7): e22648.
26. Funk WC, Caminer M, Ron SR. High levels of cryptic species diversity uncovered in Amazonian frogs. Proc R Soc B. 2012; 279(1734): 1806–1814.
27. Milá B, Tavares ES, Munoz Saldana A, Karubian J, Smith TB, Baker AJ. A trans-Amazonian screening of mtDNA reveals deep intraspecific divergence in forest birds and suggests a vast underestimation of species diversity. PLoS ONE. 2012; 7(7): e40541.
28. Tänzler R, Sagata K, Surbakti S, Balke M, Riedel A. DNA barcoding for community ecology-how to tackle a hyperdiverse, mostly undescribed Melanesian fauna. PLoS ONE. 2012; 7(1): e28832.
29. Veijalainen A, Wahlberg N, Broad GR, Erwin TL, Longino JT, Sääksjärvi IE. Unprecedented ichneumonid parasitoid wasp diversity in tropical forests. Proc R Soc Ser B Biol. Sci. 2012; 279(1748): 4694–4698.
30. Vacher JP, Chave J, Ficetola FG, Sommeria-Klein G, Tao S, Thébaud C, et al. Large-scale DNA-based survey of frogs in Amazonia suggests a vast underestimation of species richness and endemism. J Biogeogr. 2020; 47(8): 1781–1791.
31. Oliver PM, Bower DS, McDonald PJ, Kraus F, Luedtke J, Neam K, et al. Melanesia holds the world's most diverse and intact insular amphibian fauna. Comm Biol. 2022; 5(1): 1182.
32. Ferreira F, Oliver P, Kraus F, Günther R, Richards S, Tjaturadi B, et al. Molecular and acoustic evidence for large-scale underestimation of frog species diversity on New Guinea. Front Biogeogr. 2025;18:e137988.
33. Chan KO, Hutter CR, Wood Jr PL, Su YC, Brown RM. (2022). Gene flow increases phylogenetic structure and inflates cryptic species estimations: a case study on widespread Philippine puddle frogs (*Occidozyga laevis*). Syst. Biol. 2022; 71(1): 40-57.
34. Cowie RH, Bouchet P, Fontaine B. The Sixth Mass Extinction: fact, fiction or speculation?. Biol Rev. 2022; 97(2): 640–663.
35. Sharkey MJ, Janzen DH, Hallwachs W, Chapman EG, Smith MA, Dapkey T, et al. Minimalist revision and description of 403 new species in 11 subfamilies of Costa Rican braconid parasitoid wasps, including host records for 219 species. ZooKeys. 2021; 1013:1–665.
36. Fernandez-Triana JL. Turbo taxonomy approaches: lessons from the past and recommendations for the future based on the experience with Braconidae (Hymenoptera) parasitoid wasps. ZooKeys. 2022; 1087: 199-220.
37. Rakotoarison A, Scherz M, Glaw F, Köhler J, Andreone F, Franzen M, et al. Describing the smaller majority: integrative taxonomy reveals twenty-six new species of tiny microhylid frogs (genus *Stumpffia*) from Madagascar. Vertebr Zool. 2017; 67: 271–398.
38. Favret C. The 5 'D's of Taxonomy: A User's Guide. Q Rev Biol. 2024; 99(3): 131–156.
39. Fontaine B, Perrard A, Bouchet P. 21 years of shelf life between discovery and description of new species. Curr Biol. 2012; 22(22): R943–R944.
40. Liu J, Slik F, Zheng S, Lindenmayer DB. Undescribed species have higher extinction risk than known species. Conserv Lett. 2022; 15(3): e12876.
41. Luedtke JA, Chanson J, Neam K, Hobin L, Maciel AO, Catenazzi A, et al. Ongoing declines for the world's amphibians in the face of emerging threats. Nature. 2023; 622(7982): 308–314.
42. Stuart SN, Chanson JS, Cox NA, Young BE, Rodrigues AS, Fischman DL, et al. Status and trends of amphibian declines and extinctions worldwide. Science. 2004; 306: 1783–1786.

43. Stuart SN, Hoffmann M, Chanson JS, Cox NA, Berridge RJ, Ramani PP, Young BE, editors. Threatened Amphibians of the World. Barcelona, Spain: Lynx Edicions; Gland, Switzerland: IUCN; Arlington, Virginia, USA: Conservation International; 2008.
44. McDonald PJ, Brown RM, Kraus F, Bowles P, Arifin U, Eliades SJ, et al. Cryptic extinction risk in a western Pacific lizard radiation. Biodivers Conserv. 2022; 31(8): 2045–2062.
45. Frost DR. Amphibian Species of the World: An Online Reference. Version 6.2 [Internet]. 2024 [cited 2024 Dec 12]. Available from: https://amphibiansoftheworld.amnh.org/index.php
46. R Core Team. R: A language and environment for statistical computing. R Foundation for Statistical Computing; 2024. Available from: https://www.R-project.org/.
47. Revelle W. psych: Procedures for Psychological, Psychometric, and Personality Research. R package version 2.4.6. Northwestern University, Evanston, Illinois; 2024. Available from: https://CRAN.R-project.org/package=psych.
48. Wickham H. ggplot2: Elegant Graphics for Data Analysis. Springer-Verlag New York; 2016.
49. Brooks ME, Kristensen K, van Benthem KJ, Magnusson A, Berg CW, Nielsen A, et al. glmmTMB balances speed and flexibility among packages for zero-inflated generalized linear mixed modeling. The R Journal. 2017; 9(2): 378–400.
50. Fox J, Weisberg S. An R companion to applied regression. 3rd ed. Thousan Oaks (CA):Sage; 2019.
51. Hartig F. DHARMa: Residual Diagnostics for Hierarchical (Multi-Level/Mixed) Regression Models [Internet]. R package version 0.4.7. 2024 [cited 2024 Dec 12]. Available from: https://CRAN.R-project.org/package=DHARMa.
52. Bánki O, Roskov Y, Döring M, Ower G, Hernández Robles DR, Plata Corredor CA, et al. Catalogue of Life [Internet]. Version 2024-07-18. Amsterdam (Netherlands): Catalogue of Life; 2024 [cited 2024 Dec 12]. Available from: https://doi.org/10.48580/dgbqz.
53. Miralles A, Bruy T, Wolcott K, Scherz MD, Begerow D, Beszteri B, et al. Repositories for taxonomic data: where we are and what is missing. Syst Biol. 2020; 69(6): 1231–1253.
54. Wheeler QD, Raven PH, Wilson EO. Taxonomy: impediment or expedient?. Science. 2004; 303(5656): 285–285.
55. Jaramillo AF, De la Riva I, Guayasamin JM, Chaparro JC, Gagliardi Urrutia G, Gutiérrez R, et al. Vastly underestimated radiation of Amazonian salamanders (Plethodontidae: Bolitoglossa) and implications about plethodontid diversification. Mol Phylogenet Evol. 2020; 149: 1–23.
56. Costello MJ, May RM, Stork NE. Can we name Earth's species before they go extinct? Science. 2013; 339(6118): 413–416.
57. Sánchez-Vialas A, Calvo-Revuelta M, Castroviejo-Fisher S, De la Riva I. Synopsis of the amphibians of Equatorial Guinea based upon the authors' field work and Spanish natural history collections. Proc Calif Acad Sci. 2020; 66:137–230.
58. Sánchez-Vialas A, Calvo-Revuelta M, De la Riva I. Synopsis of the reptiles of Equatorial Guinea. Zootaxa. 2022; 5202(1):1–197.
59. Sánchez-Vialas A, Copete-Mosquera LA, Calvo-Revuelta M. Contributions to the amphibians and reptiles of Myanmar: insights from the Leonardo Fea legacy housed at the Museo Nacional de Ciencias Naturales of Madrid. Zootaxa. 2024, 5457(1): 1–64.
60. Brown RM, Calvo-Revuelta M, Goyes Vallejos J, Sánchez-Vialas A, De la Riva I. On the second (or, rather, the first) specimen of the recently described *Calliophis salitan* (Squamata: Elapidae), with the first report of the species from Mindanao Island, southern Philippines. Herpetology notes. 2021; 14: 1027–1035.
61. López-Estrada EK, Sánchez-Vialas A, Manzanilla J, Piñango C, Ruiz JL, García-París M. An overview of the taxonomy and geographic distribution of Venezuelan *Epicauta* (Coleoptera: Meloidae). Ann Zool. 2022; 72(1): 9–47.
62. Bebber DP, Carine MA, Wood JR, Wortley AH, Harris DJ, Prance GT, et al. Herbaria are a major frontier for species discovery. PNAS. 2010; 107(51): 22169–22171.
63. Engel MS, Ceríaco LM, Daniel GM, Dellapé PM, Löbl I, Marinov M, et al. The taxonomic impediment: a shortage of taxonomists, not the lack of technical approaches. Zool J Linn Soc. 2021; 193(2): 381–387.
64. Moura MR, Jetz W. Shortfalls and opportunities in terrestrial vertebrate species discovery. Nat Ecol Evol. 2021; 5(5):631–9.
65. Šmíd J. Geographic and taxonomic biases in the vertebrate tree of life. J Biogeogr. 2022;49(12):2120–9.
66. Buckner JC, Sanders RC, Faircloth BC, Chakrabarty P. The critical importance of vouchers in genomics. Elife. 2021;10:e68264.
67. Carné A, Vieites DR, van den Burg MP. In Vouchers We (Hope to) Trust: Unveiling Hidden Errors in GenBank's Tetrapod Taxonomic Foundations. Mol Ecol. 2025; e17812.

68. Padial JM, De la Riva I. Integrative taxonomists should use and produce DNA barcodes. Zootaxa. 2007; 1586: 67–68.
69. Blanchet S, Prunier JG, De Kort H. Time to go bigger: emerging patterns in macrogenetics. Trends Genet. 2017; 33(9): 579–580.
70. Vieilledent G, Grinand C, Rakotomalala FA, Ranaivosoa R, Rakotoarijaona JR, Allnutt TF, et al. Combining global tree cover loss data with historical national forest cover maps to look at six decades of deforestation and forest fragmentation in Madagascar. Biol Conserv. 2018; 222: 189–197.
71. Silva Junior CH, Pessôa AC, Carvalho NS, Reis JB, Anderson LO, Aragão LE. The Brazilian Amazon deforestation rate in 2020 is the greatest of the decade. Nat Ecol Evol. 2021; 5(2): 144–145.
72. Mayr E. Principles of systematic zoology. McGraw-Hill; 1969.
73. Godfray HCJ. Challenges for taxonomy. Nature. 2002; 417(6884): 17–19.
74. Riedel A, Narakusumo RP. One hundred and three new species of *Trigonopterus* weevils from Sulawesi. ZooKeys. 2019; 828: 1–153.
75. Butcher BA, Smith MA, Sharkey MJ, Quicke DL. A turbo-taxonomic study of Thai *Aleiodes* (*Aleiodes*) and *Aleiodes* (*Arcaleiodes*) (Hymenoptera: Braconidae: Rogadinae) based largely on COI barcoded specimens, with rapid descriptions of 179 new species. Zootaxa. 2012; 3457(1): 1–232.
76. Meierotto S, Sharkey MJ, Janzen DH, Hallwachs W, Hebert PD, Chapman EG, et al. A revolutionary protocol to describe understudied hyperdiverse taxa and overcome the taxonomic impediment. Dtsch Entomol. 2019; 66(2): 119–145.
77. Reyes-Velasco J. A revision of recent taxonomic changes to the eyelash palm pitviper, *Bothriechis schlegelii* (Serpentes, Viperidae). Herpetozoa. 2024; 37: 305–318.
78. Hortal J, de Bello F, Diniz-Filho JAF, Lewinsohn TM, Lobo JM, Ladle RJ. Seven shortfalls that beset large-scale knowledge of biodiversity. Annu Rev Ecol Evol. Syst. 2015; 46(1): 523–549.
79. Rancilhac L, Bruy T, Scherz MD, Pereira EA, Preick M, Straube N, et al. Target-enriched DNA sequencing from historical type material enables a partial revision of the Madagascar giant stream frogs (genus *Mantidactylus*). J Nat Hist. 2020; 54(1–4): 87–118.
80. Vences M, Miralles A, Brouillet S, Ducasse J, Fedosov A, Kharchev V, et al. iTaxoTools 0.1: Kickstarting a specimen-based software toolkit for taxonomists. Megataxa. 2021; 6:77–92.
81. Benson DA, Karsch-Mizrachi I, Lipman DJ, Ostell J, Sayers EW. GenBank. Nucleic Acids Res. 2010; 38: 46–51.
82. Ratnasingham S, Hebert PD. BOLD: The Barcode of Life Data System (http://www. barcodinglife. org). Mol Ecol Notes. 2007; 7(3): 355–364.
83. Godfray HC.J, Clark BR, Kitching IJ, Mayo SJ, Scoble MJ. The web and the structure of taxonomy. Syst. Biol. 2007; 56(6): 943–955.
84. Mietchen D, Hagedorn G, Förstner KU, Kubke MF, Koltzenburg C, Hahnel M, Penev L. Wikis in scholarly publishing. Information Services and Use. 2011; 31(1-2): 53–59.
85. Maddison DR, Guralnick R, Hill A, Reysenbach AL, McDade LA. Ramping up biodiversity discovery via online quantum contributions. Trends Ecol Evol. 2012; 27(2): 72–77.
86. Miller J, Dikow T, Agosti D, Sautter G, Catapano T, Penev L, et al.. From taxonomic literature to cybertaxonomic content. BMC biology. 2012; 10(1): 87.
87. Godfray Jr HCJ. Linnaeus in the information age. Nature. 2007; 446(7133): 259–260.
88. Wheeler QD, Knapp S, Stevenson DW, Stevenson J, Blum SD, Boom BM, et al. Mapping the biosphere: exploring species to understand the origin, organization and sustainability of biodiversity. Syst Biodivers. 2012; 10(1): 1–20.
89. Barratt CD, Lawson LP, Bittencourt-Silva GB, Doggart N, Morgan-Brown T, Nagel, P, et al. A new, narrowly distributed, and critically endangered species of spiny-throated reed frog (Anura: Hyperoliidae) from a highly threatened coastal forest reserve in Tanzania. Herpetol J. 2017; 27(1):13-24.
90. Vences M, Köhler J, Scherz MD, Hutter CR, Maheritafika HMR, Rafanoharana JM, et al. Four new species of forest-dwelling mantellid frogs from Madagascar allied to *Gephyromantis moseri*. Spixiana. 2024; 46(2):297-319.
91. Dinerstein E, Vynne C, Sala E, Joshi AR, Fernando S, Lovejoy TE, et al. A global deal for nature: Guiding principles, milestones, and targets. Sci Adv. 2019; 5(4): eaaw2869.
92. Agnarsson I, Kuntner M. Taxonomy in a changing world: seeking solutions for a science in crisis. Syst Biol. 2007; 56(3): 531–539.
93. Drew LW. Are We Losing the Science of Taxonomy?. BioScience. 2011; 61(12): 942–946.
94. Krell FT. Why impact factors don't work for taxonomy. Nature. 2002; 415(6875): 957–957.
95. Walter DE, Winterton S. Keys and the crisis in taxonomy: extinction or reinvention?. Annu Rev Entomol. 2007; 52(1): 193–208.

96. Britz R, Hundsdörfer A, Fritz U. Funding, training, permits—the three big challenges of taxonomy. Megataxa. 2020; 1(1): 49–52.
97. Löbl I, Klausnitzer B, Hartmann M, Krell FT. The silent extinction of species and taxonomists—An appeal to science policymakers and legislators. Diversity. 2023; 15(10):1053.
98. Tancoigne E, Dubois A. Taxonomy: no decline, but inertia. Cladistics. 2013; 29(5): 567–570.
99. De Carvalho MR, Ebach MC, Williams DM, Nihei SS, Trefaut Rodrigues M, Grant T, et al. Does counting species count as taxonomy? On misrepresenting systematics, yet again. Cladistics. 2014; 30(3): 322–329.
100. Bradford-Grieve J. Is there a taxonomic crisis?. New Zealand Science Review. 2016; 73(3–4): 83–86.
101. Joppa LN, Roberts DL, Pimm SL. The population ecology and social behaviour of taxonomists. Trends Ecol Evol. 2011; 26(11): 551–553.
102. Bacher S. Still not enough taxonomists: reply to Joppa et al. Trends Ecol Evol. 2012; 27(2): 65–66.
103. Bebber DP, Wood JR, Barker C, Scotland RW. Author inflation masks global capacity for species discovery in flowering plants. New Phytol. 2014; 201(2):700–706.
104. Wetterstrand K. DNA sequencing costs [Internet]. Available from: www.genome.gov/sequencingcostsdata. [cited 2024 Nov 26].
105. Puillandre N, Brouillet S, Achaz G. ASAP: assemble species by automatic partitioning. Mol Ecol Resour. 2021; 21(2): 609–620.
106. Ramírez JL, Valdivia P, Rosas-Puchuri U, Valdivia NL. SPdel: A pipeline to compare and visualize species delimitation methods for single-locus datasets. Mol Ecol Resour. 2023; 23(8): 1959–1965.
107. Tancoigne E, Ollivier G. Evaluating the progress and needs of taxonomy since the Convention on Biological Diversity: going beyond the rate of species description. Aust Syst Bot. 2017; 30(4): 326–336.
1. Lees AC, Pimm SL. Species, extinct before we know them?. Curr Biol. 2015; 25(5): R177–R180.
2. McCallum ML. Vertebrate biodiversity losses point to a sixth mass extinction. Biodiv Cons. 2015; 24(10): 2497–2519.
3. Ceballos G, Ehrlich PR, Dirzo R. Biological annihilation via the ongoing sixth mass extinction signaled by vertebrate population losses and declines. PNAS. 2017; 114(30):E6089-E6096.
4. Ceballos G, Ehrlich PR. The misunderstood sixth mass extinction. Science. 2018; 360(6393): 1080–1081.
5. Young HS, McCauley DJ, Galetti M, Dirzo, R. Patterns, causes, and consequences of anthropocene defaunation. Annu Rev Ecol Evol Syst. 2016; 47: 333–358.
6. Tracewski Ł, Butchart SH, Di Marco M, Ficetola GF, Rondinini C, Symes A, et al. Toward quantification of the impact of 21st-century deforestation on the extinction risk of terrestrial vertebrates. Conserv Biol. 2016; 30(5): 1070–1079.
7. Prathapan KD, Rajan PD. Advancing taxonomy in the global south and completing the grand Linnaean enterprise. Megataxa. 2020; 1(1): 73–77.
8. Button S, Borzée A. A new multi-metric approach for quantifying global biodiscovery and conservation priorities reveals overlooked hotspots for amphibians. [Preprint]. arXiv:2308.08829. 2023 [cited 2024 Dec 12]. Available from: https://arxiv.org/abs/2308.08829.
9. Finn C, Grattarola F, Pincheira-Donoso D. More losers than winners: investigating Anthropocene defaunation through the diversity of population trends. Biol Rev. 2023; 98(5):1732–1748.
10. Carné A, Vieites DR. A race against extinction: The challenge to overcome the Linnean amphibian shortfall in tropical biodiversity hotspots. Divers Distrib. 2024; 30(12): 1–13.
11. Burriel-Carranza B, Tejero-Cicuéndez H, Carné A, Mochales-Riaño G, Talavera A, Al Saadi S, et al. Integrating genomics and biogeography to unravel the origin of a mountain biota: The case of a reptile endemicity hotspot in Arabia. Syst Biol. 2024; syae032.
12. Lomolino MV. Conservation biogeography. In: Lomolino MV, Heaney LR, editors. Frontiers of biogeography: New directions in the geography of nature. Sunderland (MA): Sinauer; 2004. p. 293.
13. Whittaker RJ, Araújo MB, Jepson P, Ladle RJ, Watson JE, Willis KJ. Conservation biogeography: assessment and prospect. Divers Distrib. 2005; 11(1): 3–23.
14. Mora C, Tittensor DP, Adl S, Simpson AG, Worm B. How many species are there on Earth and in the ocean? PLoS Biol. 2011; 9(8): e1001127.
15. Larsen BB, Miller EC, Rhodes MK, Wiens JJ. Inordinate fondness multiplied and redistributed: the number of species on earth and the new pie of life. Q Rev Biol. 2017; 92(3): 229–265.
16. Mace GM. The role of taxonomy in species conservation. Phil Trans R Soc B. 2004; 359(1444): 711–719.
17. Nori J, Semhan R, Abdala CS, Rojas-Soto O. Filling Linnean shortfalls increases endemicity patterns: conservation and biogeographical implications for the extreme case of *Liolaemus* (Liolaemidae, Squamata) species. Zool J Linn Soc. 2022; 194(2): 592–600.

18. Padial JM, Miralles A, De la Riva I, Vences M. The integrative future of taxonomy. Front Zool. 2010; 7(1): 1–14.
19. Scheffers BR, Joppa LN, Pimm SL, Laurance WF. What we know and don't know about Earth's missing biodiversity. Trends Ecol Evol. 2012; 27: 501–510.
20. Padial JM, Castroviejo-Fisher S, Koehler J, Vila C, Chaparro JC, De la Riva I. Deciphering the products of evolution at the species level: the need for an integrative taxonomy. Zool Scr. 2009; 38(4): 431–447.
21. Riedel A, Sagata K, Suhardjono YR, Tänzler R, Balke M. Integrative taxonomy on the fast track-towards more sustainability in biodiversity research. Front Zool. 2013; 10: 1–9.
22. Vences M. The promise of next-generation taxonomy. Megataxa. 2020; 1(1): 35–38.
23. Meegaskumbura M, Bossuyt F, Pethiyagoda R, Manamendra-Arachchi K, Bahir M, Milinkovitch MC, Schneider CJ. Sri Lanka: an amphibian hot spot. Science. 2002; 298(5592): 379–379.
24. Fouquet A, Gilles A, Vences M, Marty C, Blanc M, Gemmell NJ. Underestimation of species richness in Neotropical frogs revealed by mtDNA analyses. PLoS ONE. 2007; 2(10):e1109.
25. Clare EL, Lim BK, Fenton MB, Hebert PD. Neotropical bats: estimating species diversity with DNA barcodes. PLoS ONE. 2011; 6(7): e22648.
26. Funk WC, Caminer M, Ron SR. High levels of cryptic species diversity uncovered in Amazonian frogs. Proc R Soc B. 2012; 279(1734): 1806–1814.
27. Milá B, Tavares ES, Munoz Saldana A, Karubian J, Smith TB, Baker AJ. A trans-Amazonian screening of mtDNA reveals deep intraspecific divergence in forest birds and suggests a vast underestimation of species diversity. PLoS ONE. 2012; 7(7): e40541.
28. Tänzler R, Sagata K, Surbakti S, Balke M, Riedel A. DNA barcoding for community ecology-how to tackle a hyperdiverse, mostly undescribed Melanesian fauna. PLoS ONE. 2012; 7(1): e28832.
29. Veijalainen A, Wahlberg N, Broad GR, Erwin TL, Longino JT, Sääksjärvi IE. Unprecedented ichneumonid parasitoid wasp diversity in tropical forests. Proc R Soc Ser B Biol. Sci. 2012; 279(1748): 4694–4698.
30. Vacher JP, Chave J, Ficetola FG, Sommeria-Klein G, Tao S, Thébaud C, et al. Large-scale DNA-based survey of frogs in Amazonia suggests a vast underestimation of species richness and endemism. J Biogeogr. 2020; 47(8): 1781–1791.
31. Oliver PM, Bower DS, McDonald PJ, Kraus F, Luedtke J, Neam K, et al. Melanesia holds the world's most diverse and intact insular amphibian fauna. Comm Biol. 2022; 5(1): 1182.
32. Cowie RH, Bouchet P, Fontaine B. The Sixth Mass Extinction: fact, fiction or speculation?. Biol Rev. 2022; 97(2): 640–663.
33. Sharkey MJ, Janzen DH, Hallwachs W, Chapman EG, Smith MA, Dapkey T, et al. Minimalist revision and description of 403 new species in 11 subfamilies of Costa Rican braconid parasitoid wasps, including host records for 219 species. ZooKeys. 2021; 1013:1–665.
34. Fernandez-Triana JL. Turbo taxonomy approaches: lessons from the past and recommendations for the future based on the experience with Braconidae (Hymenoptera) parasitoid wasps. ZooKeys. 2022; 1087: 199-220.
35. Rakotoarison A, Scherz M, Glaw F, Köhler J, Andreone F, Franzen M, et al. Describing the smaller majority: integrative taxonomy reveals twenty-six new species of tiny microhylid frogs (genus *Stumpffia*) from Madagascar. Vertebr Zool. 2017; 67: 271–398.
36. Favret C. The 5 'D's of Taxonomy: A User's Guide. Q Rev Biol. 2024; 99(3): 131–156.
37. Fontaine B, Perrard A, Bouchet P. 21 years of shelf life between discovery and description of new species. Curr Biol. 2012; 22(22): R943–R944.
38. Liu J, Slik F, Zheng S, Lindenmayer DB. Undescribed species have higher extinction risk than known species. Conserv Lett. 2022; 15(3): e12876.
39. Luedtke JA, Chanson J, Neam K, Hobin L, Maciel AO, Catenazzi A, et al. Ongoing declines for the world's amphibians in the face of emerging threats. Nature. 2023; 622(7982): 308–314.
40. Stuart SN, Chanson JS, Cox NA, Young BE, Rodrigues AS, Fischman DL, et al. Status and trends of amphibian declines and extinctions worldwide. Science. 2004; 306: 1783–1786.
41. Stuart SN, Hoffmann M, Chanson JS, Cox NA, Berridge RJ, Ramani PP, Young BE, editors. Threatened Amphibians of the World. Barcelona, Spain: Lynx Edicions; Gland, Switzerland: IUCN; Arlington, Virginia, USA: Conservation International; 2008.
42. Frost DR. Amphibian Species of the World: An Online Reference. Version 6.2 [Internet]. 2024 [cited 2024 Dec 12]. Available from: https://amphibiansoftheworld.amnh.org/index.php
43. R Core Team. R: A language and environment for statistical computing. R Foundation for Statistical Computing; 2024. Available from: https://www.R-project.org/.

44. Revelle W. psych: Procedures for Psychological, Psychometric, and Personality Research. R package version 2.4.6. Northwestern University, Evanston, Illinois; 2024. Available from: https://CRAN.R-project.org/package=psych.
45. Wickham H. ggplot2: Elegant Graphics for Data Analysis. Springer-Verlag New York; 2016.
46. Brooks ME, Kristensen K, van Benthem KJ, Magnusson A, Berg CW, Nielsen A, et al. glmmTMB balances speed and flexibility among packages for zero-inflated generalized linear mixed modeling. The R Journal. 2017; 9(2): 378–400.
47. Fox J, Weisberg S. An R companion to applied regression. 3rd ed. Thousan Oaks (CA):Sage; 2019.
48. Hartig F. DHARMa: Residual Diagnostics for Hierarchical (Multi-Level/Mixed) Regression Models [Internet]. R package version 0.4.7. 2024 [cited 2024 Dec 12]. Available from: https://CRAN.R-project.org/package=DHARMa.
49. Bánki O, Roskov Y, Döring M, Ower G, Hernández Robles DR, Plata Corredor CA, et al. Catalogue of Life [Internet]. Version 2024-07-18. Amsterdam (Netherlands): Catalogue of Life; 2024 [cited 2024 Dec 12]. Available from: https://doi.org/10.48580/dgbqz.
50. Miralles A, Bruy T, Wolcott K, Scherz MD, Begerow D, Beszteri B, et al. Repositories for taxonomic data: where we are and what is missing. Syst Biol. 2020; 69(6): 1231–1253.
51. Wheeler QD, Raven PH, Wilson EO. Taxonomy: impediment or expedient?. Science. 2004; 303(5656): 285–285.
52. Costello MJ, May RM, Stork NE. Can we name Earth's species before they go extinct? Science. 2013; 339(6118): 413–416.
53. Sánchez-Vialas A, Calvo-Revuelta M, Castroviejo-Fisher S, De la Riva I. Synopsis of the amphibians of Equatorial Guinea based upon the authors' field work and Spanish natural history collections. Proc Calif Acad Sci. 2020; 66:137–230.
54. Sánchez-Vialas A, Calvo-Revuelta M, De la Riva I. Synopsis of the reptiles of Equatorial Guinea. Zootaxa. 2022; 5202(1):1–197.
55. Sánchez-Vialas A, Copete-Mosquera LA, Calvo-Revuelta M. Contributions to the amphibians and reptiles of Myanmar: insights from the Leonardo Fea legacy housed at the Museo Nacional de Ciencias Naturales of Madrid. Zootaxa. 2024, 5457(1): 1–64.
56. Brown RM, Calvo-Revuelta M, Goyes Vallejos J, Sánchez-Vialas A, De la Riva I. On the second (or, rather, the first) specimen of the recently described *Calliophis salitan* (Squamata: Elapidae), with the first report of the species from Mindanao Island, southern Philippines. Herpetology notes. 2021; 14: 1027–1035.
57. López-Estrada EK, Sánchez-Vialas A, Manzanilla J, Piñango C, Ruiz JL, García-París M. An overview of the taxonomy and geographic distribution of Venezuelan *Epicauta* (Coleoptera: Meloidae). Ann Zool. 2022; 72(1): 9–47.
58. Bebber DP, Carine MA, Wood JR, Wortley AH, Harris DJ, Prance GT, et al. Herbaria are a major frontier for species discovery. PNAS. 2010; 107(51): 22169–22171.
59. Engel MS, Ceríaco LM, Daniel GM, Dellapé PM, Löbl I, Marinov M, et al. The taxonomic impediment: a shortage of taxonomists, not the lack of technical approaches. Zool J Linn Soc. 2021; 193(2): 381–387.
60. Jaramillo AF, De la Riva I, Guayasamin JM, Chaparro JC, Gagliardi-Urrutia G, Gutiérrez R, et al. Vastly underestimated radiation of Amazonian salamanders (Plethodontidae: *Bolitoglossa*) and implications about plethodontid diversification. Mol Phylogenet Evol. 2020; 149: 1–23.
61. Padial JM, De la Riva I. Integrative taxonomists should use and produce DNA barcodes. Zootaxa. 2007; 1586: 67–68.
62. Blanchet S, Prunier JG, De Kort H. Time to go bigger: emerging patterns in macrogenetics. Trends Genet. 2017; 33(9): 579–580.
63. Vieilledent G, Grinand C, Rakotomalala FA, Ranaivosoa R, Rakotoarijaona JR, Allnutt TF, et al. Combining global tree cover loss data with historical national forest cover maps to look at six decades of deforestation and forest fragmentation in Madagascar. Biol Conserv. 2018; 222: 189–197.
64. Silva Junior CH, Pessôa AC, Carvalho NS, Reis JB, Anderson LO, Aragão LE. The Brazilian Amazon deforestation rate in 2020 is the greatest of the decade. Nat Ecol Evol. 2021; 5(2): 144–145.
65. Mayr E. Principles of systematic zoology. McGraw-Hill; 1969.
66. Godfray HCJ. Challenges for taxonomy. Nature. 2002; 417(6884): 17–19.
67. Scherz MD, Daza JD, Köhler J, Vences M, Glaw F. Off the scale: a new species of fish-scale gecko (Squamata: Gekkonidae: *Geckolepis*) with exceptionally large scales. PeerJ. 2017; 5: e2955.
68. Riedel A, Narakusumo RP. One hundred and three new species of *Trigonopterus* weevils from Sulawesi. ZooKeys. 2019; 828: 1–153.

69. Butcher BA, Smith MA, Sharkey MJ, Quicke DL. A turbo-taxonomic study of Thai *Aleiodes* (*Aleiodes*) and *Aleiodes* (*Arcaleiodes*) (Hymenoptera: Braconidae: Rogadinae) based largely on COI barcoded specimens, with rapid descriptions of 179 new species. Zootaxa. 2012; 3457(1): 1–232.
70. Meierotto S, Sharkey MJ, Janzen DH, Hallwachs W, Hebert PD, Chapman EG, et al. A revolutionary protocol to describe understudied hyperdiverse taxa and overcome the taxonomic impediment. Dtsch Entomol. 2019; 66(2): 119–145.
71. Reyes-Velasco J. A revision of recent taxonomic changes to the eyelash palm pitviper, *Bothriechis schlegelii* (Serpentes, Viperidae). Herpetozoa. 2024; 37: 305–318.
72. Hortal J, de Bello F, Diniz-Filho JAF, Lewinsohn TM, Lobo JM, Ladle RJ. Seven shortfalls that beset large-scale knowledge of biodiversity. Annu Rev Ecol Evol. Syst. 2015; 46(1): 523–549.
73. Rancilhac L, Bruy T, Scherz MD, Pereira EA, Preick M, Straube N, et al. Target-enriched DNA sequencing from historical type material enables a partial revision of the Madagascar giant stream frogs (genus *Mantidactylus*). J Nat Hist. 2020; 54(1–4): 87–118.
74. Vences M, Miralles A, Brouillet S, Ducasse J, Fedosov A, Kharchev V, et al. iTaxoTools 0.1: Kickstarting a specimen-based software toolkit for taxonomists. Megataxa. 2021; 6:77–92.
75. Benson DA, Karsch-Mizrachi I, Lipman DJ, Ostell J, Sayers EW. GenBank. Nucleic Acids Res. 2010; 38: 46–51.
76. Ratnasingham S, Hebert PD. BOLD: The Barcode of Life Data System (http://www. barcodinglife. org). Mol Ecol Notes. 2007; 7(3): 355–364.
77. Godfray Jr HCJ. Linnaeus in the information age. Nature. 2007; 446(7133): 259–260.
78. Wheeler QD, Knapp S, Stevenson DW, Stevenson J, Blum SD, Boom BM, et al. Mapping the biosphere: exploring species to understand the origin, organization and sustainability of biodiversity. Syst Biodivers. 2012; 10(1): 1–20.
79. Barratt CD, Lawson LP, Bittencourt-Silva GB, Doggart N, Morgan-Brown T, Nagel, P, et al. A new, narrowly distributed, and critically endangered species of spiny-throated reed frog (Anura: Hyperoliidae) from a highly threatened coastal forest reserve in Tanzania. Herpetol J. 2017; 27(1):13–24.
80. Vences M, Köhler J, Scherz MD, Hutter CR, Maheritafika HMR, Rafanoharana JM, et al. Four new species of forest-dwelling mantellid frogs from Madagascar allied to *Gephyromantis moseri*. Spixiana. 2024; 46(2):297–319.
81. Dinerstein E, Vynne C, Sala E, Joshi AR, Fernando S, Lovejoy TE, et al. A global deal for nature: Guiding principles, milestones, and targets. Sci Adv. 2019; 5(4): eaaw2869.
82. Agnarsson I, Kuntner M. Taxonomy in a changing world: seeking solutions for a science in crisis. Syst Biol. 2007; 56(3): 531–539.
83. Drew LW. Are We Losing the Science of Taxonomy?. BioScience. 2011; 61(12): 942–946.
84. Krell FT. Why impact factors don't work for taxonomy. Nature. 2002; 415(6875): 957–957.
85. Walter DE, Winterton S. Keys and the crisis in taxonomy: extinction or reinvention?. Annu Rev Entomol. 2007; 52(1): 193–208.
86. Britz R, Hundsdörfer A, Fritz U. Funding, training, permits – the three big challenges of taxonomy. Megataxa. 2020; 1(1): 49–52.
87. Löbl I, Klausnitzer B, Hartmann M, Krell FT. The silent extinction of species and taxonomists – An appeal to science policymakers and legislators. Diversity. 2023; 15(10):1053.
88. Tancoigne E, Dubois A. Taxonomy: no decline, but inertia. Cladistics. 2013; 29(5): 567–570.
89. De Carvalho MR, Ebach MC, Williams DM, Nihei SS, Trefaut Rodrigues M, Grant T, et al. Does counting species count as taxonomy? On misrepresenting systematics, yet again. Cladistics. 2014; 30(3): 322–329.
90. Bradford-Grieve J. Is there a taxonomic crisis?. New Zealand Science Review. 2016; 73(3–4): 83–86.
91. Joppa LN, Roberts DL, Pimm SL. The population ecology and social behaviour of taxonomists. Trends Ecol Evol. 2011; 26(11): 551–553.
92. Bacher S. Still not enough taxonomists: reply to Joppa et al. Trends Ecol Evol. 2012; 27(2): 65–66.
93. Bebber DP, Wood JR, Barker C, Scotland RW. Author inflation masks global capacity for species discovery in flowering plants. New Phytol. 2014; 201(2):700–706.
94. W etterstrand K. DNA sequencing costs [Internet]. Available from: www.genome.gov/sequencingcostsdata. [cited 2024 Nov 26].
95. Puillandre N, Brouillet S, Achaz G. ASAP: assemble species by automatic partitioning. Mol Ecol Resour. 2021; 21(2): 609–620.
96. Ramírez JL, Valdivia P, Rosas-Puchuri U, Valdivia NL. SPdel: A pipeline to compare and visualize species delimitation methods for single-locus datasets. Mol Ecol Resour. 2023; 23(8): 1959–1965.

Tancoigne E, Ollivier G. Evaluating the progress and needs of taxonomy since the Convention on Biological Diversity: going beyond the rate of species description. Aust Syst Bot. 2017; 30(4): 326–336.

~~McDonald PJ, Brown RM, Kraus F, Bowles P, Arifin U, Eliades SJ, et al. Cryptic extinction risk in a western Pacific lizard radiation. Biodivers Conserv. 2022; 31(8): 2045–2062.~~

Supporting information

S1 Fig. Heatmap of global specimen collections. Heatmap showing the distribution of specimen collection events in the four analyzed regions over time. The x-axis represents the months of the year, while the y-axis represents the years of collection. The color intensity indicates the number of specimens collected in each time period.

S2 Fig. Heatmap of specimen collections in Madagascar. Heatmap showing the distribution of specimen collection events in Madagascar over time. The x-axis represents the months of the year, while the y-axis represents the years of collection. The color intensity indicates the number of specimens collected in each time period.

S3 Fig. Heatmap of specimen collections in India. Heatmap showing the distribution of specimen collection events in India over time. The x-axis represents the months of the year, while the y-axis represents the years of collection. The color intensity indicates the number of specimens collected in each time period.

S4 Fig. Heatmap of specimen collections in Melanesia. Heatmap showing the distribution of specimen collection events in Melanesia over time. The x-axis represents the months of the year, while the y-axis represents the years of collection. The color intensity indicates the number of specimens collected in each time period.

S5 Fig. Heatmap of specimen collections in Ecuador. Heatmap showing the distribution of specimen collection events in Ecuador over time. The x-axis represents the months of the year, while the y-axis represents the years of collection. The color intensity indicates the number of specimens collected in each time period.

S6 Fig. Residual diagnostics from the DHARMa R package for the fitted models.

S7 Fig. Significant effect plots of variables on description *sensu stricto*. Colored boxes differentiate the regions. Gray= global, Purple = Ecuador, Orange= Madagascar, Green = India, and Blue = Melanesia.

S8 Fig. Temporal evolution of variables in Ecuador. (**a**) Overall description process (black), collection (blue), and description *sensu stricto* (green), (**b**) Nº of authors in each species description (mean), (**c**) Number of published taxonomic papers (count), (**d**) Taxonomic papers that include genetics (percentage), (**e**) Nº of species described each year (count), (**f**) Nº of species described in each paper (mean), (**g**) Nº of type specimens in the type series (mean), (**h**) Nº of field ~~campaigns~~ trips conducted to collect the entire type series (mean).

S9 Fig. Temporal evolution of variables in India. (**a**) Overall description process (black), collection (blue), and description *sensu stricto* (green), (**b**) Nº of authors in each species description (mean), (**c**) Number of published taxonomic papers (count), (**d**) Taxonomic papers that include genetics (percentage), (**e**) Nº of species described each year (count), (**f**) Nº of species described in each paper (mean), (**g**) Nº of type specimens in the type series (mean), (**h**) Nº of field ~~campaigns~~ trips conducted to collect the entire type series (mean).

S10 Fig. Temporal evolution of variables in Madagascar. (**a**) Overall description process (black), collection (blue), and description *sensu stricto* (green), (**b**) Nº of authors in each species description (mean), (**c**) Number of published taxonomic papers (count), (**d**) Taxonomic papers that include genetics (percentage), (**e**) Nº of species described each year (count), (**f**) Nº of species described in each paper (mean), (**g**) Nº of type specimens in the type series (mean), (**h**) Nº of field ~~campaigns~~ trips conducted to collect the entire type series (mean).

S11 Fig. Temporal evolution of variables in Melanesia. (**a**) Overall description process (black), collection (blue), and description *sensu stricto* (green), (**b**) Nº of authors in each species description (mean), (**c**) Number of published taxonomic papers (count), (**d**) Taxonomic papers that include genetics (percentage), (**e**) Nº of species described each year (count), (**f**) Nº of species described in each paper (mean), (**g**) Nº of type specimens in the type series (mean), (**h**) Nº of field ~~campaigns~~ trips conducted to collect the entire type series (mean).

S1 Table. Collection and publication dates for each species and region extracted from the literature.

S2 Table. Lag times of collection (MEX_MIN_YEAR_COLPUB), description *sensu stricto* (MIN_YEAR_COLPUB) and overall process (MAX_YEAR_COLPUB) for each species and region. Categorical and numerical variables used in models are also included.

S3 Table. Statistical comparison of AIC values between empiric (fixed + random variables) and null (random variables only) models.

S4 Table. p-values from the GLMM models and evaluation metrics for global and regional analyses.

MS #PONE-D-25-19328 *Croaking for haste: How long does it take to describe a frog species since its discovery?*

Authors’ replies to the comments of the Editor and Reviewers

We thank the editor and reviewers for their positive and constructive comments. We have followed almost all of these. We reply to each comment in the following pages and explain how we dealt with them during the manuscript revision. Please, find below our responses (in black) to the editor's and reviewers' comments (in blue). Each response is preceded by "---Response" for easy identification. All changes in the newly uploaded manuscript have been highlighted using track changes. We hope that the manuscript is now suitable for publication in PLOS ONE.

PLOS ONE

Dear Dr. De la Riva,

Thank you for submitting your manuscript to PLOS ONE. After careful consideration, we feel that it has merit but does not fully meet PLOS ONE’s publication criteria as it currently stands. Therefore, we invite you to submit a revised version of the manuscript that addresses the points raised during the review process.

Please submit your revised manuscript by Aug 11 2025 11:59PM. If you will need more time than this to complete your revisions, please reply to this message or contact the journal office at plosone@plos.org. When you're ready to submit your revision, log on to https://www.editorialmanager.com/pone/ and select the 'Submissions Needing Revision' folder to locate your manuscript file. Please include the following items when submitting your revised manuscript: A rebuttal letter that responds to each point raised by the academic editor and reviewer(s). You should upload this letter as a separate file labeled 'Response to Reviewers'. A marked-up copy of your manuscript that highlights changes made to the original version. You should upload this as a separate file labeled 'Revised Manuscript with Track Changes'. An unmarked version of your revised paper without tracked changes. You should upload this as a separate file labeled 'Manuscript'.

If you would like to make changes to your financial disclosure, please include your updated statement in your cover letter. Guidelines for resubmitting your figure files are available below the reviewer comments at the end of this letter. If applicable, we recommend that you deposit your laboratory protocols in protocols.io to enhance the reproducibility of your results. Protocols.io assigns your protocol its own identifier (DOI) so that it can be cited independently in the future. For instructions see: https://journals.plos.org/plosone/s/submission-guidelines#loc-laboratory-protocols. Additionally, PLOS ONE offers an option for publishing peer-reviewed Lab Protocol articles, which describe protocols hosted on protocols.io. Read more information on sharing protocols at https://plos.org/protocols?utm_medium=editorial-email&utm_source=authorletters&utm_campaign=protocols.

We look forward to receiving your revised manuscript.

Kind regards,

Alex Slavenko

Academic Editor

PLOS ONE

Journal Requirements:

1. When submitting your revision, we need you to address these additional requirements.

Please ensure that your manuscript meets PLOS ONE's style requirements, including those for file naming. The PLOS ONE style templates can be found at

https://journals.plos.org/plosone/s/file?id=wjVg/PLOSOne_formatting_sample_main_body.pdf and

https://journals.plos.org/plosone/s/file?id=ba62/PLOSOne_formatting_sample_title_authors_affiliations.pdf

---Response: We have checked and our files meet PLOS ONE’S style requirements.

2. Thank you for stating the following financial disclosure: AC was funded by MCIN/AEI/10.13039/501100011033, contract for industrial doctorates aid DIN2021-011964.

Please state what role the funders took in the study. If the funders had no role, please state: "The funders had no role in study design, data collection and analysis, decision to publish, or preparation of the manuscript." If this statement is not correct you must amend it as needed. Please include this amended Role of Funder statement in your cover letter; we will change the online submission form on your behalf.

---Response: We have added the suggested information and included it at the end of the cover letter.

--- Before it read: AC was funded by MCIN/AEI/10.13039/501100011033, contract for industrial doctorates aid DIN2021-011964.

--- Now it reads: AC was funded by MCIN/AEI/10.13039/501100011033, contract for industrial doctorates aid DIN2021-011964. The funders had no role in study design, data collection and analysis, decision to publish, or preparation of the manuscript.

3. When completing the data availability statement of the submission form, you indicated that you will make your data available on acceptance. We strongly recommend all authors decide on a data sharing plan before acceptance, as the process can be lengthy and hold up publication timelines. Please note that, though access restrictions are acceptable now, your entire data will need to be made freely accessible if your manuscript is accepted for publication. This policy applies to all data except where public deposition would breach compliance with the protocol approved by your research ethics board. If you are unable to adhere to our open data policy, please kindly revise your statement to explain your reasoning and we will seek the editor's input on an exemption. Please be assured that, once you have provided your new statement, the assessment of your exemption will not hold up the peer review process.

---Response: Perhaps we selected this option in error. All data were already included in the initial submission and were uploaded as supplementary files.

4. We note that Figure 1a in your submission contain map images which may be copyrighted. All PLOS content is published under the Creative Commons Attribution License (CC BY 4.0), which means that the manuscript, images, and Supporting Information files will be freely available online, and any third party is permitted to access, download, copy, distribute, and use these materials in any way, even commercially, with proper attribution. For these reasons, we cannot publish previously copyrighted maps or satellite images created using proprietary data, such as Google software (Google Maps, Street View, and Earth). For more information, see our copyright guidelines: http://journals.plos.org/plosone/s/licenses-and-copyright.

We require you to either present written permission from the copyright holder to publish these figures specifically under the CC BY 4.0 license, or remove the figures from your submission:

a. You may seek permission from the original copyright holder of Figure 1a to publish the content specifically under the CC BY 4.0 license.

We recommend that you contact the original copyright holder with the Content Permission Form (http://journals.plos.org/plosone/s/file?id=7c09/content-permission-form.pdf) and the following text:

“I request permission for the open-access journal PLOS ONE to publish XXX under the Creative Commons Attribution License (CCAL) CC BY 4.0 (http://creativecommons.org/licenses/by/4.0/). Please be aware that this license allows unrestricted use and distribution, even commercially, by third parties. Please reply and provide explicit written permission to publish XXX under a CC BY license and complete the attached form.”

Please upload the completed Content Permission Form or other proof of granted permissions as an "Other" file with your submission.

In the figure caption of the copyrighted figure, please include the following text: “Reprinted from [ref] under a CC BY license, with permission from [name of publisher], original copyright [original copyright year].”

b. If you are unable to obtain permission from the original copyright holder to publish these figures under the CC BY 4.0 license or if the copyright holder’s requirements are incompatible with the CC BY 4.0 license, please either i) remove the figure or ii) supply a replacement figure that complies with the CC BY 4.0 license. Please check copyright information on all replacement figures and update the figure caption with source information. If applicable, please specify in the figure caption text when a figure is similar but not identical to the original image and is therefore for illustrative purposes only.

The following resources for replacing copyrighted map figures may be helpful:

USGS National Map Viewer (public domain): http://viewer.nationalmap.gov/viewer/

The Gateway to Astronaut Photography of Earth (public domain): http://eol.jsc.nasa.gov/sseop/clickmap/

Maps at the CIA (public domain): https://www.cia.gov/library/publications/the-world-factbook/index.html and https://www.cia.gov/library/publications/cia-maps-publications/index.html

NASA Earth Observatory (public domain): http://earthobservatory.nasa.gov/

Landsat: http://landsat.visibleearth.nasa.gov/

USGS EROS (Earth Resources Observatory and Science (EROS) Center) (public domain): http://eros.usgs.gov/#

Natural Earth (public domain): http://www.naturalearthdata.com/

---Response: The data we used to create the map comes from (https://public.opendatasoft.com/explore/dataset/world-administrative-boundaries/information/) and is licensed under the Open Government Licence v3.0, which is compatible with the Creative Commons Attribution License (CC BY 4.0). We have included this information in the figure caption.

--- Before it read: Time lags and trends in anuran amphibian species descriptions. (a) Cumulative number of frog species described over time. (b) Time lags in the description process. (c) Average description speed per year. Green: description sensu stricto, blue: collection, gray: overall process.

--- Now it reads: Time lags and trends in anuran amphibian species descriptions. (a) Cumulative number of frog species described over time. (b) Time lags in the description process. (c) Average description speed per year. Green: description sensu stricto, blue: collection, gray: overall process. The world map showing country boundaries was obtained from OpenDataSoft (https://public.opendatasoft.com/explore/dataset/world-administrative-boundaries/information/) and is licensed under the Open Government Licence v3.0, which is compatible with the Creative Commons Attribution License (CC BY 4.0).

Additional Editor Comments:

I have now received two reviews for this manuscript. I agree with both reviewers that this manuscript presents an interesting analysis of an impressive dataset, and has some solid results and discussions on a very timely research matter. However, I also share some of reviewer 2's concerns. Some of these are mostly editorial (i.e., the manuscript is quite long and overly detailed in some cases and can be streamlined, as per the excellent suggestion of reviewer 2) and some are related to the content. I agree that some statements made are overly broad and sweeping, and the language can be toned down at times. I also personally have some concerns about advocating fast-track taxonomy and wikification as solutions to the issue. I agree with reviewer 2, also based on my personal experience, that in many cases doing taxonomy properly just takes a whole lot of time, and without investing more resources into there's not much that can be done to speed it up.

---Response: We acknowledge that the manuscript in its original form was quite long and detailed. Following Reviewer #2's suggestions, we have reduced the length of some sections by focusing on the general patterns, which has shortened the manuscript. Additionally, following the editor's and Reviewer #2's suggestions, we have toned down the language throughout the manuscript. Finally, we have placed greater emphasis on the need for increased economic resources for taxonomy.

Overall, I think this is a solid manuscript with a lot of potential, and I encourage the authors to read and adress the reviewer comments carefully if they wish to submit a revised version.

Reviewers' comments:

Reviewer's Responses to Questions

Comments to the Author

1. Is the manuscript technically sound, and do the data support the conclusions?

The manuscript must describe a technically sound piece of scientific research with data that supports the conclusions. Experiments must have been conducted rigorously, with appropriate controls, replication, and sample sizes. The conclusions must be drawn appropriately based on the data presented.

Reviewer #1: Yes

Reviewer #2: Partly

2. Has the statistical analysis been performed appropriately and rigorously?

Reviewer #1: I Don't Know

Reviewer #2: I Don't Know

3. Have the authors made all data underlying the findings in their manuscript fully available?

The PLOS Data policy requires authors to make all data underlying the findings described in their manuscript fully available without restriction, with rare exception (please refer to the Data Availability Statement in the manuscript PDF file). The data should be provided as part of the manuscript or its supporting information, or deposited to a public repository. For example, in addition to summary statistics, the data points behind means, medians and variance measures should be available. If there are restrictions on publicly sharing data—e.g. participant privacy or use of data from a third party—those must be specified.

Reviewer #1: Yes

Reviewer #2: Yes

4. Is the manuscript presented in an intelligible fashion and written in standard English?

PLOS ONE does not copyedit accepted manuscripts, so the language in submitted articles must be clear, correct, and unambiguous. Any typographical or grammatical errors should be corrected at revision, so please note any specific errors here.

Reviewer #1: Yes

Reviewer #2: Yes

5. Review Comments to the Author

Please use the space provided to explain your answers to the questions above. You may also include additional comments for the author, including concerns about dual publication, research ethics, or publication ethics. (Please upload your review as an attachment if it exceeds 20,000 characters)

Reviewer #1: General Assessment:

This manuscript presents a well-conceived and original study based on a remarkable dataset. To the best of my knowledge, it is the first work to quantitatively assess the full duration of the taxonomic description

process—from the initial collection of specimens to the eventual publication of species descriptions. The research addresses an important and timely issue in biodiversity science and has the potential to make a significant contribution to the field.

The authors' approach to categorizing and formalizing their dataset is methodologically rigorous, transparent, and well justified. Their assumptions are clearly detailed and appear reasonable and reproducible. However, as I do not have expertise in statistics, I am not in a position to assess the appropriateness or robustness of the statistical tools and models employed in the analysis.

The discussion is pertinent and well articulated. I find the authors' conclusions and recommendations compelling, especially their suggestions for streamlining the taxonomic workflow to enhance the pace of species inventory. That said, I am not certain that there is a clear consensus within the taxonomic community on some of the proposed recommendations, which highlights the value of this work in stimulating a constructive debate around best practices.

Minor Comments:

I would appreciate it if the authors could briefly explain, in the Materials and Methods section, the meaning and relevance of the statistical indices reported in Table 1 (e.g., Skew, MAD, Kurtosis). For readers who are not trained statisticians, a concise clarification of what each index measures and how it contributes to the analysis would improve accessibility.

---Response: We thank the reviewer for their thoughtful and constructive comments, and for their careful reading of our manuscript.

Some of these metrics are already indirectly described in lines 283–291, where we summarize the values obtained for each (or nearly each) metric. However, we agree with Reviewer #1 that a brief explanation of the metrics would improve clarity. Therefore, we have added this information to the Materials and Methods section.

--- Before it read: To summarize and visualize the three periods, we calculated their durations in years for easier interpretation and representation. We obtained the central tendency and position statistics of each period using the base package in R [43], and we calculated asymmetry and dispersion statistics using the psych package [44]. We used the ggplot2 package [45] to represent the results using violin and boxplots. This process was conducted independently for each genus and region, and merging the four regional datasets to obtain global estimates.

--- Now it reads: To summarize and visualize the three periods, we calculated their durations in years for easier interpretation and representation. Using the base package in R [46], we obtained central tendency statistics including the mean, trimmed mean (mean after excluding 10% of extreme values from both ends to reduce the influence of outliers), and median, along with position statistics such as the minimum and maximum. Using the psych package [47], we then calculated measures of asymmetry, including skewness (an asymmetry coefficient where positive values indicate a long right tail, meaning most observations are concentrated below the mean with some high values) and kurtosis (a measure of tail weight and peakedness where high values indicate heavy tails and a sharp central peak compared to a normal distribution). Using the psych package, we also calculated dispersion measures such as the Standard Deviation (S.D.), Median Absolute Deviation (M.A.D.; a robust dispersion measure; calculated as the median of absolute differences from the median, less sensitive to outliers than S.D.), range, and Standard Error (S.E.). We used the ggplot2 package [48] to represent the results using violin and boxplots. This process was conducted independently for each genus and region, followed by merging the four regional datasets to obtain global estimates.

Conclusion:

In summary, this is a well-executed, well-written, and highly informative article. It presents an original contribution to the literature on taxonomic research. I have no major criticisms and only a single minor suggestion for clarification. I recommend this manuscript for publication after this small revision is addressed.

---Response: Thanks.

Reviewer #2:

This paper is an interesting read that provides information on the temporal distribution of key steps on the road to describing frog species in tropical areas. I think with careful revision it could be worth publishing. I have made some more detailed comments on a word version of the MS, but also make some broader comments here.

Tone of writing - Overall I suggest there may be room to be a bit more tempered in some of the claims made. As examples - for vertebrates (and especially terrestrial verts) it is defendable to argue that we do have moderately good framework for understanding patterns in many areas (i.e the existing framework is not a complete disaster). In developing countries, the claim that naming species leads to their protection is contestable at best, as conservation, let alone conservation of small brown frogs, is low on the priority list. In these places in the short-term individual species focused conservation is also not likely to get off the ground – broader region-based approaches are probably more likely to get traction. This is not to say the taxonomy is not important – it totally is and can feed into the above – but it is not the be all and end all to getting conservation happening in tropical countries. Basically - I suggest temper the tone a little in places.

---Response: We thank the reviewer for his thoughtful and constructive comments, as well as for his careful reading of our manuscript. Below, we respond to both the general comments and the specific remarks provided in the annotated version.

We appreciate the suggestion to temper some of our language and recognize that certain statements may have been more strongly worded than intended. We have reviewed the entire manuscript to identify and revise such instances.

Our intent was to highlight that our understanding of diversity and its spatial patterns remains incomplete and will continue like this until we achieve a complete—or at least representative—picture of actual diversity. In amphibians and reptiles, for instance, with over 100 species from each group described annually, taxonomic knowledge is clearly far from complete, reflecting the dynamic and still-developing nature of our understanding. We agree that in some regions, particularly for terrestrial vertebrates, a relatively well-developed framework exists for recognizing broad biodiversity patterns. However, regions such as Melanesia, Madagascar, and other tropical areas—where hundreds of species have been described recently and many more remain undescribed—demonstrate that substantial portions of biodiversity are still undocumented, and that our understanding is still improving and will continue to shift with further discoveries. We have revised the related text to avoid any misinterpretation.

We agree with the reviewer that taxonomy does not guarantee conservation outcomes and that conservation faces challenges unrelated to taxonomic progress. We do not claim that species descriptions directly lead to conservation, but rather that they are a necessary first step—since we cannot conserve what we do not recognize or delimit. This point has been clarified in the text to avoid misinterpretation. We also acknowledge that species-focused conservation is often impractical in regions with limited resources and low political will, which is why our discussion emphasizes the value of region-based approaches considering the undescribed (but known) species. While charismatic species may occasionally act as flagships for conservation, the documentation of multiple well-justified endemics (e.g., brown frogs) can also support habitat protection, aligning with broader goals such as 30 x 30. Ultimately, as noted, conservation decisions depend on socio-political and economic factors beyond taxonomy alone.

Results – I think these can be rationalised a bit. They are quite long at the moment, but many cases are really just verbal summaries of the tables. Try and cut them down. For instance instead of by region summaries – just say the global trends, and note where regions differ from this. The discussion of months where types where collected is not interesting and should be largely or completely cut. There is a high risk that any differences are largely idiosyncratic so I would strongly suggest focusing on the global trends.

---Response: We have substantially reduced the length of certain sections, focusing on global trends as suggested by Reviewer #2.

--- The section ‘Number of field campaings and temporal patterns of type specimen collection’ has been shortened from 402 to 159 words and now reads:

“Half of the species (476; 53.1%) described between 2000 and 2023 required a single field trip to collect the entire type series, with an average of eight collected specimens per species. Two trips were required for 20.3% of species, three for 11.7%, and four for 6.1%. The remaining ca. 8% were described on the basis of collections made over between five and 23 field trips.

Considering the four regions, species descriptions between 2000 and 2023 were based on type specimens collected between 1893 and 2023, with the majority collected between 1999 and 2016, with some regional differences (S1-S5 Fig).

In Melanesia and Ecuador (S4-S5 Fig), type specimen collection was relatively evenly distributed throughout the year (defined as each month having at least 40% of the number of specimens collected in the peak month). In contrast, Madagascar and India exhibit marked seasonal peaks in collection activity, with certain months showing higher numbers of type specimens collected than others (S2-S3 Fig).”

--- The section ‘Temporal evolution of variables’ has been reduced from 827 to 643 words, following the reviewer’s suggested rationale to focus on the most informative and broadly relevant variables.

Discussion:

Following from the above the discussion is my opinion too long, and includes a lot of general thoughts that are not well connected with the key results and/or quite sweeping. I would suggest this needs a thorough rewrite to make it much more focused.

I would also strongly suggest not framing this around crisis and solution – we have been hearing this for 20 years now (at least) and it has not achieved a great deal. For what it is worth, here is my summary based on working in Melanesia, which likely has over 800 frog species and about 5 active taxonomists (none of which are paid full time to be taxonomists). First there is not going to be a magic bullet, my uni does see describing frogs as a priority activity for me, and neither do most people in Melanesia. That said I do think it is of great value and agree with the authors. So what we can do however is look at the resources we have, the shape the job to be done, and try and prioritise things on criteria such as which things are likely to be most threatened, which descriptions are easiest, and which descriptions will have most impact. If we do this smart we may then be able to attract support to keep working on the job. When people are really keen to work in this space we can support and enable them, while trying to be honest about their job prospects, but we also can’t engineer a trained and employed New Guinea frog taxonomist out of thin air. I am not against new technologies at all, but with 5 people in the space (three which are over 60) we have to be realistic about what we can get done. I would suggest a more focused discussion about how to prioritise resources and potentially to advocate for a few more could be a useful thing that can be done here.

---Response: We appreciate the reviewer’s thoughtful comments and their candid reflection based on first-hand experience in Melanesia. We have taken this feedback seriously and revised the discussion to improve the focus and structure. We acknowledge that the discussion section is relatively long, but we believe it is necessary to properly contextualize our findings and the broader implications of our results.

While some of the discussion topics are not directly derived from our quantitative results, they emerge logically from them, and we believe they are important to address in the context of species description processes.

We did not intend to frame the manuscript strictly as a “crisis and solution” narrative. Rather, we aimed to describe and discuss the current state of species description—including its challenges. We fully agree that there is no “magic bullet,” and we do not suggest otherwise. However, we believe that strategies and pathways for improvement may help address specific bottlenecks and are, therefore, discussed.

Finally, we understand the reviewer’s point about prioritizing species for description. However, in many cases, such prioritization is difficult without first initiating the species delimitation process. Unless a species is highly distinct (e.g., morphologically unique, phylogenetically isolated, or strikingly colored), it is often not possible to anticipate how easily it can be described compared to other undescribed species, its conservation status, the impact of its description, or its broader significance. Therefore, while we agree with the theoretical importance of prioritization, we believe it is not straightforward in practice.

I have noted in the marked up word doc some other cases where sweeping statements are good in principle, but in practice can pose issues, or need to be much more fully fleshed out.

---Response: We have carefully reviewed the marked-up Word document and addressed all the noted instances. Below, you will find our detailed responses to each of the points raised.

Areas for consideration with respect to interpretation and analyses

The time from collection to description is actually shorter than I expected. For me this emphasises that we really need to keep collecting (as we and many other have stated in lit). Most new species are relatively newly collected, indicating that the reference libraries of life (museums) have collections that are far from complete. This point has been made, but given increasing hurdles to fieldwork and vouchering, it does not hurt to make it again. If we don't keep collecting - especially in poorly known areas, we are not gunna get the job done.

---Response: We agree that the fact that a substantial proportion of species were collected only a few years prior to their description is significant and worth highlighting. We have therefore revised the relevant paragraph to further emphasize the importance of continued collecting:

--- Before it read: Only 36% of species described since 2000 were collected within a five-year timeframe, suggesting that new species are rarely identified in the field and that targeted sampling campaigns are uncommon. The remaining species were collected earlier —up to 125 years ago— and described much later. This underscores the importance of public collections as reservoirs of potential new species but, at the same time, it concerns about the lack of resources, time, or interest dedicated to examining the collected material.

--- Now it reads: Thirty-six percent of frog species described since 2000 were named within five years of first collection, highlighting the importance of continued collecting. A substantial proportion of recently described species rely on newly collected material, which underscores the need to maintain active fieldwork, particularly in poorly known areas, at a time when logistical, political, and regulatory barriers to collecting and vouchering are increasing. In contrast, the remaining 64% of species were collected earlier —up to 125 years ago— and described much later. This suggests that new species are not always recognized as such in the field, or, if they are, their formal description is not prioritized. It also reflects that targeted sampling aimed at collecting specific, suspected new species is relatively uncommon. They also support the critical role of public scientific collections as reservoirs of undocumented diversity, while also raising concerns about the lack of resources, time, or interest dedicated to examining the collected material.

Is lag time correlated with the number of active taxonomists? I know in Melanesia there are only four living scientists that have described more than five species in this region. I suggest an analysis of how numbers of active authors may influence description speed. This could also illustrate how few people are working on some diverse faunas which would show how tenuous all this knowledge actually is.

---Response: Thank you for this valuable suggestion. In the first submission, we did include the number of active taxonomists as a variable in our analysis as can be seen in the table S2 'N_aut_taxon'.

Defining who qualifies as an "active taxonomist" is not straightforward, as (arguably) any author listed on a species description could be considered one.

To address this, we extracted the names of all authors from taxonomic papers published since 2000 and counted the number of times each name appeared across the dataset. We then defined "active taxonomists" as those whose number of appearances exceeded the mean plus the standard deviation. This threshold allowed us to identify highly recurrent contributors. Using this approach, in Melanesia, we identified S. J. Richards, R. Günther, F. Kraus, P. M. Oliver, and A. Allison as active taxonomists—each appearing at least 20 times in species descriptions after the year 2000. This, we think that seems to align well with 'active taxonomists' in that region. However, in regions like Ecuador, this threshold excluded some significant but less active (in the last 20 years) taxonomists, whose expertise and impact are nonetheless important.

When we attempted to include the number of active taxonomists per paper as a variable, we found it to be highly correlated with the total number of authors. Due to this strong correlation, we excluded the "number of active taxonomists" from the final model to avoid redundancy, as both variables would have explained

essentially the same variation. Instead, we retained the total number of authors to also test the "author inflation" hypothesis.

I would suggest the 11.3 year lag is likely an underestimate going forward, as the sample is biased towards frogs that are being described. The frogs that remain undescribed are increasingly, the hard ones – complexes of confusing or morphologically similar species that are difficult to resolve. One could speculate that lag time may increase as we increasingly get stuck on these species. But only put this in if space opens up – more important in my opinion to rationalise and focus discussion around some of the points raised earlier.

---Response: Thank you for this thoughtful observation. We understand the concern that our estimate of an 11.3-year lag may underestimate future delays, especially as some species pending description are likely part of morphologically cryptic or complex groups (i.e., the difficult ones) that may require more time and advanced tools (e.g., molecular data) to resolve. However, our dataset already includes a number of species described since 2000 that belong to such complexes, suggesting that some of these challenges are already reflected in the current lag time. Moreover, many species in our dataset were collected over 50 and up to 125 years ago, reflecting these 'difficult cases' and counterbalancing the inclusion of more recently collected, 'easier to describe' species. We believe this helps capture a range of descriptive difficulties, although we agree that lag times may increase as taxonomic efforts increasingly focus on the more complex portions of biodiversity.

Keywords

Crisis is misspelt, order words alphabetically

---Response: thanks for the observation, we have made the changes.

6. PLOS authors have the option to publish the peer review history of their article (what does this mean?). If published, this will include your full peer review and any attached files.

If you choose "no", your identity will remain anonymous but your review may still be made public.

Do you want your identity to be public for this peer review? For information about this choice, including consent withdrawal, please see our Privacy Policy.

Reviewer #1: No

Reviewer #2: Yes: Paul Oliver

[NOTE: If reviewer comments were submitted as an attachment file, they will be attached to this email and accessible via the submission site. Please log into your account, locate the manuscript record, and check for the action link "View Attachments". If this link does not appear, there are no attachment files.]

While revising your submission, please upload your figure files to the Preflight Analysis and Conversion Engine (PACE) digital diagnostic tool, https://pacev2.apexcovantage.com/. PACE helps ensure that figures meet PLOS requirements. To use PACE, you must first register as a user. Registration is free. Then, login and navigate to the UPLOAD tab, where you will find detailed instructions on how to use the tool. If you encounter any issues or have any questions when using PACE, please email PLOS at figures@plos.org. Please note that Supporting Information files do not need this step.

Reviewer #2 attached file comments:

L63 True for many groups of inverts, probably not true for plants and vertebrates, suggest clarify

---Response: In this paragraph we are talking about global diversity (in general) for which the statement 'largest proportion of biodiversity is comprised by undescribed species' is completely applicable.

L80 There its now a good ref for barcoding suggesting high levels on unrecognised diversity in Melanesia as well - file:///Users/s2984027/Downloads/biogeography-18-001_article-137988_en_1-4.pdf

---Response: Done. We have added this reference in three different sections of the paper.

L93 Often these steps are not linear like this though, there can often be back and forth while working what units are worthy of the designation of ‘species”

---Response: We have partly rephrased to include the reviewers’ point.

--- Before it read: Regardless of the chosen approach, describing a species involves five steps, each depending on the previous one —the five D’s of taxonomy [36]: discovery, delimitation, diagnosis, description and specimen determination (although not necessarily by the same person). None of these steps can be achieved without previous field work.

--- Now it reads: Regardless of the chosen approach, describing a species involves five steps —the five D’s of taxonomy [38]: discovery, delimitation, diagnosis, description and specimen determination (although not necessarily by the same person). While these steps are conceptually linear, in practice, the process is often iterative, with researchers revisiting and redefining previous steps as new information emerges or as the species boundaries are reassessed. None of these steps can be achieved without previous field work.

L94 Good point - I like this

---Response: Thanks.

L97 Suggest list some different possible data sources here

---Response: We have added some lines of evidence typically used in herpetology.

--- Before it read: To decide whether a lineage merits to be named, data from different lines of evidence should ideally be gathered, which requires time and cannot always be achieved within a single field campaign.

--- Now it reads: To decide whether a lineage merits to be named, data from different lines of evidence (e.g., morphology, genetics, bioacoustics, ecology) should ideally be gathered, which requires time and cannot always be achieved within a single field trip.

L113 We coined a term for this - ‘cryptic extinction risk’ which may be useful here - https://link.springer.com/article/10.1007/s10531-022-02412-x You could also cite the “dark extinction” papers here

---Response: We appreciate the suggestion and have now include the reference. While we recognize the relevance of the term “cryptic extinction risk”, we have opted to retain the term “silent extinction”, given its broader use.

L118 I think reframing these as aims rather than questions reads a bit better

---Response: We have followed the reviewer suggestion and changed the goals from questions to aims.

L135 And largely in practice..

---Response: We appreciate the suggestion. Indeed, we found that only a few taxonomists (sensu lato—that is, authors involved in species descriptions) have participated in species descriptions across more than one country (e.g., R.K. Abraham, A. Fouquet, A. Hamidy, C.R. Hutter, J. Köhler, A. Ohler, and R.A. Pyron), and their involvement in two countries simultaneously is very limited. We have therefore updated the sentence to reflect that the selected regions are taxonomically independent both in principle and largely in practice, as suggested.

--- Before it read: These regions are taxonomically independent and, in principle, do not share taxonomists, which helps minimize regional biases in research practices.

--- Now it reads: These regions are taxonomically independent and, in principle —and largely in practice— do not share taxonomists, which helps control for regional biases in research practices.

L151 There is an ongoing discussion about whether to include the Maluku biota as part of the Melanesian biota. I think either approach is defendable, but you might want to check whether your search terms ending up capturing this region or not

---Response: We did not include the Maluku biota because ASW only allows selection of the “South Moluccas” region, which represents only the southern part of the Maluku Islands. Since this filter does not cover the entire Maluku archipelago, we chose to exclude all Maluku Islands from our analysis. Additionally, using the “South Moluccas” filter yields five species, all described before the year 2000. As our analysis focuses exclusively on species described after 2000, applying or omitting this filter would have no effect on the results.

L157 This is true, but focusing in on Melanesia, it remains true that a vast majority of descriptions are still based largely on morph and calls, so yes we have new tools, but in very poorly known areas, the older approaches often remain very dominate, reflecting the fact that these biotas are still rife wth obviously different frogs

---Response: We agree with Reviewer #2’s observation. As our results show, Melanesia currently lags behind in the use of molecular data, with fewer than 40% of species descriptions including genetic data. However, this is likely to change soon, given the recent development of a comprehensive mitochondrial barcoding database for the entire region (Ferreira et al., 2025). Furthermore, we believe that the decision to use molecular data in species descriptions often reflects the author’s preference rather than an absolute necessity. Incorporating molecular data does not automatically imply that the species are cryptic relative to their congeners, just as relying solely on morphological traits does not necessarily indicate a larger number of obviously distinct frog species compared to other regions.

L195 Authors discretion here - but I feel like field campaign is a bit dramatic - suggest fieldwork and field trip is fine!

---Response: We have replaced all instances of “field campaign” with “field trip” throughout the manuscript.

L233 This is for the pooled 4 region dataset right, perhaps make that super clear?

---Response: Yes, we have clarified.

--- Before it read: We evaluated the temporal evolution of several variables including the overall description process, the collection, the description sensu stricto…

--- Not it reads: For each region and globally, we evaluated the temporal evolution of several variables including the overall description process, the collection, the description sensu stricto...

L239 Ok this answers my question I think, but perhaps add a clarifying section above as well

---Response: Please, see previous comment.

L241 As written not clear what the percentage is of - percentage of descriptions using genetic evidence?

---Response: We have rephrased.

--- Before it read: In addition, we counted the number of species described each year and the number of species described using genetics to compute the percentage.

--- Not it reads: Additionally, we counted the number of species described each year and the number of those that used genetic data, in order to compute the percentage of species descriptions incorporating genetic evidence per year.

L250 Not a competition - but yay for Melanesia!!! :)

---Response: Haha, yes!

L276 All this is in the table, is possible to just give the verbal summary, and point to the table for the numbers?

---Response: We agree that the essential information is already presented in the table; however, since these represent the main results, we prefer to keep the verbal summary in the text for clarity. That said, we have substantially shortened other sections as suggested by Reviewer #2 to balance the manuscript’s length.

L281 I guess Melanesia does not win the competition here!

---Response: Given the points Reviewer #2 raised during this revision, we fully understand that Melanesia does not come out on top in this comparison.

L297 This probably not important, but in a lot of places I feel like one decimal place would be a more meaningful cut-off to use in this paper

---Response: We reviewed the entire manuscript (except the tables and p-values) and reduced the number of decimal places to one for better clarity and consistency.

L303 I will bet a lot go money some of this is an artefact of the fact that this is when universities are not teaching, and lecturers can go into the field - I would be inclined to downplay these monthly patterns somewhat - I think other parts of the paper are more interesting

---Response: Following the recommendation, we have significantly shortened this section (see general Result's comment). While we agree that some of the monthly patterns may be influenced by external factors such as academic calendars, we believe a brief mention still adds useful context regarding seasonal biases in sampling.

L305 These summaries are basically the same as what is in the figures, could consider cutting or further rationilising

---Response: We have significantly reduced the length of this section (see previous comment and general comment).

L317 The main researchers in this region have not had lecturing duties, which may explain the broader spread of collections across the year. Again I don't think this is supper interesting and would suggest cutting.

---Response: We have significantly reduced the length of this section (see previous comment and general comment).

L347 That's an interesting bit of information

---Response: We agree, and we have addressed it in the discussion section.

L350 As above - I found this section to detailed, my strong suggestion would be to go through and rationalise. For instance you could give global trends, but only mention regional trends when they conflict, and otherwise point to the figures

---Response: The section 'Temporal evolution of variables' has been reduced from 827 to 643 words following the reviewer's suggested rationale. However, the evolution of many of these variables is interesting in itself, so it is not straightforward to reduce the length without losing relevant information.

L357 Is this basically saying same thing as first two sentences?

---Response: Yes, we have removed the sentence.

L420 Arguable - EO Wilson was onto this in the 80/90's, Gerald Durrell was writing about it in the 60 and 70's… Could cut these last two sentences, and join with next paragraph

---Response: We agree with the reviewer that awareness of the extinction crisis began decades ago, as reflected in the work of Wilson, Durrell, and others. Our original use of "generation" was meant to refer to all people currently living on Earth. However, we recognize that this wording may have been ambiguous and have rephrased the passage accordingly.

--- Before it read: We are the first generation to fully comprehend that we are in the midst of an extinction event, while the majority of species remain undiscovered and undescribed. We may also be the last generation having the opportunity to explore and document Earth's biodiversity [37, 51].

--- Now it reads: In the past century, we have come to realize that our planet is facing a biodiversity crisis, while the majority of species remain undiscovered and undescribed. Unless we reverse the current trend of

threats, we may be among the last generations with the opportunity to explore and document Earth's biodiversity [39, 54].

L444 Don't follow the logic here, in my experience often the opposite also occurs, things are thought to be new, but careful reveals they are not

---Response: We agree that both scenarios can occur and this sentence could be misinterpreted. We have rephrased the paragraph to improve clarity and avoid overgeneralization. Our point is that if 64% of species were described more than five years after collection, this suggests that many new species are either not recognized in the field or are not prioritized for description. In our view, if a species is clearly recognized as new in the field, it should not take ten years or more for its formal description. We acknowledge that delays can occur for many reasons, but taking a decade (or much more) to describe something identified as new from the outset seems excessive, and the less parsimonious explanation. We have rephrased to make it clearer and to include the crucial role of keep collecting.

--- Before it read: Only 36% of species described since 2000 were collected within a five-year timeframe, suggesting that new species are rarely identified in the field and that targeted sampling campaigns are uncommon. The remaining species were collected earlier —up to 125 years ago— and described much later. This underscores the importance of public collections as reservoirs of potential new species but, at the same time, it concerns about the lack of resources, time, or interest dedicated to examining the collected material.

--- Now it reads: Thirty-six percent of frog species described since 2000 were named within five years of first collection, highlighting the importance of continued collecting. A substantial proportion of recently described species rely on newly collected material, which underscores the need to maintain active fieldwork, particularly in poorly known areas, at a time when logistical, political, and regulatory barriers to collecting and vouchering are increasing. In contrast, the remaining 64% of species were collected earlier —up to 125 years ago— and described much later. This suggests that new species are not always recognized as such in the field, or, if they are, their formal description is not prioritized. It also reflects that targeted sampling aimed at collecting specific, suspected new species is relatively uncommon. They also support the critical role of public scientific collections as reservoirs of undocumented diversity, while also raising concerns about the lack of resources, time, or interest dedicated to examining the collected material.

L455 This is extremely arguable. In the places I work we desperately need both, there are literally 5 people describing species in a fauna that we estimate contains hundreds of candidate species and vast areas of uncollected terrain. Please do not write this as we do not need more people coming up reasons why we can't fill these massive sampling gaps we have. I will also argue that feildtrips often produce the easy to described species that can be done quickly, I would guess part of the slow down for Melanesia is linked back to a slowdown in fieldwork. So this means we are left dealing with the hard slow species, while also failing to fill the massive geographic gaps that remain unsnapled

---Response: Thank you for pointing this out. We fully agree that both increased collecting and increased taxonomic capacity are urgently needed, particularly in regions like Melanesia where the number of candidate species far exceeds the number of people working on them. Our intention was not to discourage collecting efforts, but to emphasize that their full value is only realized when material is properly processed and revised. To avoid misinterpretation, we have reworded the paragraph to clarify this highlighting the need to reverse the funding cuts in taxonomy.

--- Before it read: In the current context of the biodiversity crisis, some authors have suggested prioritizing the collection and preservation of as many specimens as possible as testimonies of a vanishing biodiversity, ensuring future generations can describe species that may already be extinct [37, 59]. However, unless this strategy is paired with thorough revision of collected material, it may result in decades of specimens remaining unexamined on shelves, delaying species descriptions that could contribute to their conservation.

--- Now it reads: In the context of the current biodiversity crisis, some authors have suggested prioritizing the collection and preservation of as many specimens as possible as testimonies of a vanishing biodiversity, ensuring future generations can describe species that may already be extinct [39–63]. Collecting efforts are essential for addressing the geographical and taxonomic gaps that still exist. To maximize their contribution to biodiversity discovery, collecting should be paired with proper specimen processing (including tissue

sampling for genetic analysis) and thorough taxonomic revision to promptly detect candidate species and prioritize their description.

L458 Honestly - this statement is frustrating - we have had 20 years of people talking about how to fix the crisis in taxonomy. The reality here is that it takes time, there is no quick fix, and there are fewer and fewer jobs. We can prioritise the resources we have to try and ensure maximum impact and try and advocate for more resources. But please stop pretending there is some magic fix around the corner, and we need to start talking about how we are going to prioritise the resources we do have to maximise value, rather than pretending we can somehow get it all done in the next 10 years.

---Response: We thank the reviewer for highlighting the complexities in addressing the taxonomy crisis. We have revised the section to emphasize the critical need for increased funding and streamlined methods.

--- Before it read: To ensure species are described while they still exist, it is necessary to adopt streamlined species description methodologies (e.g., “fast-track taxonomy”), the integration of molecular data to support rapid identifications, and increasing funding for taxonomic research.

--- Now it reads: To describe as many species as possible while they still exist, it is essential to reverse decades of funding cuts to taxonomy. Increased investment would support the creation and maintenance of much-needed taxonomic positions, expand sampling efforts, and enable thorough scrutiny of both recent and historical collections. In parallel, adopting streamlined species description methodologies (e.g., “fast-track taxonomy” in the sense explained above) and integrating molecular data to support rapid identifications can improve efficiency of species discovery and descriptions.

L460 Yes molecular data is good, but it is not nessecary in all cases. And this ignores the fact that many countries don’t have access to these resources, or won’t allow export to specimens to countries that do. So yes in principle, and we should certainly take and lodge tissues, but in practice sweeping statements like this often create issues for situations where they are not feasible to apply in practice.

--- Response: Thank you for this important comment. We have revised the paragraph to acknowledge the value of molecular data while also recognizing the practical limitations of its collection and use.

--- Now it read: Large-scale molecular screenings are still relatively rare, but those conducted have revealed high numbers (even hundreds) of potential new species—enabled by comparisons with previously sequenced taxa [10, 30, 32, 55]. Despite growing use of molecular data in species descriptions over the last decades, it has yet to become a standard practice (see results; [53]). Our results suggest that incorporating molecular data into frog descriptions significantly increases description time in some regions. While adding any procedure to the species descriptions inherently increases the time required, the increase of time can also be attributed to cryptic species, which may remain undescribed on shelves for years until identified molecularly. Sequencing type specimens during species descriptions offers benefits for taxonomy and biodiversity science. DNA-barcodes linked to identified and re-examinable type specimens establish a reliable connection between genetic data and physical vouchers from the outset [66]. This enables unambiguous specimen assignment to newly described species often without taxonomic expertise—except in cases of introgression, hybridization, or human error [67]. Therefore, by leveraging new methodologies and increasingly affordable molecular tools, the widespread use of DNA barcodes to screen collected material should ideally become a standard practice among taxonomists where feasible, especially in poorly surveyed regions [68].

L464 This is simply not true, temper and give context.

---Response: We have revised the sentence to provide additional context and accuracy.

--- Before it reads: Earth’s tropical regions remain largely unexplored.

--- Now it reads: Tropical regions harbor most of Earth’s terrestrial biodiversity, yet they remain highly threatened and among the least scientifically understood [64–65].

L473 Again good in principle, but in reality barcoding often just highlights moderately divergent lineages that are difficult to interpret and require detail analyses, and often further collections to identify turnover and contact points - see comment above about sampling

---Response: We partially agree with the reviewer. As already stated in the manuscript, “the reliance on barcodes alone does not provide sufficient evidence for robust species delimitation.” Importantly, we consistently refer to these lineages as ‘candidate’ species, not as ‘new’ species. This distinction underscores that such lineages must undergo taxonomic scrutiny to decide if there is enough evidence to name them. Nonetheless, we argue that making genetic data from geographically explicit samples publicly available plays a crucial role in drawing attention to previously unrecognized biodiversity. Highlighting candidate species and making their data publicly available not only fosters future taxonomic assessments but also contributes valuable data for other biological disciplines. While we acknowledge that mitochondrial divergence alone is not definitive, in many cases (with exceptions), deeply divergent mitochondrial lineages do correspond with nuclear differentiation and ultimately result in the recognition of distinct species.

--- Before it read: Genetically characterizing the collected material and making the sequences with specific localities publicly available would not only highlight candidate species, thereby promoting their formal description, but also provide valuable data for other disciplines.

--- Now it reads: Genetically characterizing the collected material and publicly releasing the georeferenced sequences would not only highlight candidate species—thereby promoting their formal description—but also provide valuable data for other disciplines such as biogeography, evolutionary biology, and ecology.

L501 Agreed

---Response: Thanks.

L509 I feel like this paragraph emphasising the need to pragmatism, directly conflicts with the earlier section which states that everything must be barcoded

---Response: We appreciate the reviewer’s concern. We believe there is a clear distinction between the comprehensive inclusion (in the original description) of natural history, biogeography, or osteological data and the inclusion of a minimal genetic marker (e.g., a DNA barcode), which we argue should be considered standard practice, when the sociopolitical context allows it. Including basic genetic information in species descriptions not only facilitates future identifications—especially for non-specialists or early-career taxonomists—but also enables a broad range of downstream analyses across disciplines.

In this sense, our recommendation for including barcodes is not at odds with the call for pragmatism, but rather reflects a practical and scalable baseline that enhances taxonomy. For example, while providing a full osteological description can be scientifically valuable, its utility in future comparative studies (unless osteology is the only line of evidence supporting the species hypothesis) often requires specialized training. On the other hand, sequencing a short gene fragment (e.g., ~300 bp) allows for straightforward comparisons—even by early-stage researchers—and makes the identity of the species more accessible, unless specific complications like mitochondrial introgression are present.

To make this concrete: consider a young zoologist conducting fieldwork in Ecuador who needs to work with the genus *Pristimantis* for the first time. Having access to a reliable barcode database would greatly ease the process of species identification, compared to relying solely on morphological traits, which often require lot of time (comparing many specimens) to get familiar with. We acknowledge that this is partly a matter of perspective, but we strongly believe that incorporating basic genetic data is one of the most effective and pragmatic ways to advance taxonomy in diverse tropical regions.

L516 THis para has some interesting ideas, but does not follow through on them

---Response: The paragraph was intended to raise key questions rather than fully resolve them, as determining minimum standards for species descriptions or deciding how comprehensive they should be is beyond the scope of this manuscript. Our goal was to introduce these unresolved issues as a way of framing the discussion that follows in subsequent sections.

L522 This is true, but it increasingly feels like this discussion is a wide ranging journey through lots of ideas that are not really clearly linked back to the key results of the análisis

---Response: In this paragraph, we introduce a major impediment to taxonomic progress: the limited accessibility of taxonomic data and the lack of standardization when such data are available. This point

directly connects to the following paragraph, where we discuss the potential of cybertaxonomy to address these challenges. While some parts of the discussion are not direct outputs of our quantitative analyses, they emerge logically from our findings and are relevant to the broader context of species description practices. Indeed, the practical difficulties of accessing type specimens, locating data papers, dealing with non-standardized data, or physically traveling to multiple collections are issues likely encountered during the description of many of the ~900 species analyzed in our study. We believe these are critical topics to raise in order to contextualize the structural limitations that affect taxonomic workflows today.

L531 Can we please stop pretending there is a “solution” - we need to look for ways to streamline and speed up the process where possible I agree, but pretending there is some way to magically speed up the rate of species descriptions x10 with existing resources is not really helping anyone. By far then most productive frog taxonomists are sill doing it largely the old-fashioned way (although sometime using newer data types) - they are just more organised, more resourced and/or more efficient than others - I don’t see anything changing that in the near future

---Response: We want to clarify that we did not intend to frame the manuscript as a “crisis and solution” narrative. Our goal was to describe and discuss the current state of species description, including its challenges. We fully agree that there is no “magic bullet,” and we do not suggest otherwise. However, we believe that some strategies and pathways may help address specific bottlenecks, which is why we included them in the discussion. To reflect this nuance, we have replaced the word “solution” with “strategy” throughout the manuscript.

Regarding the second point, the main goal of this paragraph is to emphasize the need for publicly available and easily reusable data—something that, whether an author uses morphology alone or multiple lines of evidence, is likely to be widely agreed upon.

L538 You would need make the description ICZN compliant (not trivial). Is this a platform just for frogs? Is there a peer-reviewed process involved here? This is an interesting idea - but there is a lot of complexity here.

---Response: We have now included this relevant information in the revised paragraph, which was previously omitted. The scientific work and the naming process will remain ICZN-compliant and follow the standard procedures, including submitting drafts for peer review, editing, and publication. The cybertaxonomy (which could be useful for all taxa, not only for frogs) begins after this traditional process. At that stage, all data associated with the paper should be uploaded in a standardized format to facilitate easy submission—either by the journal or the author—into a publicly accessible platform. Additionally, we have specified that any information uploaded to the platforms should be traceable, either through ORCID-identified users or associated published bibliography.

---The new paragraph now reads: A previously suggested strategy to enhance data accessibility and thereby accelerate species descriptions, without compromising species hypothesis is “wikifying” taxonomic data (i.e., cybertaxonomy [83–86]). This approach integrates traditional taxonomic practices with modern digital platforms. The formal description would continue to follow (unless a better solution is found) established taxonomic procedures (i.e., peer-reviewed publications that comply with the ICZN). However, information about a species or candidate species would be available on a centralized digital platform uploaded by ORCID-identified researchers or through referenced literature. This would draw attention to these undescribed lineages and raise awareness among colleagues working on or possessing information about the same taxa [21, 87–88]. If centralized, this system would liberate taxonomic data from conventional publication formats, make it easy to find all information about a species, and facilitate the continuous updating of species descriptions and data before, during, and after their formal description (e.g., DNA sequences, traits, and distributional data). In addition, it could promote the release of data that, due to their magnitude or nature, are not usually published but are nonetheless of interest (e.g., morphological measurements of already described species). This strategy would allow the mass description of species following a (to be defined) minimum standard to ensure reliable descriptions, with the possibility of later adding information to update them ([21, 53]; and references therein). Consequently, it would not be necessary to delay naming species to provide the most complete description possible. Species could be included in the conservation scheme from the outset, and although the act of naming itself remains static, their descriptions and biological content could remain dynamic and be updated over time. Leveraging the

fact that a small number of journals are responsible for the vast majority of published taxonomic papers, these leading journals—as suggested by Miller et al [86]—could take the lead and coordinate with other journals to request standardized taxonomical data (e.g., morphometric values, variable labelling, coordinates) and routinely upload it to digital platforms. Once a shared minimum standard for reliable descriptions and a standardized data submission format is established, other journals and authors could follow the same templates, ensuring consistency and broad adoption across the field.

L542 Only once someone has gone through and given them a realist status or simailar - even most described species are effectively invisible to the average conservationist, especially in the tropics

---Response: We will likely agree that our world is failing alarmingly to protect biodiversity. However, under the current rules, once a species has been formally described, it becomes possible to take conservation action if the necessary resources and, above all, the political will are in place. Matters such as indifference toward species loss fall outside the scope of this manuscript.

L546 Legislation in some does protect populations or other below species units, so this statement as worded is generally true, not nut correct

---Response: To avoid any misinterpretation, we have removed this sentence. However, we refer specifically to undescribed species that often, prior to formal description, do not qualify even as populations—though in some cases they may be considered divergent populations. Essentially, these entities do not exist until described.

L547 This is fine as you refer the IUCN specifically

---Response: We have removed the previous sentence to specifically refer to the IUCN.

L551 Careful with wording here, it makes it sound like the taxonomist are dithering around unaware of what is happening, in some cases this is true, but in others they are totally aware and desperate to get things names, but just face many constraints on getting the work done

---Response: We have rephrased to avoid any misinterpretation.

--- Before it read: In some cases, taxonomists describe species whose existence has been known (to specialists) for decades, and when they finally name them, they realize that the locality where it was first collected has been completely destroyed

--- Now it reads: In some cases, taxonomists describe species whose existence has been known (to specialists) for decades, but because formal naming can be delayed by multiple external constraints, when they finally name them, they sadly realize that the locality where it was first collected has been completely destroyed

L554 In Melanesia - I would say whether things are named of not does not have a massive impact on their conservation prospects - it might improve them slightly on balance, but other factors are probably more important - like whether the world warms 2 or 4 degrees, or whether chytrid gets into the island or not

--- Response: Melanesia is likely an exception compared to other tropical regions. It harbors immense biodiversity while experiencing low levels of habitat destruction. In many other tropical areas, high biodiversity coincides with extensive deforestation, which creates a different dynamic. In an ideal world, if there is sufficient will and scientific evidence, deforestation can be locally halted, potentially improving conservation outcomes.

L557 I think this is true - but again potentially much less so in tropical regions

---Response: As the reviewer pointed out, protecting populations is indeed viable under certain circumstances; however, it is generally the species level that receives most attention. Issues such as indifference toward species loss, while important, fall outside the scope of this manuscript.

L556 I agree this is an interesting idea. The question would be whether this will be picked up by non-specialists in their work to prioritise regions. One intermediate step here is that taxonomic specialists can

use there knowledge of undescribed diversity to try and map and highlight key areas - See Fred Kraus's work on Milne Bay Province for an example

Response: The purpose of publishing genetic and distributional data on undescribed species is not only to prioritize regions but also to enable a wide range of downstream analyses at different scales. Such data allows other researchers, often using methodologies unfamiliar to taxonomists, to uncover meaningful biodiversity patterns. Once a sequence is available in GenBank and can be geographically attributed to a region, many studies can utilize this data. In fact, it has already been used in macroecological and phylogenetic studies to prioritize taxa and understand biogeographic patterns (see for example; https://www.pnas.org/doi/10.1073/pnas.1714891115, https://onlinelibrary.wiley.com/doi/full/10.1111/jbi.14491, https://www.nature.com/articles/s44185-023-00024-4,).

Reviewer #2 Word Comments

Reviewer 2 made numerous changes to the text. We have accepted the vast majority of them. The changes that were not accepted are discussed below:

L32 new species descriptions accumulate *similar rapidity*. We have not accepted this change because candidate species are flagged by the hundreds, while species descriptions increase at a steady rate of approximately 100 species per year.

L51 Populations *of animals and plants* are being reduced, contracted, and extirpated; species are becoming extinct at an unprecedented rate… We have changed the sentence using 'Wildlife instead of 'animals and plants'.

L102 This is especially true for frogs, where obtaining call recordings —often a source of key diagnostic characters— can be ~~daunting~~ *time-consuming*. The phrase "time-consuming" appears earlier in the text, and we wish to avoid repetition. More importantly, we feel that "daunting" better captures the broader challenges involved in obtaining call recordings—such as logistical difficulties, species-specific behavior, and environmental constraints—which go beyond the issue of time alone.
