# Supplementary material for: Croaking for haste: How long does it take to describe a frog species since its discovery?
Source: PLoS One. 2026 Jan 23;21(1):e0323855. doi: 10.1371/journal.pone.0323855 (PMC12829843; doi:10.1371/journal.pone.0323855)
Supplement: Attachment — Submitted filename: 0_Response_to_reviewers_auresp_2.pdf [file pone.0323855.s019.pdf]

MS #PONE-D-25-19328R1 *Croaking for haste: How long does it take to describe a frog species since its discovery?*

Authors' replies to the comments of the Editor and Reviewers

We thank the editor and the reviewers for their positive and constructive comments. We have followed almost all of these. We reply to each comment in the following pages and explain how we dealt with them during the manuscript revision. Please, find below our responses (in black) to the editor's and reviewers' comments (in blue). Each response is preceded by "---Response" for easy identification. All changes in the newly uploaded manuscript have been highlighted using track changes. We hope that the manuscript is now suitable for publication in PLOS ONE.

>> Reviewer #2

Reviewer #2: I have now reviewed this manuscript for a second time. I think the changes that have been made greatly improve clarity and readability, and I appreciate that the authors have taken time to respond to all suggestions.

I have gone through and tried to make some comments on the revised pdf on areas that could be potentially further tidied up.

In general - my opinion is still that discussion and small parts of the introduction are not very convincing and in places are not really providing helpful solutions that are going to address the issues in place. I would be the cutting down the discussion of how we can use novel approaches to speed up taxonomy, and focusing in the key messages that your data supports - a) it still takes a while to get things described and this lag might be increasing, b) there is still much we don't know as evidenced by most new species being recently collected, c) hence we need to keep collecting to have any hope of filling these gaps, and d) under-resourced museums and taxonomists are critical to meeting this challenge.

More specifically I'm sorry I did not pick this up earlier but I think part of the issue here is that dichotomy between fast-track and traditional taxonomy is not very clear or at all convincing (at least to me). Yes we should keep looking at ways to make things more efficient - I agree. But what exactly is being proposed to be cut from a "typical" descriptions to make things quicker? In my experience the deeper you dig into this the harder it becomes to work out what you are going to cut - especially for the god-awful species complexes we increasingly have to work with in groups like frogs - this is where you need more detail to actually understand what you are looking at. yes you can cut some of the detailed descriptive stuff - but in practice this actually often does not take very long and is often very brief anyway - so cutting it will not in fact save enormous amounts of time. There is also a trade off here in making descriptions useful versus quick. This is not an argument to better define fast-track taxonomy, it is argument to cut down reference to it completely as I actually don't think it is in practice really much different that what is being done or a 'solution".

Following on this I will say that my general point in the first review with the summary of the Melanesian frog fauna was that with ~5 people really active in taxonomy, and most of them spending 50-75% of their time doing other things - fast-track taxonomy, wiki-style sharing of data etc etc is not really going to achieve help that much (and indeed may just give the small number of people working the problem a world more work to do). The issue is capacity. I do understand that part of the idea here is that by emphasising the new approaches it has the potential attract new support and resources to this work, however I think in practice most of what is suggested is no game changer, and the only real game changer here is more funding and more taxonomists (or even just funding for the taxonomists that exist to do taxonomy!). I concede the story might be different in other areas that have more workers with potentially better resourcing that I don't know so well.

---Response: We thank Reviewer #2 for his thorough revision of our manuscript and the detailed, constructive feedback provided across both review rounds. We have carefully considered all of these suggestions and implemented most of them.

We would like to emphasize that the primary goal with this manuscript has never been to provide a radical change to taxonomy to overcome the Linnean shortfall. As specified in the title, our goal is to descriptively quantify the time required to describe a frog species. We also discuss several factors that can negatively affect the time, and discuss approaches that may improve it, but never as a magic solution. We fully agree

that additional funding to support existing taxonomic positions, create new ones, and enable more extensive sampling and in-depth examination of biological collections is crucial, especially because there is a substantial amount of work to be done and, comparatively, very few researchers. This is now explicitly stated in the discussion.

Following Reviewer #2's recommendation, we have substantially reduced and refocused the discussion, particularly the section previously titled '*Types of species descriptions and future perspectives*', which is now renamed '*Taxonomic data'*. In this revised section, we have removed the discussion of different 'types' of taxonomy. The section now provides a concise focus on taxonomic data availability. We strongly believe that making species sheets easily accessible and including all known data for each species would greatly enhance taxonomy, substantially reducing the time required to compile the necessary datasets for species comparisons. The reviewer's concern regarding an increased workload is understandable. However, in practice, the main difference from current procedures (assuming the responsibility falls on the author, not the journal) would be standardizing data and uploading it to a centralized repository—a process analogous to submitting sequences to GenBank.

The main discussion points highlighted by Reviewer #2 (a—d) are addressed in the previous section 'Anuran species description amid a biodiversity crisis'.

To further clarify the usefulness of Fast-track taxonomy approach, we refer to Riedel et al., 2013, which we cite multiple times in our manuscript. In their study, the authors discuss how streamlined and concise species descriptions can accelerate the process by up to twentyfold and outline seven specific factors contributing to this increased efficiency.

To give a final bit of clarity and perhaps a warning about tone I also quote one of the response to review comments here:

"Our point is that if 64% of species were described more than five years after collection, this suggests that many new species are either not recognized in the field or are not prioritized for description. In our view, if a species is clearly recognized as new in the field, it should not take ten years or more for its formal description. We acknowledge that delays can occur for many reasons, but taking a decade (or much more) to describe something identified as new from the outset seems excessive, and the less parsimonious explanation."

To be honest, this comes across as a staggeringly naive thing to say - I know people who have been working on taxonomy of groups in both Australia and New Guinea that have dozens to hundreds of taxa that they are desperate to describe, but due to the massive amount work involved in even basic characterisation and diagnosis of these species, the sheer numbers of new species that need to be described, and the fact that more often than not no-one is paying them to do this work, and the fact they want to sleep at night - the descriptive process is taking decades or more.

I guess my general point here is that yes we should absolutely be looking for ways to characterise issues and push things forwards and speed things up when we can, but these impediments and issues exist for many and complicated reasons and people have been thinking about it for decades. So I think focus more on the data you have and what it shows, and acknowledge that the main impediments are not procedural, but time and resources (with perhaps a large dash of legislation and bureaucracy for extra flavour).

I will leave it to editor and authors as to how much they want to act on this - I think the data as presented is fair and reasonable and summaries are interesting. And hopefully this is review helpful it is genuinely meant as to be - it is an interesting paper, and certainly question of how well we are doing in documenting the fauna of these poorly-known regions and groups is an important one to keep track of.

---Response: We completely agree that the issue is multifactorial, and that the limited number of taxonomists, the large number of undescribed species, and the lack of funding all contribute significantly to these delays. Our intention was not to underestimate these challenges, but rather to highlight that such delays have important implications for biodiversity documentation and conservation. We have modified parts of the text to better convey this point, for example: "… concerns about the limited personnel, resources, and time available to examine the collected material."

Kind regards, Paul

>> Reviewer #2 attached file comments:

L69 Upon reading this carefully - I think this sets paragraph sets up categories that with further investigation are not really that clearly definned, my suggestion would be to cut it completely - with associated discussion also going.

---Response: One of the aims of this paragraph is to define these concepts, which we believe we have done. We have, however, removed the associated discussion as suggested, while retaining the introductory part.

L73 Move this clause up in the sentence

---Response: We have moved the clause up as suggested.

--- Before it read: Integrative taxonomy emerged to accommodate and unify new concepts and methods from various disciplines to ensure the proper description of taxa, taxonomic stability over time, and preventing taxonomic inflation by integrating multiple lines of evidence [18, 20].

--- Now it reads: Integrative taxonomy, by integrating multiple lines of evidence, emerged to accommodate and unify new concepts and methods from various disciplines to ensure the proper description of taxa, maintain taxonomic stability over time, and prevent taxonomic inflation [18, 20].

L75 I think that in practice there are a complicated set of trade-offs here - I have skinks know only from a single specimen that I could describe, but with accessing comparative material and look at specimens in museums my diagnoses will be less useful, and my descriptions lacking information on distribution are also less useful. So basic message is yes there might be short-cuts, but they have consequences - hence why most people are still to varying degrees doing speciecs descriptions that take a long time

---Response: We completely agree that omitting collected specimens is a trade-off, and that describing a species based on a single specimen is suboptima. However, fast-track taxonomy suggests that examining very large series of specimens (sometimes exceeding 200 individuals, as seen in some descriptions included in this study) may not be necessary or useful—particularly when those specimens are distributed across multiple institutions, making such revisions logistically challenging.

L78 I will also say the descriptions often don't take that long - it is amassing sufficient and compelling evidence that different species are involved and working ouut how to diagnose them - there is no fast track to this I am afraid

---Response: Fast track taxonomy involves (or avoids) several steps that can streamline the species description process, as detailed in Riedel et al. (2013), which we cite multiple times. We would like to emphasize that we are not suggesting that not following a fast track approach necessarily results in major delays. We have reviewed the text to ensure that this point cannot be misinterpreted. Our intention is simply to note that, under a fast-track framework, the description process (sensu stricto, as in our paper), can be more straightforward.

L89 I think this is a poorly explaining dichomtomy - increasingly many species can only be identified with genetics and distribution, even those discovered by integrative approaches. I appreciate that you are saying ID based on barcode data alone is not enough, but as worded the comparison does not work.

---Response: Here we refer to the classical (but not the only) example of turbo taxonomy from Sharkey et al. (2021), in which the authors provided only the barcode sequence as the species diagnosis for 403 species. We believe that such descriptions make it impossible to identify a species without a molecular lab.

L193 maybe emphasise that these are probably best considered estimates for tropical regions

---Response: We agree that these values have been calculated in tropical regions. We selected tropical areas primarily to obtain a sufficiently large sample size (N) for analysis. Although we did not conduct a systematic analysis of non-tropical species, a few recent examples indicate that description times outside the tropics fall within a similar range: *Pelodytes atlanticus* (Portugal, 2021) — 39 years; *Bufotes cypriensis* (Cyprus, 2019) — 8 years since first collection; *Bufo eichwaldi* (Azerbaijan, 2008) — 7 years; *Pyxicephalus beylelli* (Namibia, 2024) — 15 years; and *Phrynomantis newtoni* (Angola, 2021) — 118 years.

L338 don't fully understand these two sentences, as worded they seem to contradict each other - should these two sentences be merged - maybe describe direction of relationship first, and then note the is only significant in two instances

---Response: We have rephrased following the reviewer's suggestion.

--- Before it read: The use of molecular data in species descriptions had a significant effect in the global model and in India but not in Ecuador, Madagascar, or Melanesia. In all regions except Madagascar, incorporating genetic data was associated with longer description times (S7 Fig, S4 Table).

--- Now it reads: Incorporating molecular data was associated with longer description times in all regions except Madagascar, but this effect was significant only in the global model and in India (S7 Fig, S4 Table).

L415 Melanesian and Madagascar also have poorer education infrastructure - i.e Uni's etc

---Response: That is true, and perhaps this partly explains why relatively few local taxonomists are emerging from these two regions.

L428 suggest reword - when you are dealing with 200 undescribed species the desire is there, it is just not feasible to sort everything

---Response: We have rephrased following the reviewer's suggestion.

--- Before it read: This suggests that new species are not always recognized as such in the field, or, when they are, their formal description is not prioritized..

--- Now it reads: This suggests that new species are not always recognized as such in the field, or, when they are, their formal description may be delayed by the limited resources and capacity of (the few) taxonomists to process the sheer volume of undescribed species, or because other research priorities are often more academically rewarded.

L429 I think could cut both this sentence and the previous

---Response: We have rephrased these sentences (see above).

L443 This paragraph feels like a bit of walk through lots of different ideas. I would sugegst try and focus it more one or two key points - my suggestion would a) we need to collection and b) the critical importance of musuems. the fast track taxonomy discussion is unconvincing for reasons mentioned above.

---Response: We have removed the discussion on fast track taxonomy and focused on these points.

L453 but in only two analyses was this relationship significant

---Response: We acknowledge this point; however, our statement specifically refers to the global analysis, in which the relationship was significant.

L455 wording in confusing here - but I think this is key point - at least for Melanesia the easy species are getting described quickly based on morph and calls, the hard species are taking longer and need genetics.

---Response: We partially agree with this point. However, in regions such as Madagascar or India, where 100% species descriptions include genetics, not all of the new described species are cryptic.

L531 this is arguable - if people really need to get the paper they can usually just email the author

---Response: We have deleted the full sentence.

L538 Is this acronym definned before here

---Response: We have deleted the acronym and included the whole wording instead

L543 looking at genbank as an example - I think making sure best as possible data is included in the description is probably a better ideas! Genbank IDs almos never get updated

---Response: We understand the reviewer's concern and agree that ensuring high-quality data in species descriptions is important. However, given the time required, for example, to obtain comprehensive distributional data or assess intraspecific variability for a single species, we believe that prioritizing rapid descriptions—based on sufficient but not exhaustive data—may be more effective during the current biodiversity crisis. Additional information can be incorporated once the species has been described. At the end, a species cannot generally be considered for conservation until it has been formally described.

We assume the reviewer is referring to the taxonomic names or metadata associated with GenBank entries. Unfortunately, updates to GenBank records depend on the submitting party and can only be made by them or by GenBank staff with written authorization. This limitation often prevents corrections or updates once the data are published.

L555 I really don't think setting this up is a simple as you think, and in practice if I need data form a previous authors description I just email the author. Sometime they are willing to share, sometimes they are not - an the latter situation platforms like this will not solve those issues

---Response: Unless journals require standardized data deposition as a mandatory condition for publication—similar to how genetic sequences must be submitted to GenBank prior to publication.

L557 Is this talking about frogs specifically, all animals, all species - there is huge scope here?

---Response: The idea is generally applicable, while acknowledging that different organismal groups would require distinct standards.

L569 again be careful here - this makes it sound like bumbling taxonomists are unaware of the issue - usually they are well aware, and are desperate to get this information as quickly as possible, but various constraints get in the way - quite often not having a job

---Response: We have rephrased this sentence for greater clarity to avoid any suggestion that taxonomists are unaware of the issue.

---Before it read: In some cases, taxonomists describe species whose existence has been known (to specialists) for decades, but because formal naming can be delayed by multiple external constraints, when they finally name them, they sadly realize that the locality where it was first collected has been completely destroyed

---Now it reads: Because of the multiple constraints that can delay formal naming, taxonomists often describe species that have been identified as such for decades. In some cases, given the alarming rate of habitat destruction, it happens that by the time a name is finally given, the type locality has been severely altered or lost.

L574 No - the biggest challenge is massive habitat loss, but this issue is compounded by the lack of recognition

---Response: We have rephrased.

--- Before it read: Therefore, the biggest challenge faced by undescribed species is…

--- Now it reads: Therefore, a major challenge faced by undescribed species is…

>> Additional changes made by authors

L535 – We have added a reference (already cited elsewhere in the manuscript) to support this statement, reference 53.

L557 – After submitting the revised manuscript, we recalled about a pilot submission template for authors suggested in reference 53. We have now included the following sentence "…(see a proposed pilot submission template for taxonomic data: [53])."
